# Mechanosynthesis of Odd‐Numbered Tetraaryl[*n*]cumulenes

**DOI:** 10.1002/anie.201905670

**Published:** 2019-07-17

**Authors:** Karen J. Ardila‐Fierro, Carsten Bolm, José G. Hernández

**Affiliations:** ^1^ Institute of Organic Chemistry RWTH Aachen University Landoltweg 1 52074 Aachen Germany

**Keywords:** ball milling, calcium carbide, cumulenes, mechanochemistry

## Abstract

A mechanochemical synthesis of one‐dimensional carbon allotrope carbyne model compounds, namely tetraaryl[*n*]cumulenes (*n*=3, 5) was realized. Central for the mechanosynthesis of the cumulenic carbon nanostructures were the development of a mechanochemical Favorskii alkynylation‐type reaction and the implementation of a solvent‐free, acid‐free reductive elimination with tin(II) chloride by ball milling.

The use of mechanical energy to facilitate chemical reactions and physical transformations has been rapidly gaining terrain in various fields of the chemical sciences.^1^ One of the most distinct benefits of mechanochemical syntheses by ball milling is the possibility to generate unique product compositions compared to solution‐based protocols.^2^ This alteration in chemical reactivity has been identified to be, at least partially, a consequence of the exertion of mechanical forces under solvent‐free conditions, or the use of only catalytic amounts of organic solvents.^3^, ^4^ Thus, new avenues to react chemicals that differ in their solubility profiles were opened,^5^ for which finding a common reaction medium would be otherwise problematic. Such advantages by mechanochemistry have proven particularly valuable in areas of research focused on the synthesis and functionalization of poorly soluble two‐ and three‐dimensional carbon‐based materials. Examples include the iconic mechanochemical dimerization of C_60_,^6^ the development of mechanically based procedures for the delamination of graphite,^7^ the mechanochemical functionalization of graphene nanosheets,^8^ and the recent mechanosynthesis of nanographenes,^9^ to name just a few.6b Conversely, mechanochemical synthesis or functionalization of one‐dimensional carbon nanostructures, such as carbyne model compounds have remained unexplored.

For practical reasons such as accessibility, stability, and simplicity, polyynes and cumulenes have become ideal model compounds to predict and to study the expected physicochemical properties of carbyne (Scheme 1 a).^10^ In the case of cumulenes, various synthetic methods have been established to produce or to connect sp‐hybridized carbons into cumulenic structures of various lengths, [3]cumulenes being the simplest examples and therefore the most investigated ones.10b, 11 Commonly, [3]cumulenes are synthesized by a two‐step route involving the addition of a Li‐ or Mg‐acetylide to ketones in solution, followed by reductive elimination of the corresponding 1,4‐butyndiols using KI/H_2_SO_4_, SnCl_2_/AcOH, or SnCl_2_/HCl in organic solvents (Scheme 1 b).10b, 11 Although widely applied, these strategies are not universal and become impractical if the cumulene precursors exhibit poor solubility in organic solvents or when acid‐labile substituents decorate the structure of the starting materials and products.

**Scheme 1:**
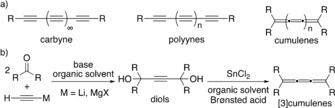
a) Carbyne, polyynes, and cumulenes. b) The most common solution‐based approach towards the synthesis of [3]cumulenes.

With these precedents in mind, we wondered if the stability of [*n*]cumulenes would allow the establishment of a mechanochemical route to access such unique carbon structures. Herein, we report a proof‐of‐principle study demonstrating the realization of this concept.

As starting point, we envisioned that a mechanochemical activation of benzophenone (**1 a**) and solid calcium carbide (CaC_2_)^12^ in the ball mill would enable the mechanosynthesis of propargylic diol **2 a** through a double alkynylation reaction. Previous attempts to use CaC_2_ as a surrogate of acetylene in solution‐based alkynylation reactions have afforded mostly propargyl alcohols such as **3 a** after the ethynylation of carbonylic compounds.^13^, ^14^ However, our recent findings on the mechanochemical copper(I)‐catalyzed A^3^ coupling with calcium carbide revealed that activation of CaC_2_ under solvent‐free and water‐free conditions by mechanochemistry principally harnessed the reactivity of the acetylide ion (C_2_^−2^) from CaC_2_,^3^ rather than following the common approach of producing gaseous acetylene from CaC_2_ and water.^12^ Initial experiments, milling **1 a** and CaC_2_ (1:2 ratio) for 3 h at 800 rpm in a planetary ball mill did not promote any chemical transformation, and only **1 a** was recovered after the workup of the reaction mixture. Then, a series of additives were tested with aim of facilitating the activation of CaC_2_, for example, through a mechanochemical salt metathesis reaction.^15^ Experimentally, CaC_2_ and the corresponding additives (KX; X=F, Cl, Br, I or K_2_CO_3_), were milled for 30 min prior the addition of **1 a**, followed by a second milling period of 3 h. Analysis of the reaction mixture revealed that only KI and K_2_CO_3_ had led to formation of the expected products **2 a** and **3 a**, albeit in trace amounts (for details, see the Supporting Information). Next, KOH was used as an additive to favor a Favorskii alkynylation‐type reaction.^16^ Pleasingly, this time, the solvent‐free mechanochemical reaction afforded propargylic diol **2 a** and propargyl alcohol **3 a** in 41 % yield and 25 % yield, respectively (Scheme 2).^17^

**Scheme 2:**
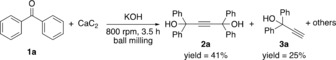
Mechanochemical alkynylation reaction between **1 a** and CaC_2_.

The use of NaOH, LiOH, or Ca(OH)_2_ instead of KOH led to a drop in reactivity (for details, see the Supporting Information), ruling out the basicity of the additive as the main contributor for the chemical reactivity observed by ball milling. Instead, this indicates that the driving force of the reaction might rather be associated with the relative free energies of formation and lattice energies of the additives.^18^

Having established a mechanochemical route to access propargylic diol **2 a**, we then focused on the synthesis of [3]cumulene **4 a**.^19^ As mentioned above, typically, reductive elimination of propargylic diols is carried out using tin(II) chloride (SnCl_2_ or SnCl_2_⋅2 H_2_O) in organic solvents such as diethyl ether or THF, commonly in the presence of Brønsted acids.^20^ Along these lines, in a recent work, Yamago and co‐workers reported that the mixture of SnCl_2_ and HCl generated the ate complex H_2_SnCl_4_, which has been credited to serve as a more active reducing agent formed in situ during the reaction.^21^ However, Brønsted acid‐free Sn‐mediated reductive eliminations are also known.^20^ Collectively, these observations made us curious to see whether a solvent‐free, acid‐free mechanochemical Sn^II^‐mediated reduction of diol **2 a** in the ball mill would lead to the formation of [3]cumulene **4 a** (Scheme 3 a). To test this idea, **2 a** and SnCl_2_⋅2 H_2_O (1:1.1 ratio) were reacted for 1 h in a planetary ball mill. At the end of the milling, a bright yellow solid reaction mixture was observed. Analysis of this solid by powder X‐ray diffraction (PXRD) techniques revealed the disappearance of Bragg reflections corresponding to **2 a** and the presence of a new set of reflections that resembled the expected PXRD pattern of [3]cumulene **4 a** (Scheme 3 b). Isolation of **4 a** from the inorganic residue was carried out by dissolving **4 a** in a minimal amount of organic solvent, and by passing such solution through a pad of Celite (a similar yield was obtained after purification of **4 a** by column chromatography or extraction). Repeating the reaction using anhydrous SnCl_2_ under inert atmosphere gave similar results, thereby ruling out a potential formation of catalytic amounts of HCl from water present in SnCl_2_⋅*x* H_2_O. Interestingly, carrying out the mechanochemical reaction using a ratio **2 a**/SnCl_2_⋅2 H_2_O (1:0.5) led to the formation of [3]cumulene **4 a** together with 1,1,4,4‐tetraphenyl‐2‐chlorobuta‐2,3‐dien‐1‐ol (**4 a′)** (Scheme 3 c) (**4 a**:**4 a′** ca. 1:1 ratio indicated by ^1^H NMR spectroscopy at RT). The identity of **4 a′** was confirmed by various analytic techniques and by independent synthesis.22a Moreover, milling independently synthesized **4 a′** and SnCl_2_⋅2 H_2_O led to the formation of [3]cumulene **4 a**, together with the concomitant formation of unidentified byproducts (Scheme 3 d); thereby indicating that compounds like chloroallene **4 a′** could correspond to some of the long sought‐after intermediates in reductive elimination of diols with stannous chloride (for a plausible mechanism, see the Supporting Information).^20^, ^21^, ^23^

**Scheme 3:**
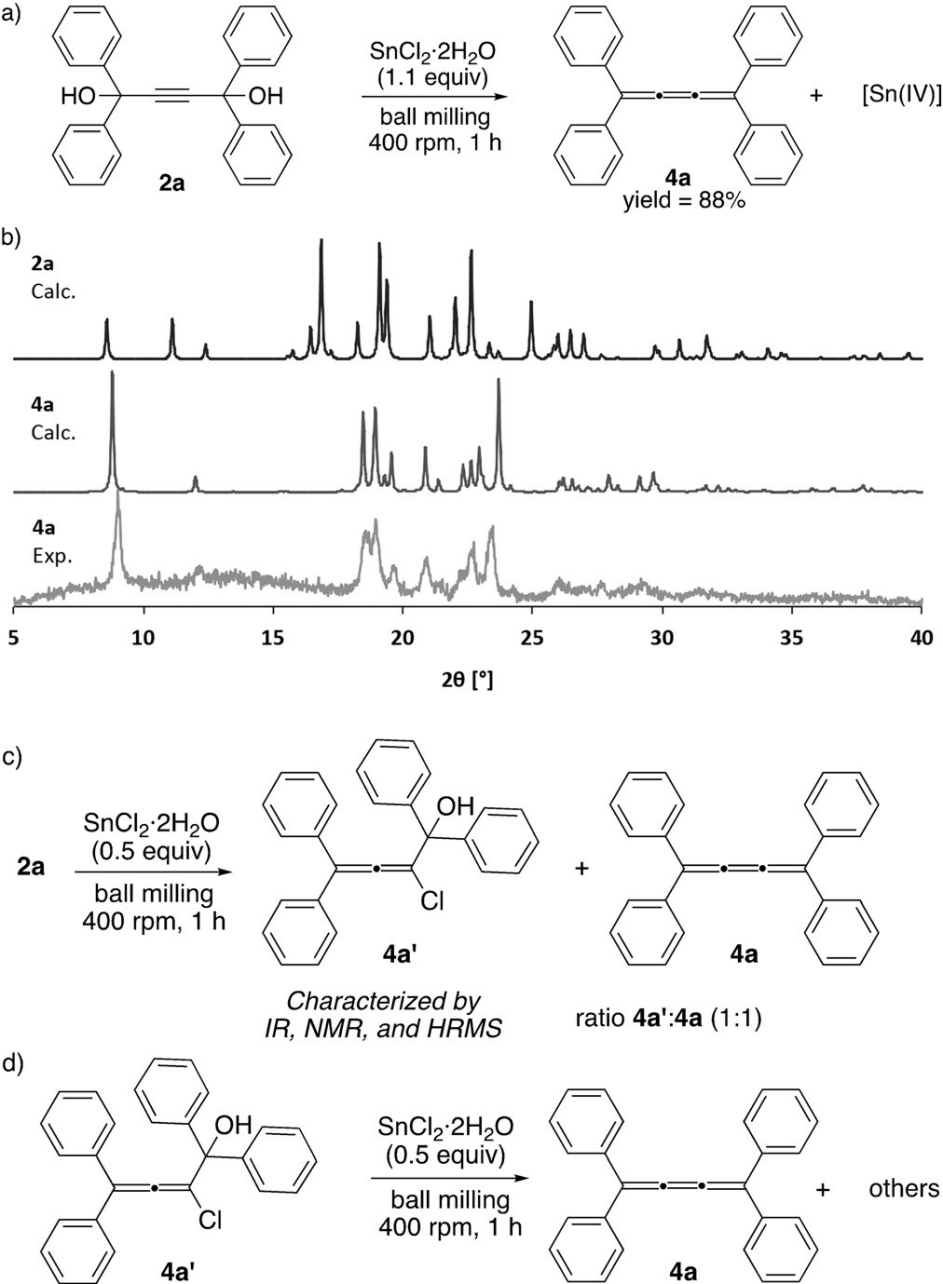
a) Mechanochemical reductive elimination of **2 a**. b) PXRD patterns for: **2 a** (top),24a **4 a** (middle),24b solid recovered after the reaction of **2 a** and SnCl_2_⋅2 H_2_O (1.1 equiv) (bottom). c) Reductive elimination of **2 a** using 0.5 equiv of SnCl_2_⋅2 H_2_O. d) Use of **4 a′** in the synthesis of **4 a**.

Next, using the established ball milling conditions for the mechanochemical Favorskii alkynylation‐type reaction, a series of ketones **1 a**–**j**, calcium carbide, and KOH were reacted (Scheme 4). Gratifyingly, benzophenone derivatives bearing electron‐donating (**1 b**–**c**) and electron‐withdrawing groups (**1 d**–**g**) tolerated the mechanochemical protocol (Scheme 4). Similarly, unsymmetrical ketones such as phenyl 2‐pyridyl ketone (**1 h**), 4‐fluorobenzophenone (**1 i**), and *tert*‐butyl phenyl ketone (**1 j**) also underwent the mechanochemical alkynylation reactions affording the corresponding 2‐butyne‐1,4‐diols **2 h**–**j** and propargyl alcohols **3 h**–**j** (Scheme 4).

**Scheme 4:**
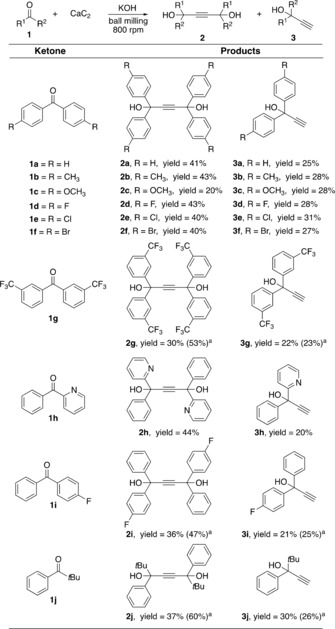
Mechanosynthesis of propargylic diols **2 a**–**j** and propargyl alcohols **3 a**–**j** using calcium carbide.^[a]^ Yields in parenthesis correspond to five‐fold scale‐up experiments using a 45 mL ZrO_2_ milling vessel.

With a library of mechanochemically synthesized 2‐butyne‐1,4‐diols **2** at our disposal, we selected a few additional representative examples to test the mechanochemical SnCl_2_‐mediated reductive elimination. Pleasingly, the application of the ball milling protocol to **2 b** afforded the expected [3]cumulene **4 b** in 82 % yield (Scheme 5). The use of halogen substituted aryl alkynediol **2 d** led to the formation of [3]cumulene **4 d** in 75 % yield (Scheme 5). Additionally, unsymmetrical alkynediol **2 i** reacted smoothly to provide the *Z* and *E* isomers **4 i** (ratio 1:1 indicated by ^19^F NMR spectroscopy at RT) in 87 % yield (Scheme 5).19c On the contrary, milling 2‐pyridyl substituted alkynediol **2 h** with SnCl_2_⋅2 H_2_O afforded a complex mixture of unidentified products.^25^ Similarly, less reactive alkyl‐ and aryl‐substituted diol **2 j**, failed to undergo the reductive elimination using SnCl_2_⋅2 H_2_O by ball milling. Although this time, ketone **2 j** remained unreactive. In general, synthesis of alkyl substituted [*n*]cumulenes is more difficult compared to aryl[*n*]cumulenes.10b Moreover, stability of cumulenes increases with the use of steric bulk end groups.^11^

**Scheme 5:**
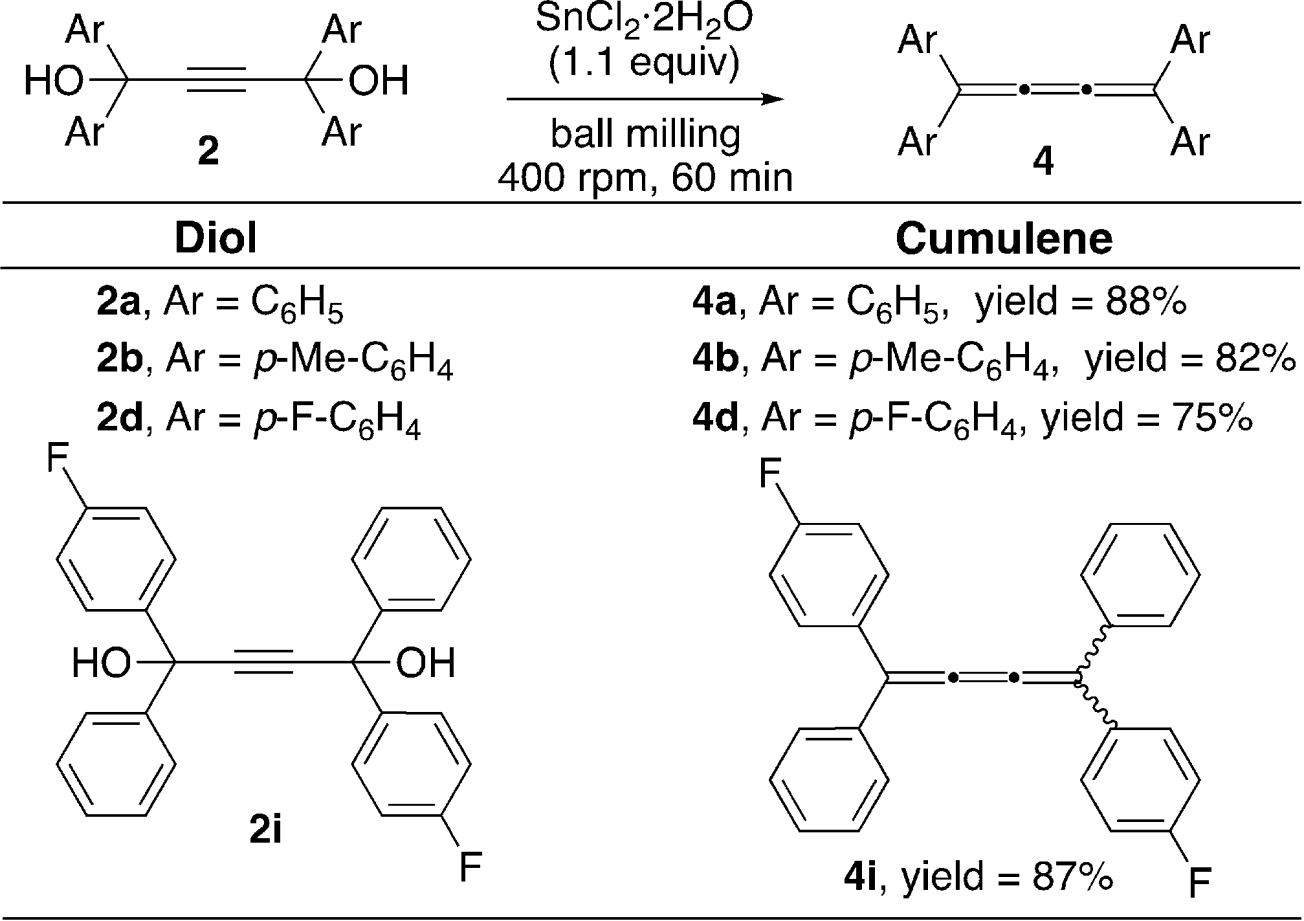
Synthesis of [3]cumulenes **4**.

Having demonstrated not only the usefulness of the mechanochemically synthesized 2‐butyne‐1,4‐diols **2** but also the relative stability of [3]cumulenes in the ball mill, we decided to use propargyl alcohol **3 a** as a building block for the mechanosynthesis of the much more interesting [5]cumulenic unit.10b Thus, **3 a** was reacted in the presence of CuCl and tetramethylethylenediamine (TMEDA) through a mechanochemical copper‐mediated oxidative coupling to afford hexa‐2,4‐diyne‐1,6‐diol (**5 a**) in 88 % yield (Scheme 6).^26^

**Scheme 6:**
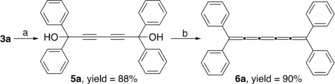
Mechanosynthesis of [5]cumulene **6 a**. a) CuCl (1.0 equiv), TMEDA (1.0 equiv), 800 rpm, 120 min. b) SnCl_2_⋅_2_ H_2_O (1.0 equiv), 400 rpm, 60 min.

Then, solid **5 a** was milled in the presence of SnCl_2_⋅2 H_2_O for 60 min at 400 rpm in a planetary ball mill to give a dark red solid. Immediate analysis of the crude reaction mixture by solution NMR spectroscopy confirmed the formation of [5]cumulene **6 a** upon milling. Isolation of **6 a** was accomplished after dissolving **6 a** in a minimal amount of organic solvent, and by passing such solution through a pad of Celite to give **6 a** in 90 % yield (Scheme 6).

In summary, we have developed a mechanochemical route to access tetraaryl[*n*]cumulenes (*n*=3, 5) by implementation of ball milling techniques. The synthetic approach towards tetraaryl[3]cumulenes **4** encompasses the mechanochemical activation of ketones, calcium carbide, and potassium hydroxide through a Favorskii alkynylation‐type reaction in a ball mill, followed by the establishment of a solventless and acid‐free SnCl_2_‐mediated reductive elimination of propargylic diols **2**. Particularly interesting was the observation that mechanochemical reductive elimination of **2 a** with sub‐stoichiometric amounts of SnCl_2_⋅2 H_2_O enabled the isolation of one intermediate of the reaction, thereby gaining insight into the mechanism of the transformation. Additionally, the mechanochemical approach was successfully extended to the synthesis of the illustrative tetraphenyl[5]cumulene (**6 a**) after a mechanochemical copper‐mediated oxidative coupling of propargylic alcohol **3 a** followed by reductive elimination of diyne diol **5 a** with SnCl_2_⋅2 H_2_O. From a more general perspective, we foresee the protocols reported in this work to be applicable in areas such as material sciences for the mechanosyntheses of carbon‐based materials from CaC_2_,^27^ and in supramolecular chemistry for the mechanosynthesis of, for example, conjugated polycyclic hydrocarbons and cumulenes derived from poorly soluble starting materials or from acid‐labile precursors.

## Conflict of interest

The authors declare no conflict of interest.

## Supporting information

As a service to our authors and readers, this journal provides supporting information supplied by the authors. Such materials are peer reviewed and may be re‐organized for online delivery, but are not copy‐edited or typeset. Technical support issues arising from supporting information (other than missing files) should be addressed to the authors.

Supplementary

## References

[1] For selected recent reviews and examples, see:

[1a] C. Bolm , J. G. Hernández , Angew. Chem. Int. Ed. 2019, 58, 3285–3299; 10.1002/anie.201810902 30417972

[1b] D. Tan , F. García , Chem. Soc. Rev. 2019, 48, 2274–2292; 30806391 10.1039/c7cs00813a

[1c] K. Lisac , F. Topíc , M. Arhangelskis , S. Cepić , P. A. Julien , C. W. Nickels , A. J. Morris , T. Friščić , D. Cinčić , Nat. Commun. 2019, 10, 61; 30610194 10.1038/s41467-018-07957-6 PMC6320372

[1d] N. Willis-Fox , E. Rognin , T. A. Aljohani , R. Daly , Chem 2018, 4, 2499–2537;

[1e] M. J. Muñoz-Batista , D. Rodriguez-Padron , A. R. Puente-Santiago , R. Luque , ACS Sustainable Chem. Eng. 2018, 6, 9530–9544;

[1f] C. Bolm , J. G. Hernández , ChemSusChem 2018, 11, 1410–1420; 29436773 10.1002/cssc.201800113

[1g] J. Andersen , J. Mack , Green Chem. 2018, 20, 1435–1443;

[1h] M. Ferguson , M. S. Moyano , G. A. Tribello , D. E. Crawford , E. M. Bringa , S. L. James , M. G. Del Pópolo , Chem. Sci. 2019, 10, 2924–2929; 30996870 10.1039/c8sc04971h PMC6427933

[1i] N. R. Rightmire , T. P. Hanusa , Dalton Trans. 2016, 45, 2352–2362; 26763151 10.1039/c5dt03866a

[1j] P. Baláž , M. Achimovičová , M. Baláž , P. Billik , A. Cherkezova-Zheleva , J. M. Criado , F. Delogu , E. Dutková , E. Gaffet , F. L. Gotor , R. Kumar , I. Mitov , T. Rojac , M. Senna , A. Streletskii , K. Wieczorek-Ciurowa , Chem. Soc. Rev. 2013, 42, 7571–7637; 23558752 10.1039/c3cs35468g

[1k] E. Boldyreva , Chem. Soc. Rev. 2013, 42, 7719–7738. 23864028 10.1039/c3cs60052a

[2a] J. G. Hernández , C. Bolm , J. Org. Chem. 2017, 82, 4007–4019; 28080050 10.1021/acs.joc.6b02887

[2b] J.-L. Do , T. Friščić , ACS Cent. Sci. 2017, 3, 13–19; 28149948 10.1021/acscentsci.6b00277 PMC5269651

[2c] J. C. Robertson , M. L. Coote , A. C. Bissember , Nat. Chem. Rev. 2019, 3, 290–304;

[2d] K. Budny-Godlewski , I. Justyniak , M.-K. Leszczyński , J. Lewiński , Chem. Sci. DOI: 10.1039/c9sc01396b. 10.1039/c9sc01396b PMC6686643 31588281

[3] M. Turberg , K. J. Ardila-Fierro , C. Bolm , J. G. Hernández , Angew. Chem. Int. Ed. 2018, 57, 10718–10722; 10.1002/anie.201805505 29897672

[4] J. L. Howard , M. C. Brand , D. L. Browne , Angew. Chem. Int. Ed. 2018, 57, 16104–16108; 10.1002/anie.201810141 PMC6282732 30335216

[5] Z.-Y. Zhang , D. Ji , W. Mao , Y. Cui , Q. Wang , L. Han , H. Zhong , Z. Wei , Y. Zhao , K. Nørgaard , T. Li , Angew. Chem. Int. Ed. 2018, 57, 10949–10953; 10.1002/anie.201805998 29952136

[6a] K. Komatsu , G.-W. Wang , Y. Murata , M. Shiro , Nature 1997, 387, 583–586;

[6b] S.-E. Zhu , F. Li , G.-W. Wang , Chem. Soc. Rev. 2013, 42, 7535–7570. 23677148 10.1039/c3cs35494f

[7a] J. M. González-Domínguez , V. León , M. I. Lucío , M. Prato , E. Vázquez , Nat. Protoc. 2018, 13, 495–506; 29446772 10.1038/nprot.2017.142

[7b] V. J. González , A. M. Rodríguez , V. León , J. Frontiñán-Rubio , J. L. Fierro , M. Durán-Prado , A. B. Muñoz-García , M. Pavone , E. Vázquez , Green Chem. 2018, 20, 3581–3592.

[8a] I.-Y. Jeon , Y.-R. Shin , G.-J. Sohn , H.-J. Choi , S.-Y. Bae , J. Mahmood , S.-M. Jung , J.-M. Seo , M.-J. Kim , D. W. Chang , L. Dai , J.-B. Baek , Proc. Natl. Acad. Sci. USA 2012, 109, 5588–5593; 22454492 10.1073/pnas.1116897109 PMC3326447

[8b] I.-Y. Jeon , H. J. Choi , S. M. Jung , J. M. Seo , M. J. Kim , L. Dai , J. B. Baek , J. Am. Chem. Soc. 2013, 135, 1386–1393; 23110522 10.1021/ja3091643

[8c] X. Fan , D. W. Chang , X. Chen , J.-B. Baek , L. Dai , Curr. Opin. Chem. Eng. 2016, 11, 52–58.

[9] S. Grätz , D. Beyer , V. Tkachova , S. Hellmann , R. Berger , X. Feng , L. Borchardt , Chem. Commun. 2018, 54, 5307–5310. 10.1039/c8cc01993b 29651492

[10a] W. A. Chalifoux , R. R. Tykwinski , Nat. Chem. 2010, 2, 967–971; 20966954 10.1038/nchem.828

[10b] J. A. Januszewski , R. R. Tykwinski , Chem. Soc. Rev. 2014, 43, 3184–3203; 24671214 10.1039/c4cs00022f

[10c] W. Xu , E. Leary , S. Hou , S. Sangtarash , M. T. González , G. Rubio-Bollinger , Q. Wu , H. Sadeghi , L. Tejerina , K. E. Christensen , N. Agraït , S. J. Higgins , C. J. Lambert , R. J. Nichols , H. L. Anderson , Angew. Chem. Int. Ed. 2019, 58, 8378–8382; 10.1002/anie.201901228 PMC6563095 31026371

[11] For recent review articles, see:

[11a] L. Leroyer , V. Maraval , R. Chauvin , Chem. Rev. 2012, 112, 1310–1343; 22040177 10.1021/cr200239h

[11b] D. Wendinger , R. R. Tykwinski , Acc. Chem. Res. 2017, 50, 1468–1479. 28561581 10.1021/acs.accounts.7b00164

[12] For recent reviews on application of CaC_2_ as a surrogate of acetylene in chemical synthesis, see:

[12a] K. S. Rodygin , Y. A. Vikenteva , V. P. Ananikov , ChemSusChem 2019, 12, 1483–1516; 30938099 10.1002/cssc.201802412

[12b] V. V. Voronin , M. S. Ledovskaya , A. S. Bogachenkov , K. S. Rodygin , V. P. Ananikov , Molecules 2018, 23, 2442; 10.3390/molecules23102442 PMC6222752 30250005

[12c] K. S. Rodygin , G. Werner , F. A. Kucherov , V. P. Ananikov , Chem. Asian J. 2016, 11, 965–976. 26898248 10.1002/asia.201501323

[13a] A. Hosseini , D. Seidel , A. Miska , P. R. Schreiner , Org. Lett. 2015, 17, 2808–2811. 25997788 10.1021/acs.orglett.5b01219

[14] For a report on the use of CaC_2_ in the synthesis of propargylic diols after long reaction times in benzene, see: G. F. Woods , L. H. Schwartzman , Org. Synth. 1952, 32, 70–72.

[15] I. R. Speight , S. C. Chmely , T. P. Hanusa , A. L. Rheingold , Chem. Commun. 2019, 55, 2202–2205. 10.1039/c8cc10155h 30702108

[16a] M. B. Smith , J. March , March's Advanced Organic Chemistry: Reactions, Mechanisms, and Structure, 6th ed., Wiley-Interscience, Hoboken, 2007, ch. 16, 1359–1360;

[16b] A. E. Favorskii , M. P. Skosarevskii , Zh. Russ. Khim. O-va. 1900, 32, 652;

[16c] E. Y. Shmidt , I. A. Bidusenko , N. I. Protsuk , A. I. Mikhaleva , B. A. Trofimov , Russ. J. Org. Chem. 2013, 49, 8–11.

[17] **2 a** and **3 a** were obtained concomitantly with formation of by-products from a background reaction between **1 a** and KOH.

[18] Relative free energies of formation of LiOH (−439.0 kJ mol^−1^), NaOH (−379.5 kJ mol^−1^), KOH (−379.1 kJ mol^−1^), Ca(OH)_2_ (−898.6 kJ mol^−1^), CaC_2_ (−64.8 kJ mol^−1^). Lattice energy of LiOH (−1021.0 kJ mol^−1^); NaOH (−885.2 kJ mol^−1^); KOH (−789.3 kJ mol^−1^). For a reference, see: A. I. Aleixo , P. H. Olivera , H. P. Diogo , M. E. Minas de Piedade , Thermochim. Acta 2005, 428, 131–136.

[19] For selected examples, see:

[19a] S. N. Spisak , M. U. Bühringer , Z. Wei , Z. Zhou , R. R. Tykwinski , M. A. Petrukhina , Angew. Chem. Int. Ed. 2019, 58, 2023–2028; 10.1002/anie.201812283 30560557

[19b] N. Kerisit , P. Gawel , B. Levandowski , Y.-F. Yang , V. García-López , N. Trapp , L. Ruhlmann , C. Boudon , K. N. Houk , F. Diederich , Chem. Eur. J. 2018, 24, 159–168; 29139153 10.1002/chem.201703903

[19c] M. U. Bühringer , K. Padberg , M. D. Phelps , H. Maid , C. Placht , C. Neiss , M. J. Ferguson , A. Görling , R. R. Tykwinski , Angew. Chem. Int. Ed. 2018, 57, 8321–8325; 10.1002/anie.201802137 29603858

[19d] Y. Kuwatani , G. Yamamoto , M. Oda , M. Iyoda , Bull. Chem. Soc. Jpn. 2005, 78, 2188–2208.

[20] J. L. Marshall , D. Lehnherr , B. D. Lidner , R. R. Tykwinski , ChemPlusChem 2017, 82, 967–1001. 10.1002/cplu.201700168 31961601

[21] V. K. Patel , E. Kayahara , S. Yamago , Chem. Eur. J. 2015, 21, 5742–5749. 25753916 10.1002/chem.201406650

[22a] F. Toda , N. Ooi , K. Akagi , Bull. Chem. Soc. Jpn. 1971, 44, 1050–1054;

[22b] E. V. Banide , B. C. Molloy , Y. Ortin , H. Müller-Bunz , M. J. McGlinchey , Eur. J. Org. Chem. 2007, 2611–2622.

[23] M. S. Newman , K. Kanakarajan , J. Org. Chem. 1980, 45, 2301–2304.

[24a] J. K. Sundar , K. M. Kumar , V. Vijayakumar , J. Suresh , S. Natarajan , P. L. N. Lakshman , Acta Crystallogr. Sect. E 2010, 66, o679; 10.1107/S160053681000629X PMC2983558 21580424

[24b] V. G. Jiménez , R. Tapia , M. A. Medel , I. F. A. Mariz , T. Ribeiro , V. Blanco , J. M. Cuerva , E. Maçôas , A. G. Campaña , Chem. Commun. 2018, 54, 3359–3362. 10.1039/c8cc00386f 29542798

[25] Reductive elimination of **2 h** using SnCl_2_ in Et_2_O-HCl has been reported to fail, leading instead to the formation of a pyridylquinolizinone derivative:

[25a] G. R. Newkome , J. D. Sauer , M. L. Erbland , J. Chem. Soc. Chem. Commun. 1975, 885–886.

[26] For reports on mechanochemical oxidative couplings of terminal acetylenes, see:

[26a] R. Schmidt , R. Thorwirth , T. Szuppa , A. Stolle , B. Ondruschka , H. Hopf , Chem. Eur. J. 2011, 17, 8129–8138; 21626591 10.1002/chem.201100604

[26b] L. Chen , B. E. Lemma , J. S. Rich , J. Mack , Green Chem. 2014, 16, 1101–1103.

[27a] Y. Li , Q. Liu , W. Li , H. Meng , Y. Lu , C. Li , ACS Appl. Mater. Interfaces 2017, 9, 3895–3901; 28071888 10.1021/acsami.6b13610

[27b] M. E. Casco , S. Kirchhoff , D. Leistenschneider , R. Rauche , E. Brunner , L. Borchardt , Nanoscale 2019, 11, 4712–4718. 30838363 10.1039/c9nr01019j

