# Supplementary material for: Mechanosynthesis of Odd‐Numbered Tetraaryl[n]cumulenes
Source: Angew Chem Int Ed Engl. 2019 Jul 17;58(37):12945–9. doi: 10.1002/anie.201905670 (PMC6773223; doi:10.1002/anie.201905670)
Supplement: Supplementary file 1 — Supplementary [file ANIE-58-12945-s001.pdf]

CHEMISTRY
AN ASIAN JOURNAL

Supporting Information

Three-Dimensional Hierarchical Constructs of MOF-on-Reduced Graphene Oxide for Lithium–Sulfur Batteries

Yushan Wu,[a] Haoqing Jiang,[c] Fu-Sheng Ke,*[a] and Hexiang Deng*[a, b]

asia_201900848_sm_miscellaneous_information.pdf

Supporting Information

Table of contents

1. General information S2

2. Table 1 Optimization of the milling conditions for the mechanochemical Favorskii reaction S3

3. Mechanochemical Favorskii reaction S3

4. Mechanosynthesis of [3]cumulenes 4 S4

4.1 Plausible mechanism for the formation of 4a involving intermediate 4a' S4

5. Mechanochemical homocoupling of 3a S5

6. Mechanosynthesis of [5]cumulene 6a S5

7. Characterization of the products S5

8. NMR spectra S21

9. References S52

1. General information

All chemicals are commercial available and they were used as received unless otherwise stated. Calcium carbide was purchased from Alfa Aesar® (technical grade CaC_2, 74.24 % purity). Thin-layer chromatography (TLC) was performed using TLC plates (silica gel 60 on aluminum or glass with fluorescence indicator F254) from MERCK. Qualitative analysis of the TLC plates was carried out using UV light (λ = 254 nm and λ = 366 nm) and/or by immersion in an aqueous solution of potassium permanganate ($KMnO_4$) and heating of the stained plates with a heat-gun at 300 °C until dryness. Products were purified by column chromatography using silica gel 60 (40−63 μm) from ACROS Organics. Solvents for column chromatography were distilled prior to use.
Mechanochemical reactions were carried out in a in a FRITSCH planetary micro mill model “Pulverisette 7 classic line”.
All NMR spectra were recorded on a VNMRS 400 or on a VNMRS 600 spectrometer. Proton chemical shifts are reported in parts per million on the δ scale and are calibrated using the residual non-deuterated solvent signal as an internal reference. Spectral data is provided as follows: chemical shift in ppm (from downfield to upfield), multiplicity (s = singlet, d = doublet, m = multiplet), integration and coupling constant *J*.
IR-spectra were recorded on a PERKIN ELMER 100 FT/IR spectrometer with an ATR-Unit.
Mass spectra were recorded on a FINNIGAN SSQ7000 (EI 70 eV) spectrometer and high-resolution mass spectra on a THERMO FISHER Scientific Orbitrap XL spectrometer.
Powder X-Ray Diffraction (PXRD) patterns were collected on a Bruker D2 phaser X-ray diffractometer.

2. Table S1 Optimization of the milling conditions for the mechanochemical Favorskii reaction[a]

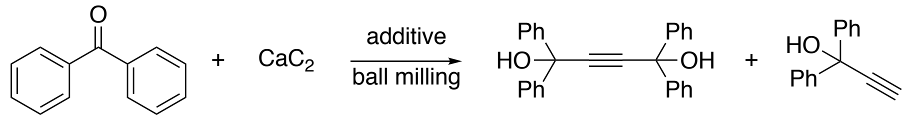

Entry	Additive	Yield (%)[c] **1a**	Yield (%)[c] **2a**	Yield (%)[c] **3a**
1[b]	none	100	0	0
2	KI	97	2	1
3	K_2CO_3	78	14	8
**4**[d]	**KOH**	**14**[e]	**41**[e]	**25**[e]
5	NaOH	89	7	4
6	LiOH	100	0	0
7	$Ca(OH)_2$	100	0	0

[a] Reaction conditions: CaC_2 (70.4 mg, 1.098 mmol) (of technical grade CaC_2, 74.24 % purity, real amount added 0.815 mmol) and the additive (2.196 mmol) were milled under argon atmosphere for 30 min at 800 rpm in a planetary mill using a 12 mL ZrO_2 vessel with 20 ZrO_2 balls of 5 mm in diameter. Then, **1a** (100 mg, 0.549 mmol) was added into the milling jar under argon atmosphere and the milling was restarted for 3 h. [b] **1a** and CaC_2 (1:2 ratio) were milled for 3 h without the additive. [c] Determined by ^{1}H NMR spectroscopy. [d] Formation of by-products from the reaction between **1a** and KOH was observed (ca. 10 %). [e] Determined after separation of the products by column chromatography.

3. Mechanochemical Favorskii reaction

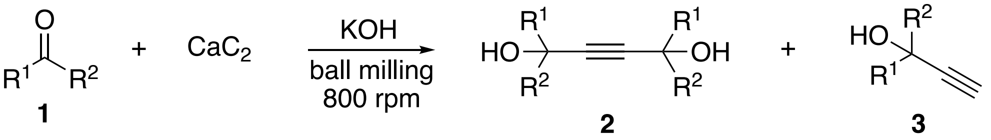

Inside a glovebox, CaC_2 (70.4 mg, 1.098 mmol) (of technical grade CaC_2, 74.24 % purity, real amount used 0.815 mmol) and KOH (123.16 mg, 2.19 mmol) were added in a 12 mL ZrO_2 milling jar with 20 ZrO_2 balls of 5 mm. The jar was closed tightly, sealed with electrical tape, taken out of the glovebox, and milled for 30 min at 800 rpm. The jar, still taped, was placed again inside the glovebox, there, it was opened and **1** (0.54 mmol) was added. The jar was closed and as mentioned before, electrical tape was used to seal it. Once the container was outside the glovebox, the mixture was milled for 3 h at 800 rpm. After the milling was stopped, the milling jar was open outside the glovebox and HCl 1M (10 mL) was added.

Then, the aqueous phase was extracted with ethyl acetate (3 × 15 mL). The organic fraction was concentrated under vacuum and the product was purified by flash column chromatography on silica gel using a gradient of ethyl acetate in *n*-pentane.

Five-fold scale up experiments were carried out in a 45 mL ZrO_2 milling jar with 5 ZrO_2 balls of 10 mm, and 14 ZrO_2 balls of 5 mm, under otherwise identical conditions.

4. Mechanosynthesis of [3]cumulenes

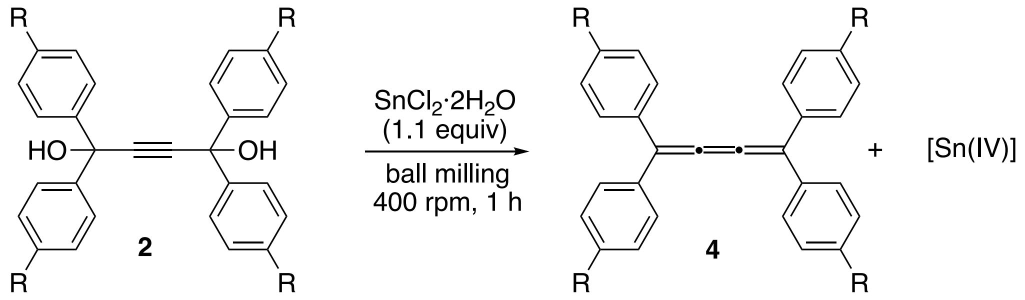

A mixture of **2** (0.12 mmol, 1.0 equiv) and $SnCl_2 \cdot 2H_2O$ (31.8 mg, 0.14 mmol, 1.1 equiv), were transferred into a ZrO_2 milling jar (12 mL volume) equipped with 20 ZrO_2 balls of 5 mm and milled at 400 rpm for 60 min. After the milling was stopped, the reaction mixture was dissolved in a minimal amount of dichloromethane and filtered through a pad of Celite® to remove the inorganic residue. Alternatively, extraction using water (20 mL) and ethyl acetate (3 × 15 mL) or purification by flash column chromatography on silica gel using a gradient of ethyl acetate in *n*-pentane also led to the isolation of cumulenes **4** in similar yields.

4.1 Plausible mechanism for the formation of 4a involving intermediate 4a'

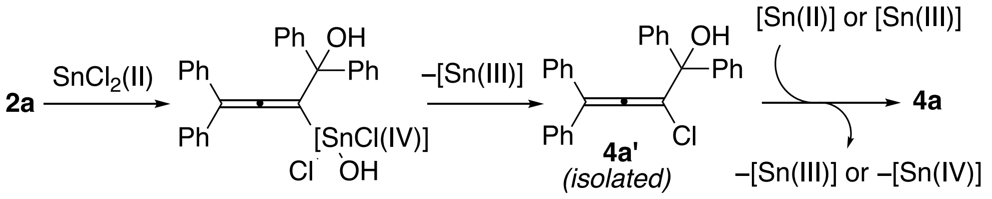

5. Mechanochemical homocoupling of 3a

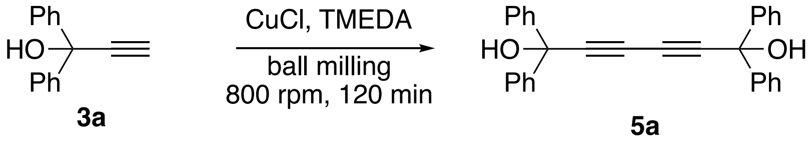

A mixture of **3a** (100 mg, 0.48 mmol, 1.0 equiv), CuCl (47.6 mg, 0.48 mmol, 1.0 equiv) and TMEDA (55.8 mg, 0.48 mmol, 1.0 equiv), were milled in a 12 mL ZrO_2 milling jar with 20 ZrO_2 balls of 5 mm. Once the milling was stopped, the reaction mixture was recovered and extracted with water (20 mL) and ethyl acetate (3 × 15 mL). Then the organic phase was concentrated in vacuum and the product was purified by flash column chromatography on silica gel using a gradient of ethyl acetate in *n*-pentane.

6. Mechanosynthesis of [5]cumulene 6a

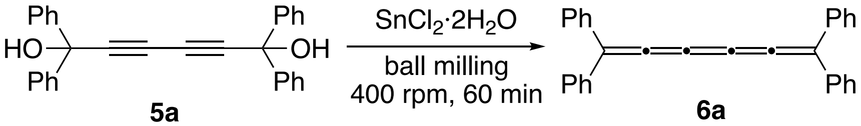

A mixture of **5a** (50 mg, 0.12 mmol, 1.0 equiv) and $SnCl_2 \cdot 2H_2O$ (32.6 mg, 0.14 mmol, 1.2 equiv), were transferred into a ZrO_2 milling jar (12 mL volume) equipped with 20 ZrO_2 balls of 5 mm and milled at 400 rpm for 60 min. After the milling was stopped, the reaction mixture was dissolved in a minimal amount of dichloromethane and filtered through a pad of Celite® to remove the inorganic residue.

7. Characterization of the products

1,1,4,4-Tetraphenylbut-2-yne-1,4-diol (2a)

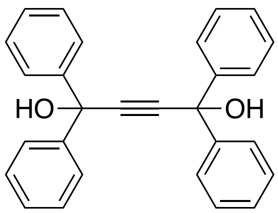

2a

Molecular formula: $C_{28}H_{22}O_2$

Molecular Mass: 390.1620 g/mol

^{1}H NMR (400 MHz, acetone-d_6), δ (ppm): 7.66 (d, *J* = 7.58 Hz, 8H), 7.29 (t, *J* = 7.65 Hz, 8H), 7.21 (t, *J* = 7.41 Hz, 4H), 5.82 (brs, 2H).

^{13}C{^{1}H} NMR (100 MHz, acetone-d_6), δ (ppm): 147.3, 128.7, 128.0, 126.8, 90.5, 74.5. The spectral data for this compound match that reported in the literature.[3]

IR (ATR) ν (cm^{-1}) = 3323, 3063, 3025, 2109, 1691, 1596, 1488, 1450, 1258, 1211, 1006, 694.

MS (EI$^+$, 70 eV) *m/z* (%): 372.2 (30) [M−H_2O]$^+$, 285.1 (45), 267.0 (75), 207.1 (15), 183.1 (15), 165.1 (9), 105.1 (100), 77.1 (21).

HRMS (ESI$^+$) m/z: calcd. for [M+Na]$^+$ = [$C_{28}H_{22}O_2Na$]$^+$: 413.1512; found: 413.1524.

1,1-Diphenyl-prop-2-yn-1-ol (3a)

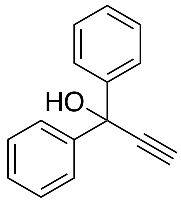

3a

Molecular formula: $C_{15}H_{12}O$

Molecular Mass: 208.0888 g/mol

^{1}H NMR (400 MHz, acetone-d_6), δ (ppm): 7.64 (d, *J* = 7.54 Hz, 4H), 7.32 (t, *J* = 7.63 Hz, 4H), 7.24(t, *J* = 7.37 Hz, 2H), 5.67 (s, 1H), 3.35 (s, 1H).

^{13}C{^{1}H} NMR (100 MHz, acetone-d_6), δ (ppm): 146.9, 128.7, 128.0, 126.7, 87.9, 76.2, 74.2. The spectral data for this compound match that reported in the literature.[4]

IR (ATR) ν (cm^{-1}) = 3551, 3061, 3028, 2115, 1597, 1488, 1448, 1327, 1202, 1155, 992, 688.

MS (EI$^+$, 70 eV) *m/z* (%): 208.1 (100) [M]$^+$, 207.1 (40), 189.0 (18), 179.0 (28), 131.0 (34), 130.1 (26), 105.1 (8), 77.1 (14), 53.3 (24).

HRMS (ESI$^+$) m/z: calcd. for [M+Na]$^+$ = [$C_{15}H_{12}ONa$]$^+$: 231.0780; found: 231.0780.

1,1,4,4-Tetrakis(4-methylphenyl)but-2-yne-1,4-diol (2b)

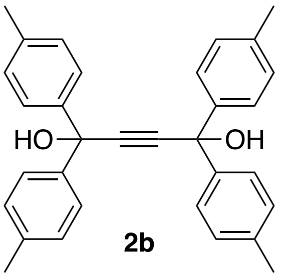

2b

Molecular formula: $C_{32}H_{30}O_2$
Molecular Mass: 446.2246 g/mol
^{1}H NMR (600 MHz, acetone-d_6), δ (ppm): 7.53 (d, *J* = 8.49 Hz, 8H), 7.10 (d, *J* = 8.08 Hz, 8H), 5.63 (s, 2H), 2.28 (s, 12H).
^{13}C{^{1}H} NMR (150 MHz, acetone-d_6), δ (ppm): 144.7, 137.3, 129.2, 126.8, 90.4, 74.2, 20.9. The spectral data for this compound match that reported in the literature.[4]

IR (ATR) ν (cm^{-1}) = 3386, 3022, 2918, 2324, 2187, 1698, 1506, 1445, 1411, 1216,1005, 676.

MS (EI$^+$, 70 eV) *m/z* (%): 446.4 (2) $[M]^+$, 428.3 (18), 327.1 (40), 309.1 (81), 211.1 (18), 119.1 (100), 91.2. (22), 65.3 (5).
HRMS (ESI$^+$) m/z: calcd. for $[M+Na]^+$ = $[C_{32}H_{30}O_2Na]^+$: 469.2138; found: 469.2139.

1,1-Bis(4-methylphenyl)prop-2-yn-1-ol (3b)

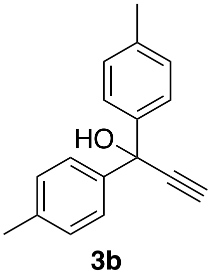

3b

Molecular formula: $C_{17}H_{16}O$
Molecular Mass: 236.1201 g/mol
^{1}H NMR (600 MHz, acetone-d_6), δ (ppm): 7.49 (d, *J* = 8.49 Hz, 4H), 7.12 (d, *J* = 8.35 Hz, 4H), 5.52 (s, 1H), 3.30 (s, 1H), 2.28 (s, 6H).
^{13}C{^{1}H} NMR (150 MHz, acetone-d_6), δ (ppm): 144.2, 137.4, 129.2, 126.7, 88.2, 75.8, 75.8, 73.9, 20.9. The spectral data for this compound match that reported in the literature.[5]

IR (ATR) ν (cm^{-1}) = 3539, 3286, 2922, 2325, 2183, 1508, 1450, 1409, 1322, 1171,

986, 815.

MS (EI$^+$, 70 eV) *m/z* (%): 236.1 (100) $[M]^+$, 221.1 (55), 219.1 (40), 178.0 (15), 145.1 (31), 119.1 (11), 115.1 (11), 91.2 (16), 53.3 (15).
HRMS (ESI$^+$) m/z: calcd. for $[M+Na]^+$ = $[C_{17}H_{16}ONa]^+$: 259.1093; found: 259.1097.

1,1,4,4-Tetrakis(4-methoxyphenyl)but-2-yne-1,4-diol (2c)

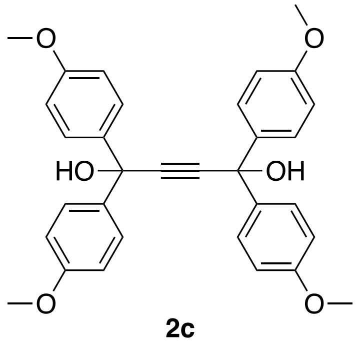

2c

Molecular formula: $C_{32}H_{30}O_6$
Molecular Mass: 510.2042 g/mol
^{1}H NMR (600 MHz, acetone-d_6), δ (ppm): 7.53 (d, J = 8.83 Hz, 8H), 6.84 (d, J = 8.82 Hz, 8H), 5.56 (s, 2H), 3.76 (s, 12H).
^{13}C{^{1}H} NMR (150 MHz, acetone-d_6), δ (ppm): 159.7, 139.8, 128.1, 113.9, 90.5, 73.8, 55.5, 55.5. The spectral data for this compound match that reported in the literature.[3]

IR (ATR) ν (cm^{-1}) = 3442, 3001, 2935, 2837, 2549, 2187, 2045, 1979, 1893, 1705, 1605, 1504, 1459, 1299, 1243, 1169, 1027, 902, 827.

MS (EI$^+$, 70 eV) *m/z* (%): 510.5 (4) $[M]^+$, 456.3 (10), 408.1 (11), 357.1 (16), 268.1 (58), 242.1 (38), 135.1 (100), 123.1 (30), 77.3 (24).
HRMS (ESI$^+$) m/z: calcd. for $[M+Na]^+$ = $[C_{32}H_{30}O_6Na]^+$: 533.1934; found: 533.1934.

1,1-Bis(4-methoxyphenyl)prop-2-yn-1-ol (3c)

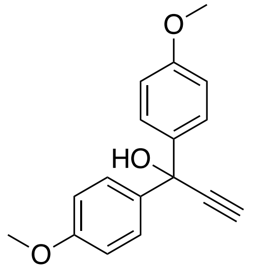

3c

Molecular formula: $C_{17}H_{16}O_3$

Molecular Mass: 268.1099 g/mol

^{1}H NMR (600 MHz, acetone-d_6), δ (ppm): 7.49 (d, J = 8.69 Hz, 4H), 6.86 (d, J = 8.82 Hz, 4H), 5.42 (s, 1H), 3.77 (s, 6H), 3.29 (s, 1H).

^{13}C{^{1}H} NMR (150 MHz, acetone-d_6), δ (ppm): 159.8, 139.3, 128.0, 113.9, 88.4, 75.7, 73.6, 55.4. The spectral data for this compound match that reported in the literature.[5]

IR (ATR) ν (cm^{-1}) = 3855, 3471, 3243, 3077, 2935, 2839, 2482, 2324, 2187, 2103, 1982, 1928, 1747, 1607, 1505, 1461, 1347, 1244, 1060, 817, 684.

MS (EI^{+}, 70 eV) *m/z* (%): 268.1 (100) $[M]^{+}$, 251.1 (58), 237.1 (17), 161.1 (38), 135.1 (21), 108.2 (14), 77.3 (10), 53.4 (24).

HRMS (ESI^{+}) m/z: calcd. for $[M+Na]^{+}$ = $[C_{17}H_{16}O_{3}Na]^{+}$: 291.0991; found: 291.0991.

1,1,4,4-Tetrakis(4-fluorophenyl)but-2-yne-1,4-diol (2d)

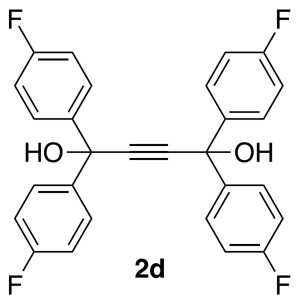

2d

Molecular formula: $C_{28}H_{18}F_{4}O_{2}$

Molecular Mass: 462.1243 g/mol

^{1}H NMR (600 MHz, acetone-d_6), δ (ppm): 7.69-7.66 (m, 8H), 7.11-7.07 (m, 8H), 6.07 (s, 2H).

^{13}C{^{1}H} NMR (150 MHz, acetone-d_6), δ (ppm): 162.8 (d, ^{1}J(C,F) = 245.4 Hz), 143.0 (d, ^{4}J(C,F) = 2.9 Hz), 128.8 (d, ^{3}J(C,F) = 8.3 Hz), 115.5 (d, ^{2}J(C,F) = 21.6 Hz), 90.4, 73.6.

^{19}F NMR (564 MHz, acetone-d_6), δ (ppm): −116.82.

The spectral data for this compound match that reported in the literature.[3]

IR (ATR) ν (cm^{-1}) = 3553, 3446, 3070, 2924, 2855, 2669, 2187, 2103, 1903, 1777, 1661, 1601, 1500, 1414, 1343, 1221, 1156, 1095, 994, 827.

MS (EI^{+}, 70 eV) *m/z* (%): 462.2 (1) $[M]^{+}$, 444.2 (15), 339.1 (32), 321.1 (40), 219.1 (13), 123.0 (100), 95.1 (14).

HRMS (ESI^{+}) m/z: calcd. for $[M+Na]^{+}$ = $[C_{28}H_{18}F_{4}O_{2}Na]^{+}$: 485.1135; found: 485.1136.

1,1-Bis(4-fluorophenyl)prop-2-yn-1-ol (3d)

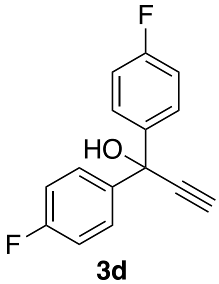

3d

Molecular formula: $C_{15}H_{10}F_2O$
Molecular Mass: 244.0700 g/mol
^{1}H NMR (600 MHz, $CDCl_3$), δ (ppm): 7.57-7.54 (m, 4H), 7.04-7.01 (m, 4H), 2.90 (s, 1H), 2.81 (s, 1H).
^{13}C{^{1}H} NMR (150 MHz, $CDCl_3$), δ (ppm): 162.5 (d, ^1J(C,F) = 246.8 Hz), 140.2 (d, ^4J(C,F) = 3.6 Hz), 127.9 (d, ^3J(C,F) = 8.1 Hz), 115.3 (d, ^2J(C,F) = 21.7 Hz), 86.0, 76.1, 73.5.
^{19}F NMR (564 MHz, $CDCl_3$), δ (ppm): −114.39.
The spectral data for this compound match that reported in the literature.[4]

IR (ATR) ν (cm^{-1}) = 3567, 3418, 3298, 3073, 2927, 2703, 2115, 1987, 1898, 1771, 1601, 1503, 1411, 1326, 1225, 1158, 1054, 987, 832.

MS (EI$^+$, 70 eV) *m/z* (%): 244.1 (100) $[M]^+$, 243.0 (34), 227.1 (37), 215.0 (24), 149.0 (53), 148.0 (40), 123.0 (25), 95.1 (19), 53.2 (34).
HRMS (ESI$^+$) m/z: calcd. for $[M]^+$ = $[C_{15}H_{10}F_2O]^+$: 244.0694; found: 244.0691.

1,1,4,4-Tetrakis(4-chlorophenyl)but-2-yne-1,4-diol (2e)

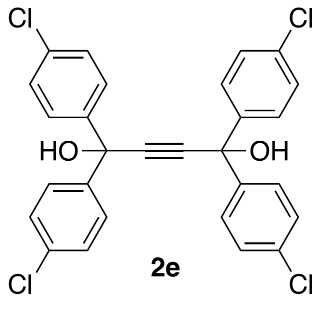

2e

Molecular formula: $C_{28}H_{18}Cl_4O_2$
Molecular Mass: 526.0061 g/mol
^{1}H NMR (400 MHz, acetone-d_6), δ (ppm): 7.65 (br. d, J = 8.70 Hz, 8H), 7.36 (br. d, J = 8.90 Hz, 8H), 6.17 (s, 2H).

^{13}C{^{1}H} NMR (100 MHz, acetone-d_6), δ (ppm): 145.5, 133.7, 129.0, 128.5, 90.1, 73.6. The spectral data for this compound match that reported in the literature.[3]

IR (ATR) ν (cm^{-1}) = 3378, 2925, 2854, 2286, 2180, 2013, 1905, 1786, 1691, 1588, 1485, 1402, 1210, 1174, 1135, 1902, 902, 819.

MS (EI^{+}, 70 eV) *m/z* (%): 510.3 (4), 508.2 (3) $[M-H_2O]^{+}$, 475.2 (7), 387.0 (14), 369.0 (10), 250.9 (10), 141.0 (33), 139.0 (100), 111.1 (17), 75.2 (5).
HRMS (ESI^{+}) m/z: calcd. for $[M+Na]^{+}$ = $[C_{28}H_{18}Cl_4O_2Na]^{+}$: 548.9953; found: 548.9952.

1,1-Bis(4-chlorophenyl)prop-2-yn-1-ol (3e)

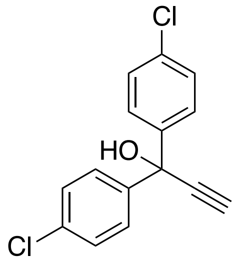

3e

Molecular formula: $C_{15}H_{10}Cl_2O$
Molecular Mass: 276.0109 g/mol
^{1}H NMR (400 MHz, acetone-d_6), δ (ppm): 7.63 (br. d, *J* = 8.81 Hz, 4H), 7.36 (br. d, *J* = 8.58 Hz, 4H), 5.98 (s, 1H), 3.45 (s, 1H).
^{13}C{^{1}H} NMR (100 MHz, acetone-d_6), δ (ppm): 145.4, 133.7, 128.9, 128.5, 86.8, 77.1, 73.4.
The spectral data for this compound match that reported in the literature.[5]

IR (ATR) ν (cm^{-1}) = 3552, 3409, 3294, 2925, 2319, 2114, 1904, 1782, 1694, 1590, 1486, 1401, 1325, 1169, 1091, 986, 822.

MS (EI^{+}, 70 eV) *m/z* (%): 277.9 (24), 276.0 (39) $[M]^{+}$, 241.0 (100), 189.0 (14), 165.0 (36), 139.0 (13), 111.0 (11), 75.2 (9).
HRMS (ESI^{+}) m/z: calcd. for $[M]^{+}$ = $[C_{15}H_{10}Cl_2O]^{+}$: 276.0103; found: 276.0091.

1,1,4,4-Tetrakis(4-bromophenyl)but-2-yne-1,4-diol (2f)

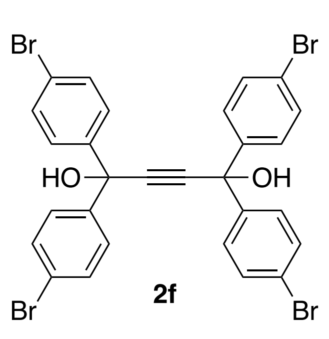

2f

Molecular formula: $C_{28}H_{18}Br_4O_2$
Molecular Mass: 701.8040 g/mol
^{1}H NMR (600 MHz, acetone-d_6), δ (ppm): 7.58 (d, J = 8.84 Hz, 8H), 7.51 (d, J = 8.72 Hz, 8H), 6.20 (brs, 2H).
^{13}C{^{1}H} NMR (150 MHz, acetone-d_6), δ (ppm): 145.9, 132.0, 128.8, 121.9, 90.0, 73.7. The spectral data for this compound match that reported in the literature.[3]

IR (ATR) ν (cm^{-1}) = 3380, 3087, 2958, 2701, 2297, 2166, 2045, 1925, 1803, 1698, 1584, 1482, 1359, 1228, 1193, 1071, 1007, 903, 813.

MS (EI$^+$, 70 eV) *m/z* (%): 689.0 (1), 607.0 (3), 442.0 (7), 340.0 (27), 285.0 (25), 185.0 (100), 183.0 (96), 157.0 (21), 76.3 (13).
HRMS (ESI$^+$) m/z: calcd. for $[M+Na]^+$ = $[C_{28}H_{18}Br_4O_2Na]^+$: 724.7932; found: 724.7933.

1,1-Bis-(4-bromophenyl)prop-2-yn-1-ol (3f)

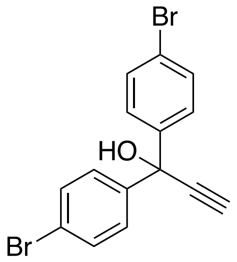

3f

Molecular formula: $C_{15}H_{10}Br_2O$
Molecular Mass: 363.9098 g/mol
^{1}H NMR (600 MHz, acetone-d_6), δ (ppm): 7.57-7.55 (m, 4H), 7.54-7.51 (m, 4H), 6.00 (s, 1H), 3.47 (s, 1H).
^{13}C{^{1}H} NMR (150 MHz, acetone-d_6), δ (ppm): 145.8, 132.0, 128.8, 121.9, 86.7, 77.1, 73.5. The spectral data for this compound match that reported in the literature.[7]

IR (ATR) ν (cm^{-1}) = 3941, 3555, 3289, 3063, 2924, 2856, 2659, 2304, 2113, 1908, 1794, 1584, 1480, 1397, 1357, 1291, 1177, 1123, 1064, 990, 815.

MS (EI$^+$, 70 eV) *m/z* (%): 367.9 (32), 365.9 (63), 364.0 (35) $[M+H]^+$, 287.0 (88), 285.0 (100), 210.9 (34), 206.1 (43), 189.0 (23), 178.1 (37), 155.0 (9), 76.2 (11), 53.3 (44).
HRMS (ESI$^+$) m/z: calcd. for $[M]^+$ = $[C_{15}H_{10}Br_2O]^+$: 363.9092; found: 363.9101.

1,1,4,4-Tetrakis(3-(trifluoromethyl)phenyl)but-2-yne-1,4-diol (2g)

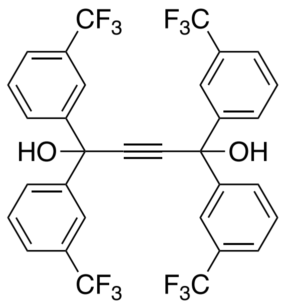

2g

Molecular formula: $C_{32}H_{18}F_{12}O_2$
Molecular Mass: 662.1115 g/mol
^{1}H NMR (600 MHz, acetone-d_6), δ (ppm): 8.04 (s, 4H), 7.94 (d, *J* = 7.81 Hz, 4H), 7.66 (d, *J* = 8.07 Hz, 4H), 7.61 (t, *J* = 8.07 Hz, 4H), 6.60 (s, 2H).
^{13}C{^{1}H} NMR (150 MHz, acetone-d_6), δ (ppm): 147.4, 131.0 (q, ^2J(C,F) = 32.2 Hz), 130.8, 130.3, 125.5 (q, ^3J(C,F) = 3.6 Hz), 125.0 (q, ^1J(C,F) = 271.0 Hz), 123.1 (q, ^2J(C,F) = 3.80 Hz), 90.2, 73.9.
^{19}F NMR (376 MHz, acetone-d_6), δ (ppm): −63.16.

IR (ATR) ν (cm^{-1}) = 3396, 2931, 2155, 1907, 1661, 1694, 1614, 1489, 1441, 1324, 1163, 1122, 1073, 903, 803.

MS (EI$^+$, 70 eV) *m/z* (%): 644.4 (1) [M−H_2O]$^+$, 643.4 (5), 489.3 (18), 319.0 (9), 173.0 (100), 145.1 (17).
HRMS (ESI$^+$) m/z: calcd. for [M+Na]$^+$ = [$C_{32}H_{18}F_{12}O_2Na$]$^+$: 685.1007; found: 685.1005.

1,1-Bis(3-(trifluoromethyl)phenyl)prop-2-yn-1-ol (3g)

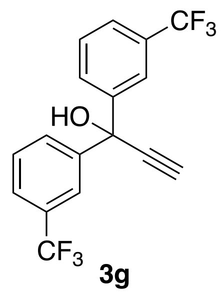

3g

Molecular formula: $C_{17}H_{10}F_6O$
Molecular Mass: 344.0636 g/mol
^{1}H NMR (600 MHz, acetone-d_6), δ (ppm): 8.03 (s, 2H), 7.90 (d, *J* = 7.79 Hz, 2H), 7.67-7.61 (m, 4H), 6.35 (s, 1H), 3.59 (s, 1H).

^{13}C{^{1}H} NMR (150 MHz, acetone-d_6), δ (ppm): 147.4, 130.9, 130.8 (q, ^{2}J(C,F) = 32.4 Hz), 130.3, 125.4 (q, ^{3}J(C,F) = 4.1 Hz), 125.1 (q, ^{1}J(C,F) = 272.7 Hz), 123.1 (q, ^{3}J(C,F) = 3.99 Hz), 86.2, 78.0, 73.6.
^{19}F NMR (564 MHz, acetone-d_6), δ (ppm): −63.15

IR (ATR) ν (cm^{-1}) = 3608, 3307, 3076, 2929, 2659, 2328, 1908, 1727, 1613, 1440, 1326, 1164, 1124, 1074, 804, 702.

MS (EI^{+}, 70 eV) *m/z* (%): 344.2 (100) [M]$^{+}$, 327.1 (21), 275.1 (58), 255.1 (13), 227.1 (8), 199.0 (61), 173.0 (17), 145.1 (22), 95.1 (4).
HRMS (ESI^{+}) m/z: calcd. for [M]$^{+}$ = [$C_{17}H_{10}F_6O$]$^{+}$: 344.0630; found: 344.0622.

1,4-Diphenyl-1,4-di(pyridin-2-yl)but-2-yne-1,4-diol (2h)

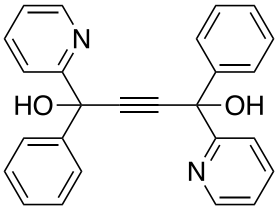

2h

Molecular formula: $C_{26}H_{20}N_2O_2$
Molecular Mass: 392.1525 g/mol
^{1}H NMR (600 MHz, acetone-d_6), δ (ppm): (Mixture of rotamers) 8.56 (s, 2H), 7.83-7.79 (m, 2H), 7.74-7.66 (m, 6H), 7.32-7.29 (m, 6H), 7.26-7.23 (m, 2H), 6.38 (brs, 1H), 6.37 (brs, 1H).
^{13}C{^{1}H} NMR (150 MHz, acetone-d_6), δ (ppm): (Mixture of rotamers) 163.2, 163.1, 148.5, 148.5, 145.9, 138.3, 128.8, 128.8, 128.3, 128.3, 127.2, 123.7, 121.7, 121.6, 89.4, 89.4, 74.6.

IR (ATR) ν (cm^{-1}) = 3411, 3058, 2926, 2656, 2286, 2030, 1981, 1895, 1740, 1587, 1490, 1433, 1362, 1293, 1197, 1141, 1095, 1011, 926, 750.

MS (EI^{+}, 70 eV) *m/z* (%): 392.0 (7) [M]$^{+}$, 376.0 (15), 374.9 (100), 344.9 (12), 295.9 (18), 195.9 (12), 105.0 (15), 78.2 (12).
HRMS (ESI^{+}) m/z: calcd. for [M+Na]$^{+}$ = [$C_{26}H_{20}N_2O_2Na$]$^{+}$: 415.1404; found: 415.1417.

1-Phenyl-1-(pyridin-2-yl)prop-2-yn-1-ol (3h)

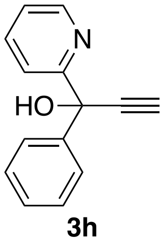

3h

Molecular formula: $C_{14}H_{11}NO$
Molecular Mass: 209.0841 g/mol
^{1}H NMR (600 MHz, acetone-d_6), δ (ppm): 8.54-8.52 (m, 1H), 7.81-7.78 (m, 1H), 7.71-7.69 (m, 2H), 7.62 (d, J = 8.01 Hz, 1H), 7.35-7.33 (m, 2H), 7.32-7.30 (m, 1H), 7.28-725 (m, 1H), 6.41 (brs, 1H), 3.31 (brs, 1H).
^{13}C{^{1}H} NMR (150 MHz, acetone-d_6), δ (ppm): 162.6, 148.3, 145.3, 138.3, 128.8, 128.4,127.0, 123.7, 121.7, 121.5, 87.1, 75.8, 74.2. The spectral data for this compound match that reported in the literature.[8]

IR (ATR) ν (cm^{-1}) = 3263, 3057, 2926, 2855, 2578, 2118, 1956, 1891, 1736, 1664, 1590, 1431, 1365, 1246, 1186, 1153, 1089, 1052, 928, 782.

MS (EI$^+$, 70 eV) *m/z* (%): 209.9 (24), 209.0 (100) $[M]^+$, 179.9 (41), 132.0 (14), 106.0 (7), 78.2 (17).
HRMS (ESI$^+$) m/z: calcd. for $[M+Na]^+$ = $[C_{14}H_{11}NO]^+$: 210.0919; found: 210.0913.

1,4-Bis(4-fluorophenyl)-1,4-diphenylbut-2-yne-1,4-diol (2i)

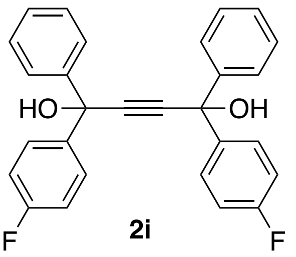

2i

Molecular formula: $C_{28}H_{20}F_2O_2$
Molecular Mass: 426.1431 g/mol
^{1}H NMR (600 MHz, acetone-d_6), δ (ppm): 7.70-7.65 (m, 8H), 7.34-7.31 (m, 4H), 7.26-7.24 (m, 2H), 7.09-7.06 (m, 4H), 5.97 (brs, 1H).
^{13}C{^{1}H} NMR (150 MHz, acetone-d_6), δ (ppm): 162.7 (d, ^1J(C,F) = 244.0 Hz), 146.9, 143.4 (d, ^4J(C,F) = 2.9 Hz), 128.9, 128.8, 128.1, 126.7, 115.4 (d, ^2J(C,F) = 21.7 Hz), 90.4, 74.0.
^{19}F NMR (564 MHz, acetone-d_6), δ (ppm): −117.24.

IR (ATR) ν (cm^{-1}) = 3329, 3066, 2926, 2854, 2470, 2116, 1993, 1899, 1692, 1601, 1502, 1448, 1365, 1226, 1157, 1134, 1096, 1007, 895, 834.

MS (EI$^+$, 70 eV) *m/z* (%): 408.1 (18) $[M-H_2O]^+$, 380.2 (7), 321.1 (18), 303.1 (27), 285.1 (27), 183.0 (12), 123.1 (100), 105.1 (96), 95.2 (20) 77.3 (24).
HRMS (ESI$^+$) m/z: calcd. for $[M+Na]^+$ = $[C_{28}H_{20}F_2O_2Na]^+$: 449.1323; found: 449.1322.

1-(4-Fluorophenyl)-1-phenylprop-2-yn-1-ol (3i)

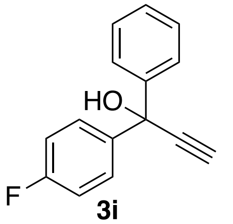

3i

Molecular formula: $C_{15}H_{11}FO$
Molecular Mass: 226.0794 g/mol
^{1}H NMR (600 MHz, acetone-d_6), δ (ppm): 7.66-7.62 (m, 4H), 7.34-7.31 (m, 2H), 7.27-7.24 (m, 1H), 7.10-7.07 (m, 2H), 5.79 (s, 1H), 3.40 (s, 1H).
^{13}C{^{1}H} NMR (150 MHz, acetone-d_6), δ (ppm): 162.7 (d, ^1J(C,F) = 248.0 Hz), 146.6, 143.1 (d, ^4J(C,F) = 3.0 Hz), 128.8, 128.8, 128.7, 128.1, 126.9, 115.3 (d, ^2J(C,F) = 21.6 Hz), 87.7, 76.5 (d, ^3J(C,F) = 5.5 Hz), 73.8.
^{19}F NMR (564 MHz, acetone-d_6), δ (ppm): −117.29.

IR (ATR) ν (cm^{-1}) = 3922, 3549, 3293, 3064, 2926, 2668, 2327, 2114, 1991, 1898, 1769, 1601, 1503, 1448, 1327, 1225, 1160, 1046, 985, 833.

MS (EI$^+$, 70 eV) *m/z* (%): 226.1 (100) $[M]^+$, 209.1 (71), 197.1 (24), 149.0 (31), 130.1 (20), 123.1 (12), 123.1 (100), 105.1 (8), 95.2 (10), 77.3 (12), 53.4 (34).
HRMS (ESI$^+$) m/z: calcd. for $[M+H]^+$ = $[C_{15}H_{12}FO]^+$: 227.0872; found: 227.1255.

2,2,7,7-Tetramethyl-3,6-diphenyloct-4-yne-3,6-diol (2j)

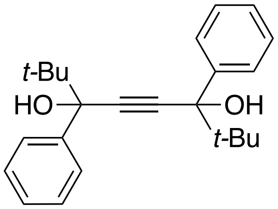

2j

Molecular formula: $C_{24}H_{30}O_2$
Molecular Mass: 350.2246 g/mol
^{1}H NMR (600 MHz, acetone-d_6), δ (ppm): 7.68-7.65 (m, 4H), 7.32-7.28 (m, 4H), 7.27-7.25 (m, 2H), 4.84 (d, *J* = 1.44 Hz, 1H), 4.83 (d, *J* = 1.58 Hz, 1H), 1.05 (s, 9H), 1.04 (s, 9H).
^{13}C{^{1}H} NMR (150 MHz, acetone-d_6), δ (ppm): 144.1, 144.0, 128.8, 128.8, 127.4, 127.4, 89.1, 89.1, 78.7, 40.3, 40.2, 26.0, 26.0.

IR (ATR) ν (cm^{-1}) = 3553, 3457, 2963, 2873, 2287, 2164, 1728, 1601, 1482, 1448, 1217,1033, 983, 703.

MS (EI$^+$, 70 eV) *m/z* (%): 333.2 (19) [M−OH]$^+$, 293.0 (25), 275.1 (40), 236.0 (18), 219.0 (10), 191.0 (12), 105.0 (100), 57.2 (31).
HRMS (ESI$^+$) m/z: calcd. for [M+Na]$^+$ = [$C_{24}H_{30}O_2Na$]$^+$: 373.2138; found: 373.2137.

4,4-Dimethyl-3-phenylpent-1-yn-3-ol (3j)

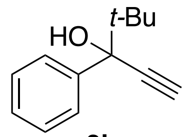

3j

Molecular formula: $C_{13}H_{16}O$
Molecular Mass: 188.1201 g/mol
^{1}H NMR (400 MHz, acetone-*d*$_6$), δ (ppm): 7.64-7.62 (m, 2H), 7.33-7.26 (m, 3H), 4.79 (s, 1H), 3.07 (s, 1H), 1.00 (s, 9H).
^{13}C{^{1}H} NMR (100 MHz, acetone-*d*$_6$), δ (ppm): 143.5, 128.6, 127.8, 127.5, 87.9, 78.5, 74.8, 74.8, 39.7, 25.7.

IR (ATR) ν (cm^{-1}) = 3553, 3469, 3299, 2967, 2319, 2111, 1669, 1601, 1483, 1449, 1216, 1061, 980, 754.

MS (EI$^+$, 70 eV) *m/z* (%): 188.1 (3) [M]$^+$, 173.0 (18), 171.1 (44), 132.1 (81), 131.0 (100), 105.1 (15), 77.2. (14), 57.3 (29).
HRMS (ESI$^+$) m/z: calcd. for [M+Na]$^+$ = [$C_{13}H_{16}ONa$]$^+$: 211.1093; found: 211.1093.

1,1,4,4-Tetraphenylbuta-1,2,3-triene (4a)

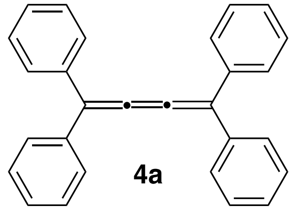

4a

Molecular formula: $C_{28}H_{20}$
Molecular Mass: 356.1565 g/mol
^{1}H NMR (600 MHz, $CDCl_3$), δ (ppm): 7.59-7.56 (m, 8H), 7.41-7.38 (m, 8H), 7.35-7.33 (m, 4H).
^{13}C{^{1}H} NMR (150 MHz, $CDCl_3$), δ (ppm): 152.1, 138.9, 129.6, 128.5, 128.1, 122.8. The spectral data for this compound match that reported in the literature.[5]

IR (ATR) ν (cm^{-1}) = 3385, 3051, 2922, 2854, 2318, 2082, 1991, 1812, 1734, 1588, 1485, 1439, 1286, 1155, 1074, 691.

MS (EI$^+$, 70 eV) *m/z* (%): 357.1 (28), 356.1 $[M]^+$(100), 276.1 (8), 178.0 (23), 176.1 (4), 138.1(2).
HRMS (ESI$^+$) m/z: calcd. for $[M]^+$ = $[C_{28}H_{20}]^+$: 356.1559; found: 356.1568.

2-Chloro-1,1,4,4-tetraphenylbuta-2,3-dien-1-ol (4a')

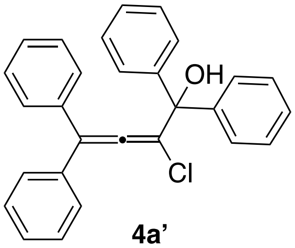

4a'

Molecular formula: $C_{28}H_{21}ClO$
Molecular Mass: 408.1281 g/mol
^{1}H NMR (600 MHz, $CDCl_3$), δ (ppm): 7.44 (dd, *J* = 7.94 Hz, *J* = 1.97 Hz, 4H), 7.33 (t, *J* = 3.15 Hz, 6H), 7.27-7.23 (m, 6H), 7.08-7.07 (m, 4H), 3.35 (s, 1H).
^{13}C{^{1}H} NMR (150 MHz, $CDCl_3$), δ (ppm): 202.2, 143.2, 135.3, 128.9, 128.6, 128.5, 128.1, 128.0, 127.3, 119.1, 112.4, 82.5. The spectral data for this compound match that reported in the literature.[9]

IR (ATR) ν (cm^{-1}) = 3867, 3548, 3059, 2923, 2855, 2320, 2034, 1946, 1811, 1722, 1596, 1491, 1446, 1331, 1163, 1085, 692.

MS (EI$^+$, 70 eV) *m/z* (%): 410.2(4), 408.1 $[M]^+$(12), 372.2 (79), 267.1 (23), 183.1 (53), 105.1(100), 77.2 (26).
HRMS (ESI$^+$) m/z: calcd. for $[M]^+$ = $[C_{28}H_{21}ClONa]^+$: 431.1178; found: 431.1178.

1,1,4,4-Tetrakis(4-methylphenyl)buta-1,2,3-triene (4b)

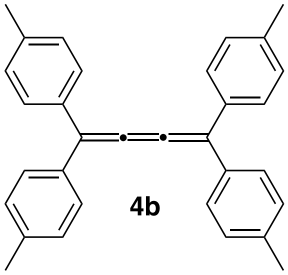

4b

Molecular formula: $C_{32}H_{28}$
Molecular Mass: 412.2191 g/mol
^{1}H NMR (400 MHz, $CDCl_3$), δ (ppm): 7.48 (d, *J* = 8.12 Hz, 8H), 7.20 (d, *J* = 8.04 Hz, 8H), 2.42 (s, 12H).
^{13}C{^{1}H} NMR (100 MHz, $CDCl_3$), δ (ppm): 150.5, 137.8, 136.3, 129.4, 129.2, 121.6, 21.4.

The spectral data for this compound match that reported in the literature.[5]

IR (ATR) ν (cm^{-1}) = 3396, 3025, 2917, 2861, 2325, 2098, 1910, 1801, 1603, 1503, 1408,1291, 1183, 1112, 820.

MS (EI$^+$, 70 eV) *m/z* (%): 413.3 (36), 412.3 $[M]^+$(100), 206.1 (12), 191.1 (4), 176.1 (2).

HRMS (ESI$^+$) m/z: calcd. for $[M]^+$ = $[C_{32}H_{28}]^+$: 412.2185; found: 412.2188.

1,1,4,4-Tetrakis(4-fluorophenyl)buta-1,2,3-triene (4d)

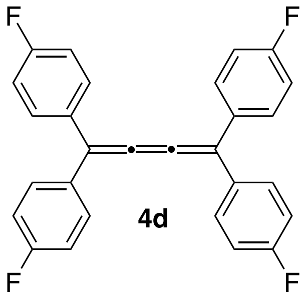

Molecular formula: $C_{28}H_{16}F_4$

Molecular Mass: 428.1188 g/mol

^{1}H NMR (400 MHz, dichloromethane-*d$_2$*), δ (ppm): 7.52-7.48 (m, 8H), 7.13-7.09 (m, 8H).

^{13}C{^{1}H} NMR (100 MHz, dichloromethane-*d$_2$*), δ (ppm): 163.2 (d, ^1J(C,F) = 253.7 Hz), 135.2, 131.3 (d, ^3J(C,F) = 8.1 Hz), 120.8, 116.0 (d, ^2J(C,F) = 21.9 Hz).

^{19}F NMR (376 MHz, dichloromethane-*d$_2$*), δ (ppm): −113.70.

The spectral data for this compound match that reported in the literature.[3]

IR (ATR) ν (cm^{-1}) = 3855, 3748, 2924, 2662, 2323, 2112, 1962, 1892, 1600, 1503, 1407,1231, 1157, 1014, 834.

MS (EI$^+$, 70 eV) *m/z* (%): 429.2 (43), 428.2 $[M]^+$(100), 332.2 (5), 214.1 (33).

HRMS (ESI$^+$) m/z: calcd. for $[M]^+$ = $[C_{28}H_{16}F_4]^+$: 428.1182; found: 428.1175.

(*E/Z*)-1,4-bis(4-fluorophenyl)-1,4-diphenylbuta-1,2,3-triene (4i)

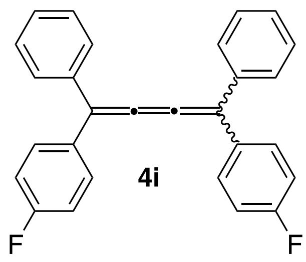

Molecular formula: $C_{28}H_{18}F_2$

Molecular Mass: 392.1377 g/mol

^{1}H NMR (600 MHz, $CDCl_3$), δ (ppm): 7.54-7.50 (m, 8H), 7.41-7.38 (m, 4H), 7.36-7.33 (m, 2H), 7.10-7.06 (m, 4H).

^{13}C{^{1}H} NMR (150 MHz, $CDCl_3$), δ (ppm): (Mixture of *E* and *Z* isomers) 163.3, 161.7, 151.3, 138.5, 138.5, 134.7, 131.0, 131.0, 130.9, 130.9, 129.2, 129.2, 128.5, 128.4, 128.1, 128.1, 121.4, 115.5, 115.5, 115.3, 115.3.

^{19}F NMR (564 MHz, $CDCl_3$), δ (ppm): −113.21, −113.27

The spectral data for this compound match that reported in the literature.[3]

IR (ATR) ν (cm^{-1}) = 3864, 3397, 3059, 2919, 2702, 2472, 2184, 1986, 1896, 1598, 1503, 1445,1231, 1156, 1014, 836.

MS (EI$^+$, 70 eV) *m/z* (%): 394.0 (4), 392.9 $[M]^+$(24), 391.8 (100), 293.8 (9), 195.8 (66).

HRMS (ESI$^+$) m/z: calcd. for $[M]^+$ = $[C_{28}H_{18}F_2]^+$: 392.4488; found: 392.4488.

1,1,6,6-Tetraphenylhexa-2,4-diyne-1,6-diol (5a)

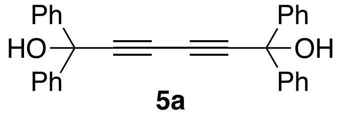

5a

Molecular formula: $C_{30}H_{22}O_2$

Molecular Mass: 414.1620 g/mol

^{1}H NMR (600 MHz, $CDCl_3$), δ (ppm): 7.57 (d, *J* = 7.58 Hz, 8H), 7.35 (t, *J* = 7.96 Hz, 8H), 7.29 (t, *J* = 7.89 Hz, 4H), 2.87 (s, 2H).

^{13}C{^{1}H} NMR (150 MHz, $CDCl_3$), δ (ppm): 143.9, 128.5, 128.2, 126.2, 82.8, 75.1, 71.3. The spectral data for this compound match that reported in the literature.[10]

IR (ATR) ν (cm^{-1}) = 3524, 3063, 3027, 2323, 2184, 2096, 1913, 1596, 1488, 1449, 1339, 1039, 692.

MS (EI$^+$, 70 eV) *m/z* (%): 396.3 (36) $[M-H_2O]^+$, 309.1 (56), 291.2 (31), 202.1 (32), 178.1 (11), 165.1 (14), 105.2 (100), 77.3 (50).

HRMS (ESI$^+$) m/z: calcd. for $[M+Na]^+$ = $[C_{30}H_{22}O_2Na]^+$: 437.1512; found: 437.1511.

1,1,6,6-Tetraphenyl-1,2,3,4,5-hexapentaene (6a)

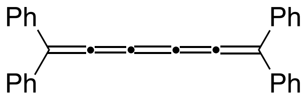

6a

Molecular formula: $C_{30}H_{20}$

Molecular Mass: 380.1565 g/mol

^{1}H NMR (400 MHz, $CDCl_3$), δ (ppm): 7.60-7.57 (m, 8H), 7.43-7.35 (m, 12H).
^{13}C{^{1}H} NMR (100 MHz, $CDCl_3$), δ (ppm): 149.5, 138.2, 129.5, 128.7, 128.7, 127.4, 124.9. The spectral data for this compound match that reported in the literature.[10]

IR (ATR) ν (cm^{-1}) = 3439, 3053, 2922, 2853, 2328, 2100, 1996, 1898, 1661, 1590, 1485, 1442, 1275, 1176, 1072, 688.

MS (EI^{+}, 70 eV) *m/z* (%): 381.3 (35), 380.3 $[M]^{+}$(100), 356.2 (42), 178.1 (20), 165.2 (7), 139.2(1).
HRMS (ESI^{+}) m/z: calcd. for $[M]^{+}$ = $[C_{30}H_{20}]^{+}$: 380.1559; found: 380.1558.

8. NMR spectra

^{1}H NMR of 2a

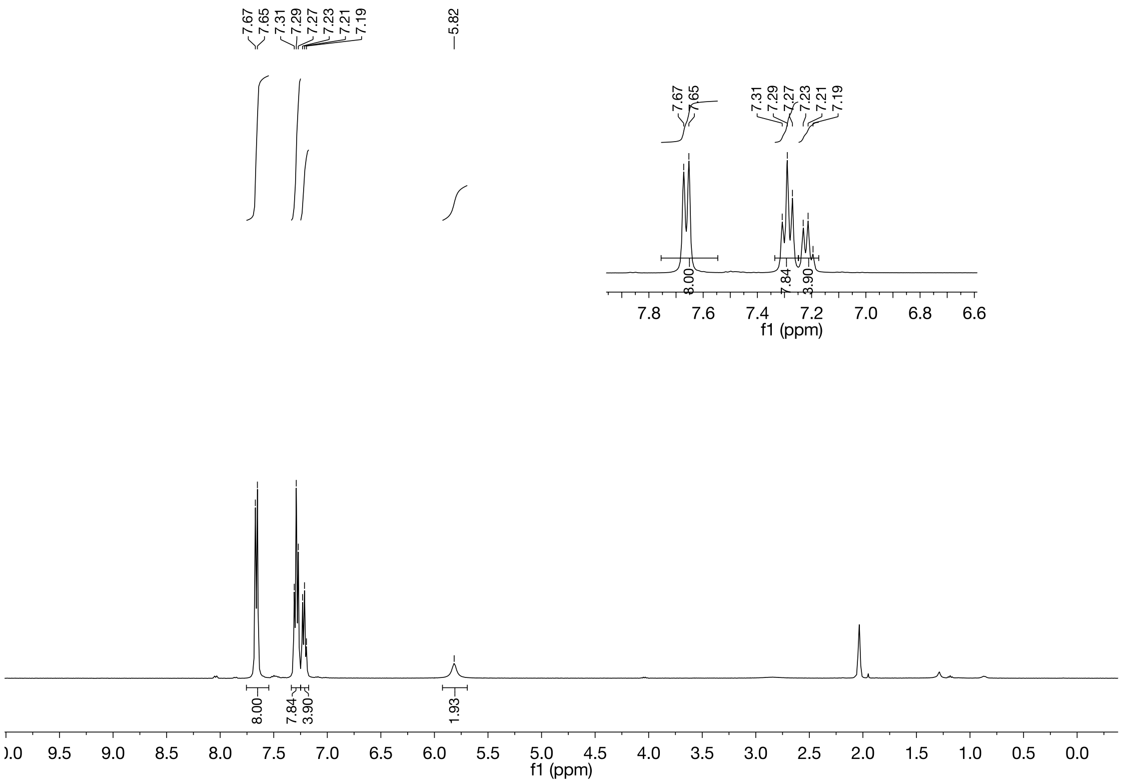

^{13}C NMR of 2a

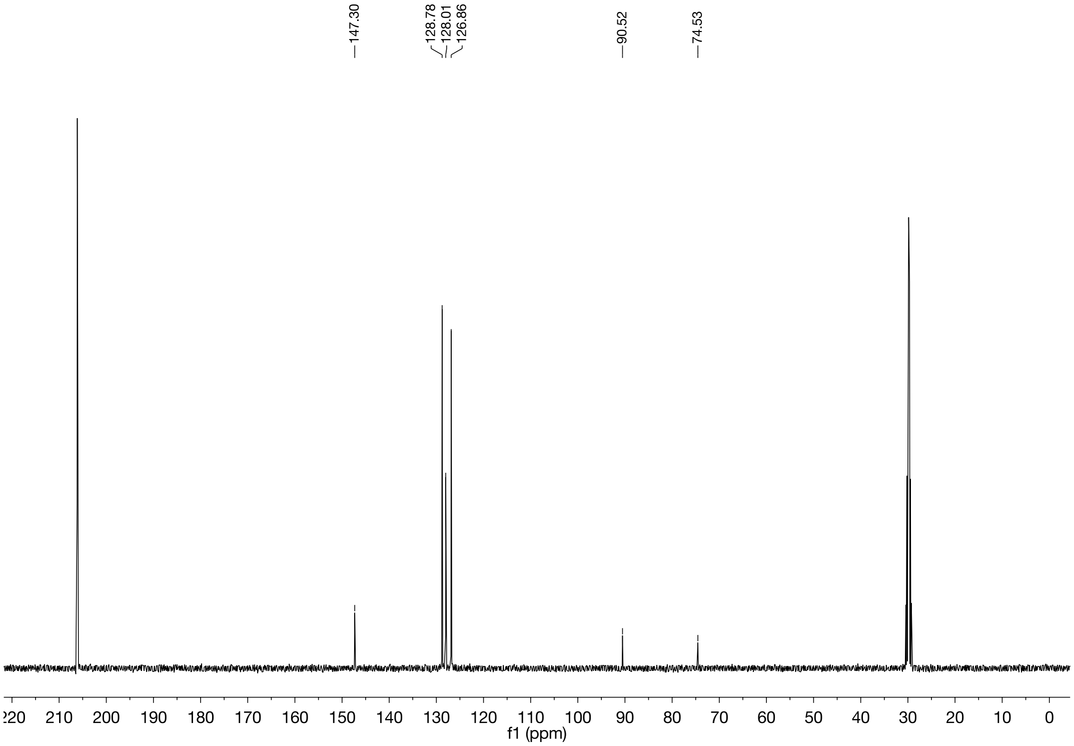

^{1}H NMR of 3a

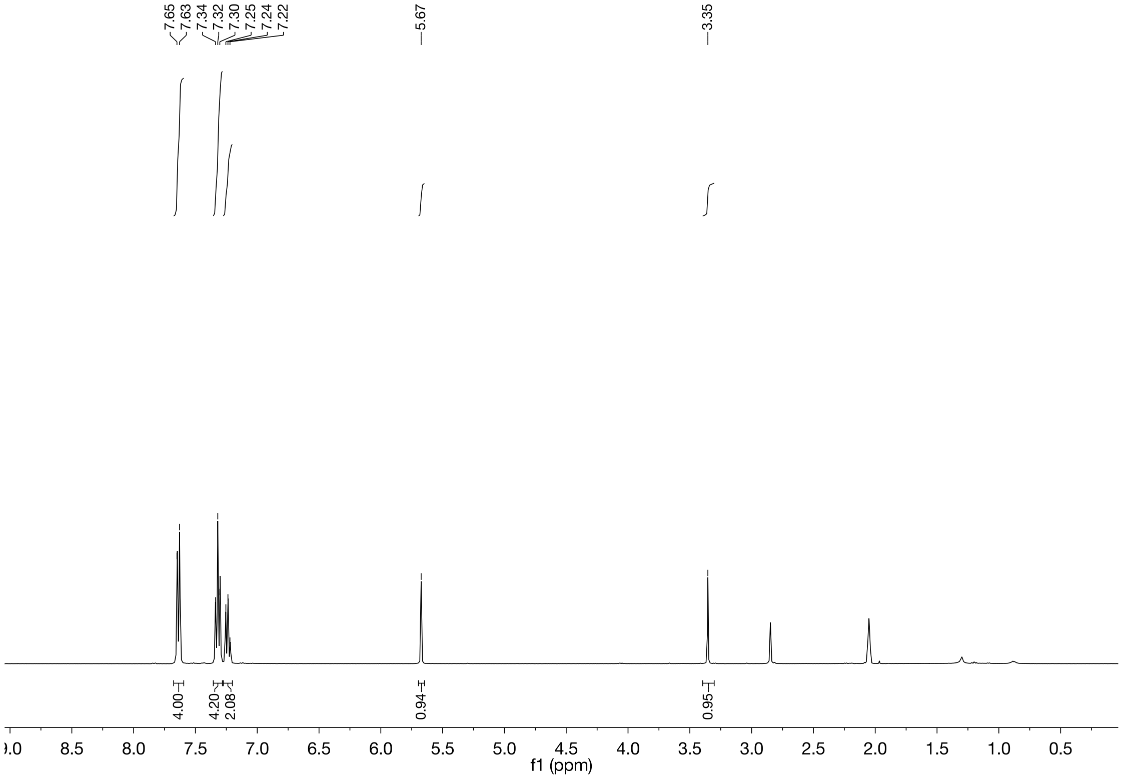

^{13}C NMR of 3a

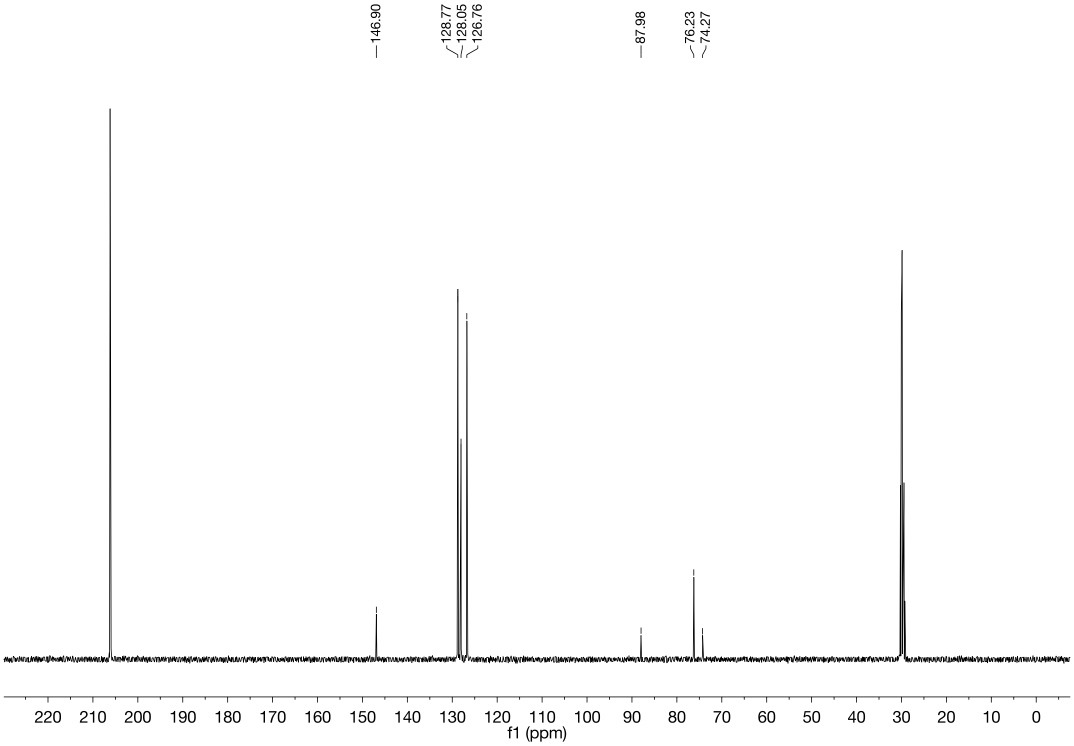

^{1}H NMR of 2b

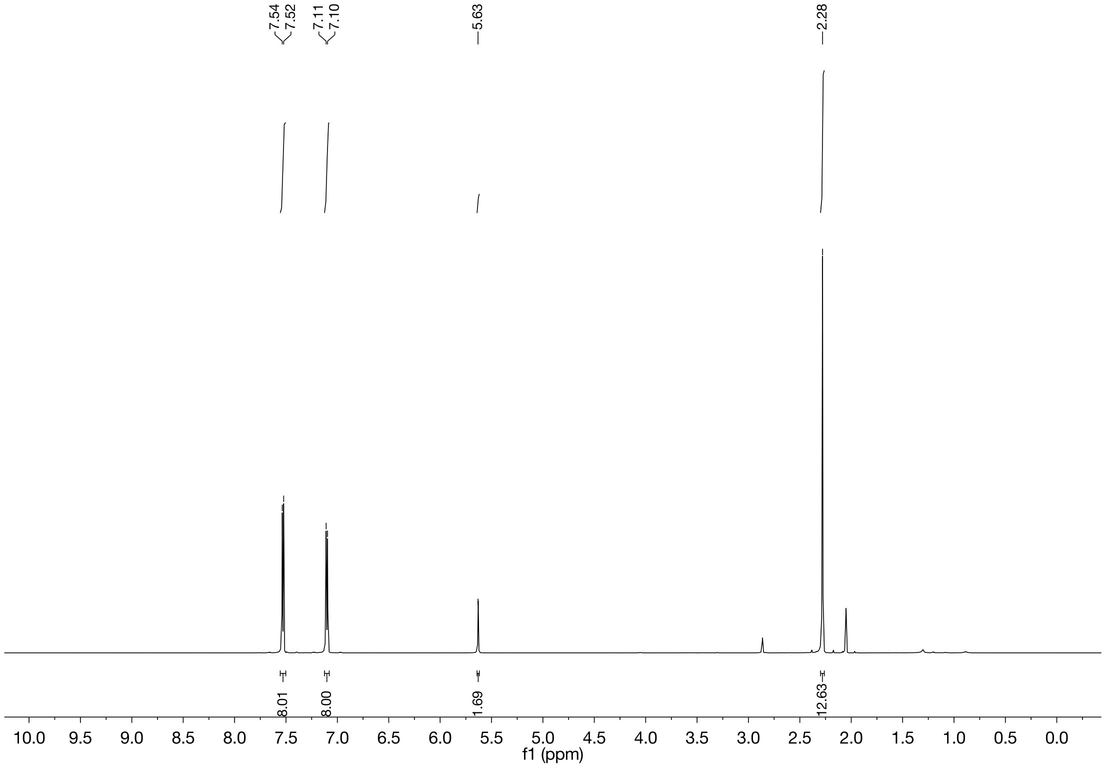

^{13}C NMR of 2b

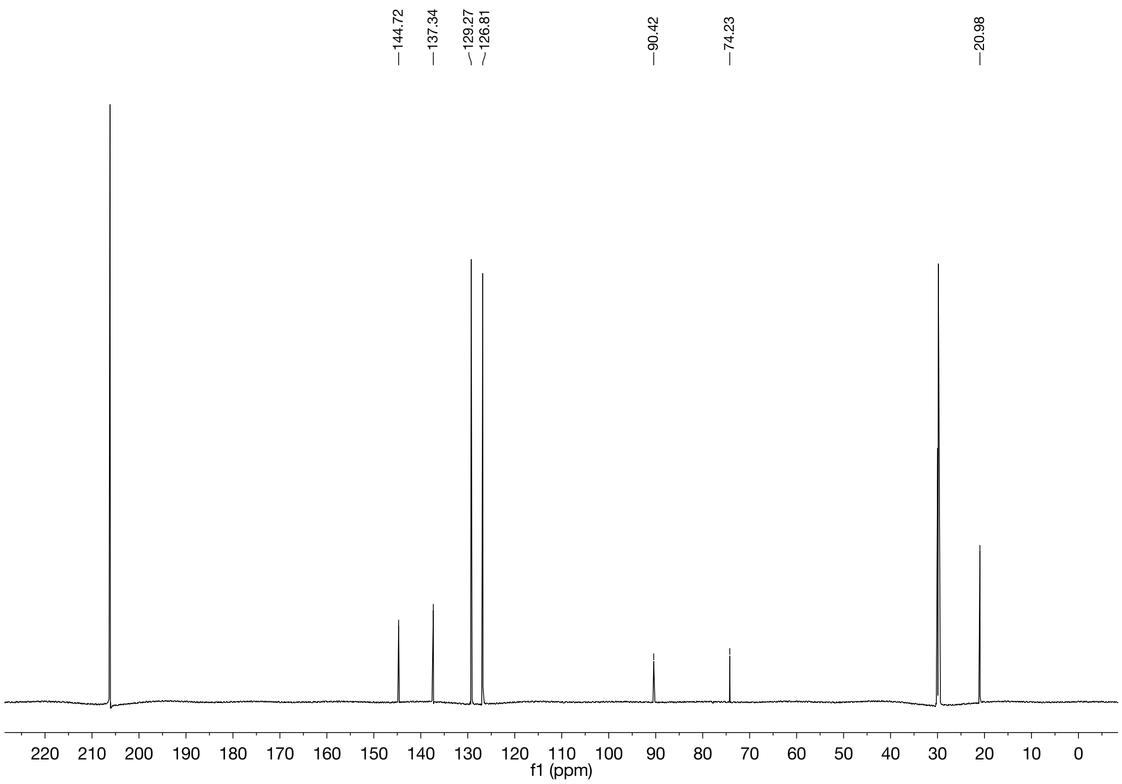

^{1}H NMR of 3b

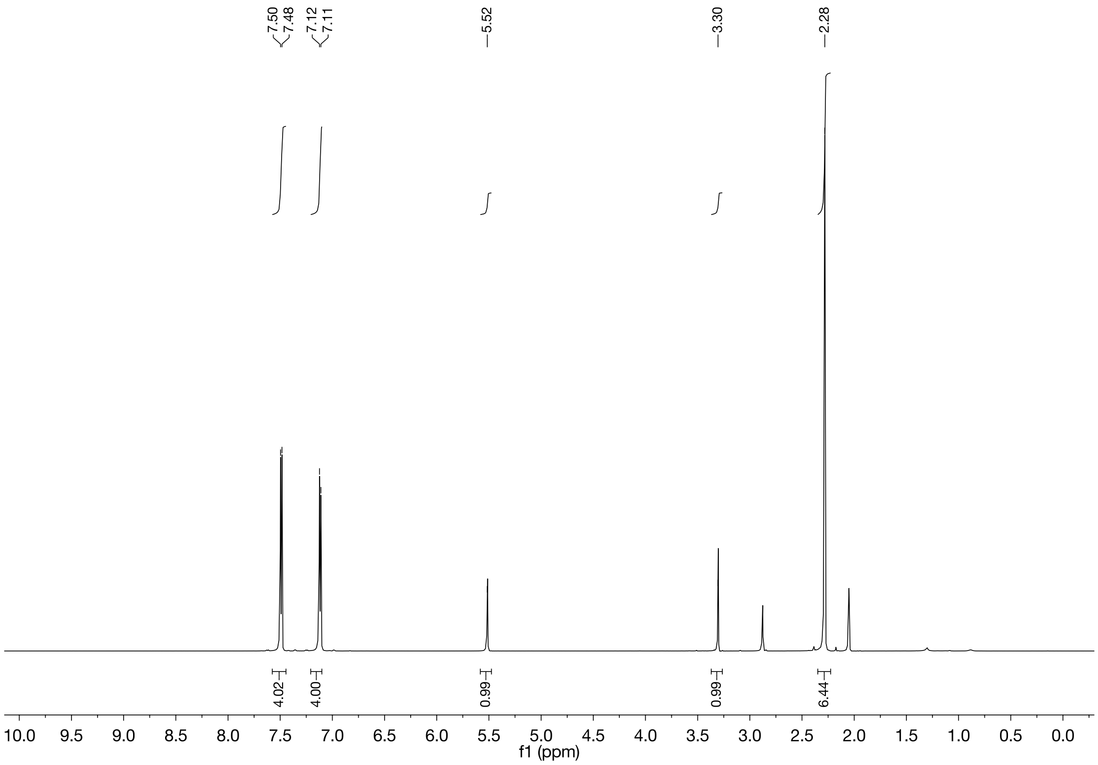

^{13}C NMR of 3b

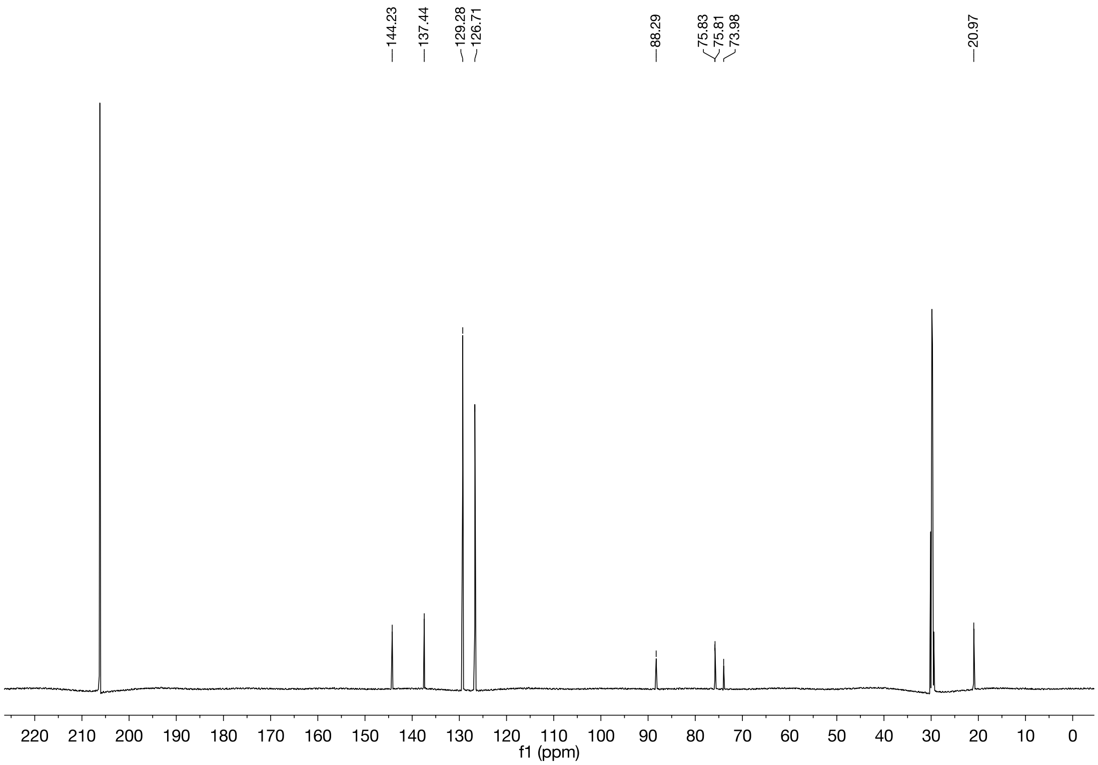

^{1}H NMR of 2c

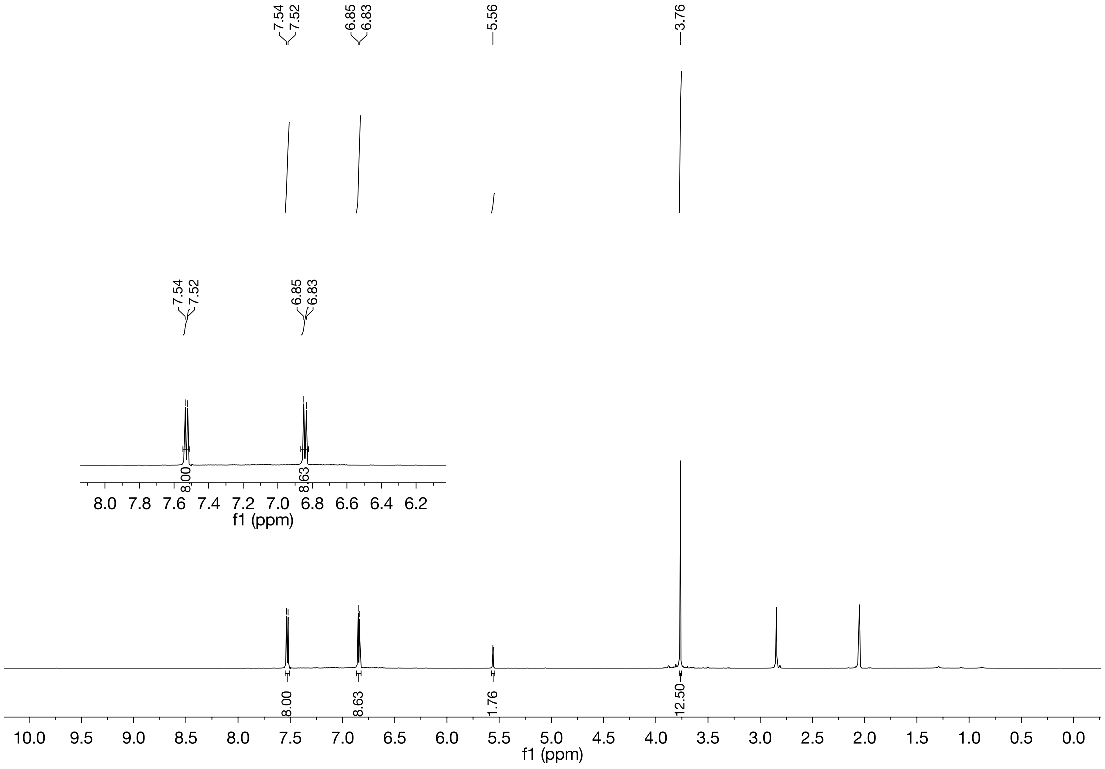

^{13}C NMR of 2c

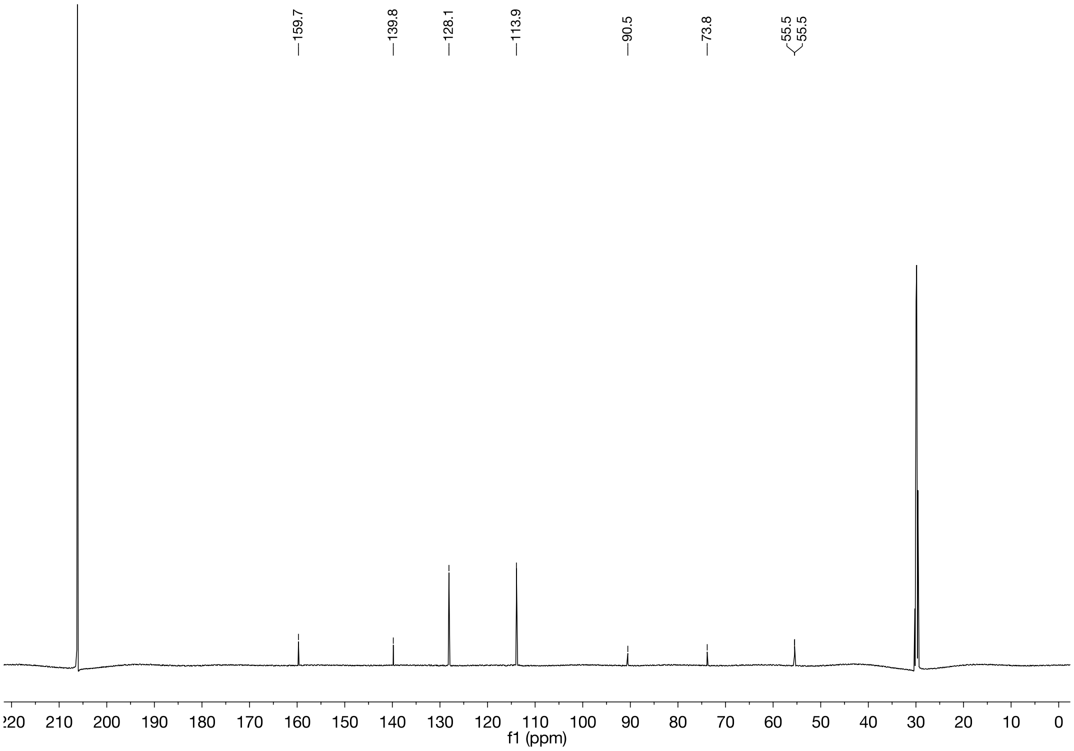

^{1}H NMR of 3c

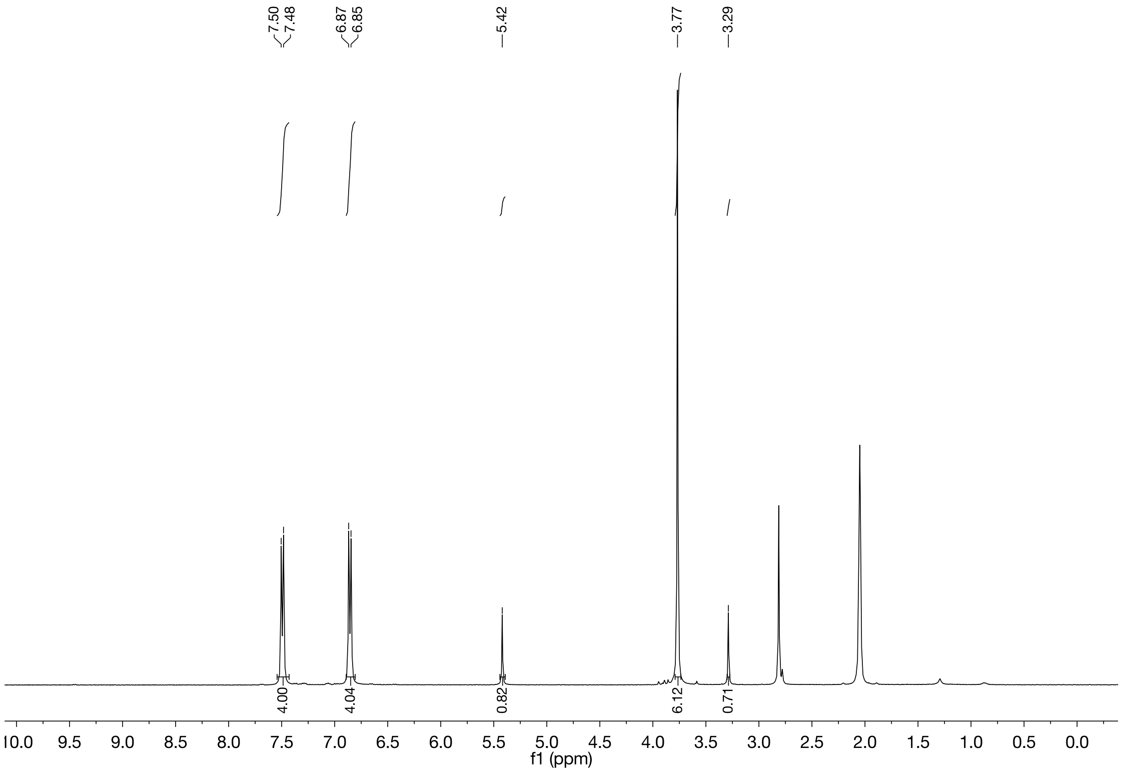

^{13}C NMR of 3c

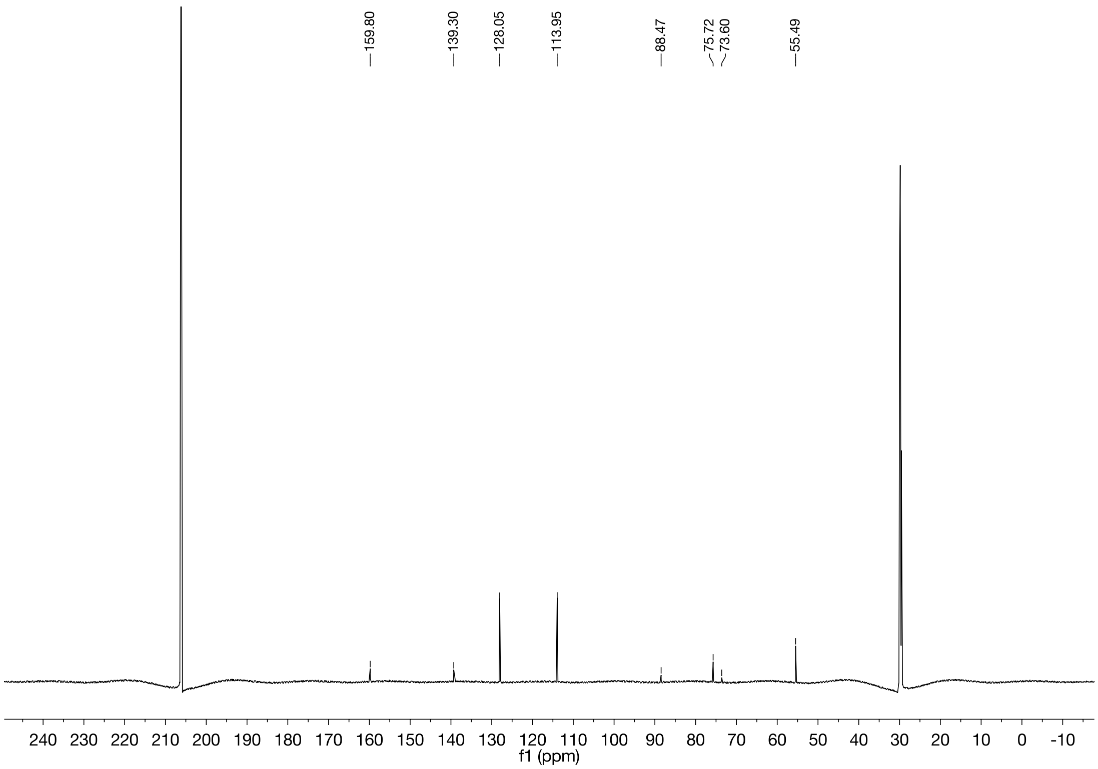

^{1}H NMR of 2d

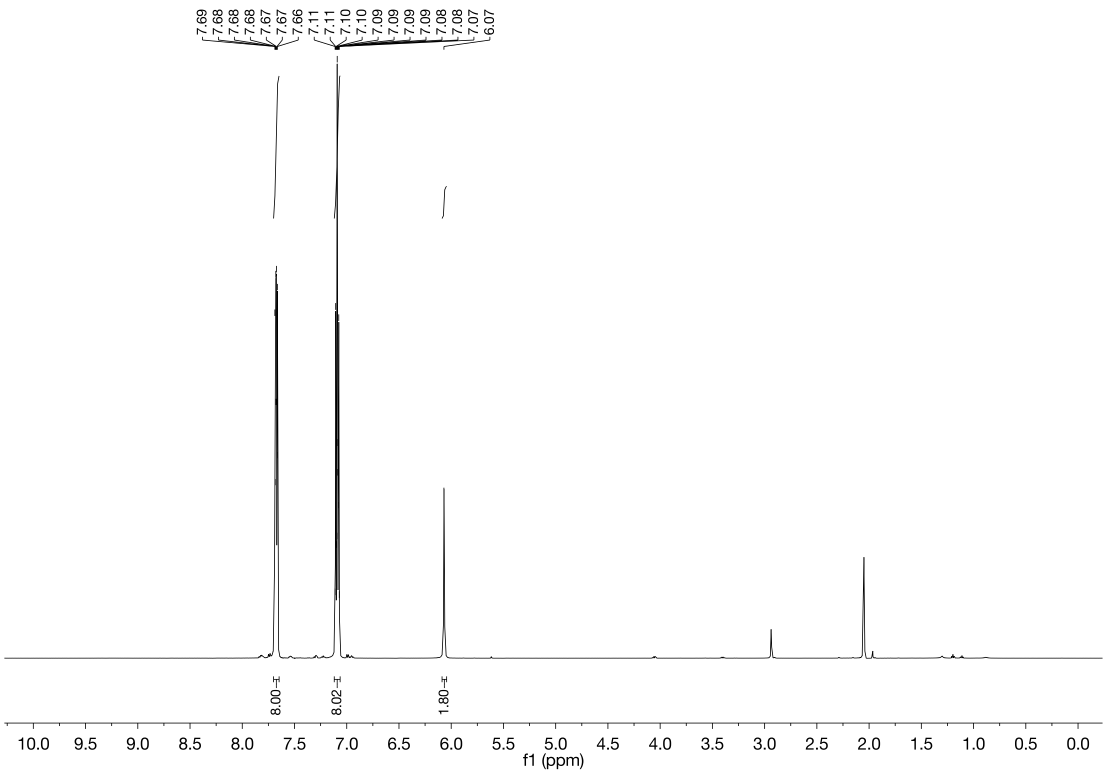

^{13}C NMR of 2d

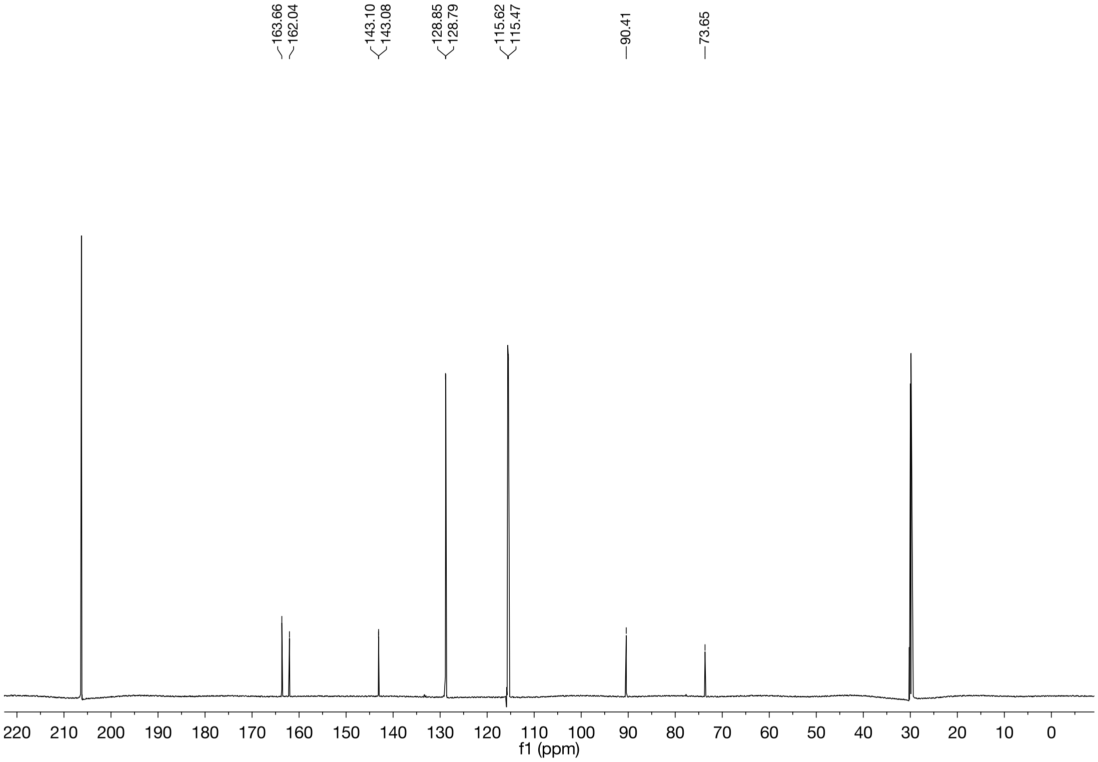

^{19}F NMR of 2d

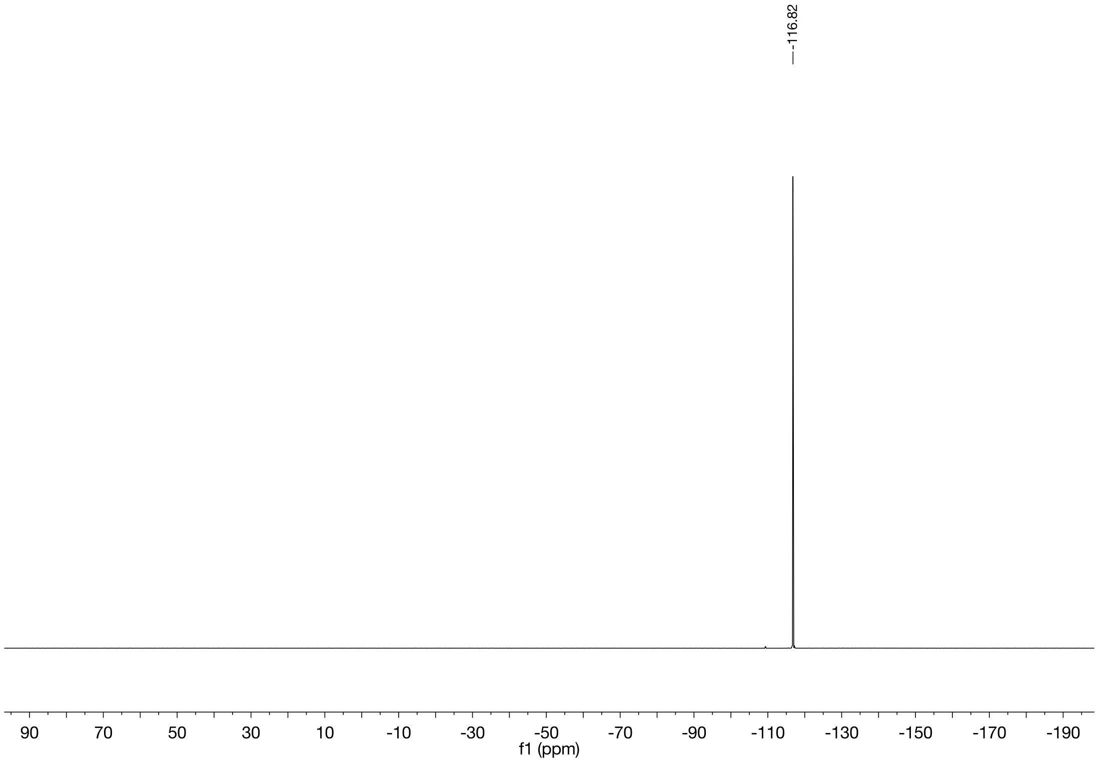

^{1}H NMR of 3d

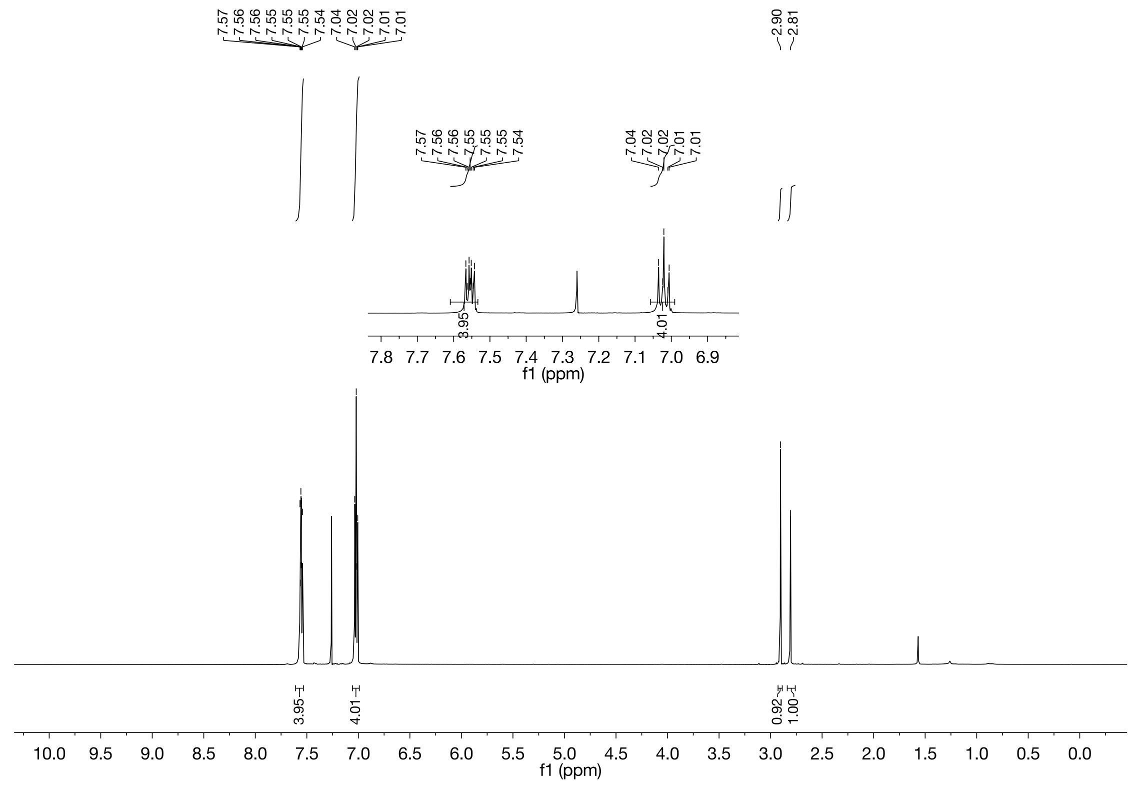

^{13}C NMR of 3d

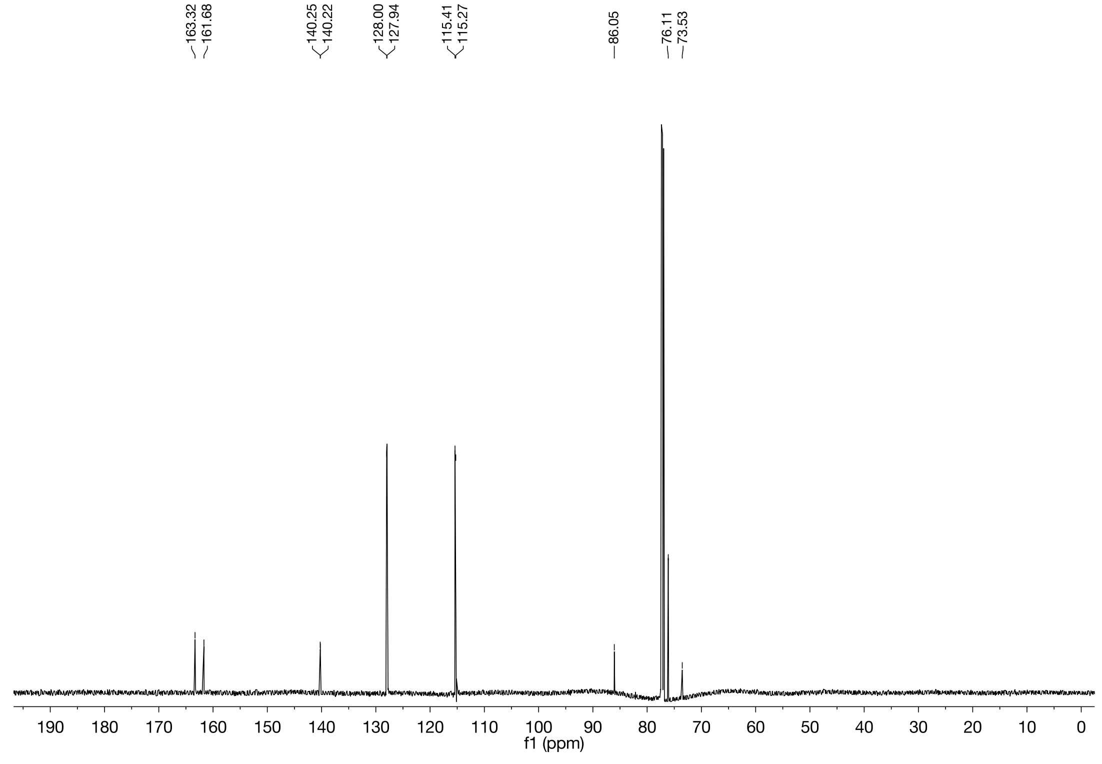

^{19}F NMR of 3d

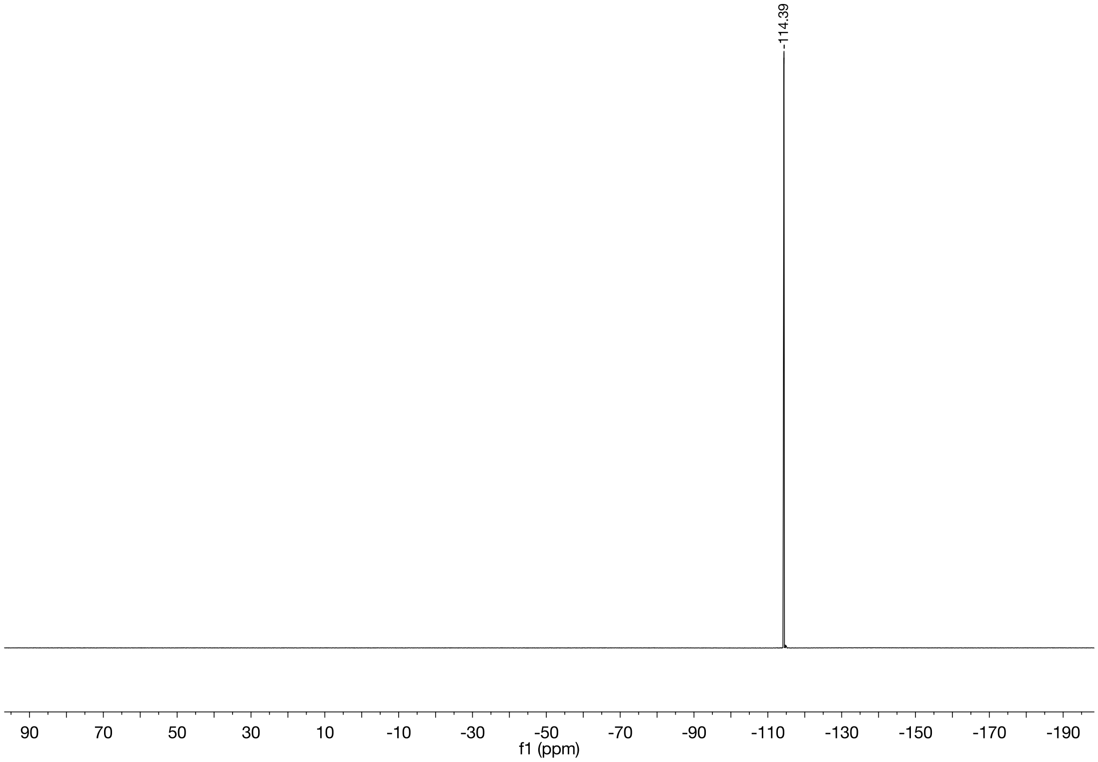

^{1}H NMR of 2e

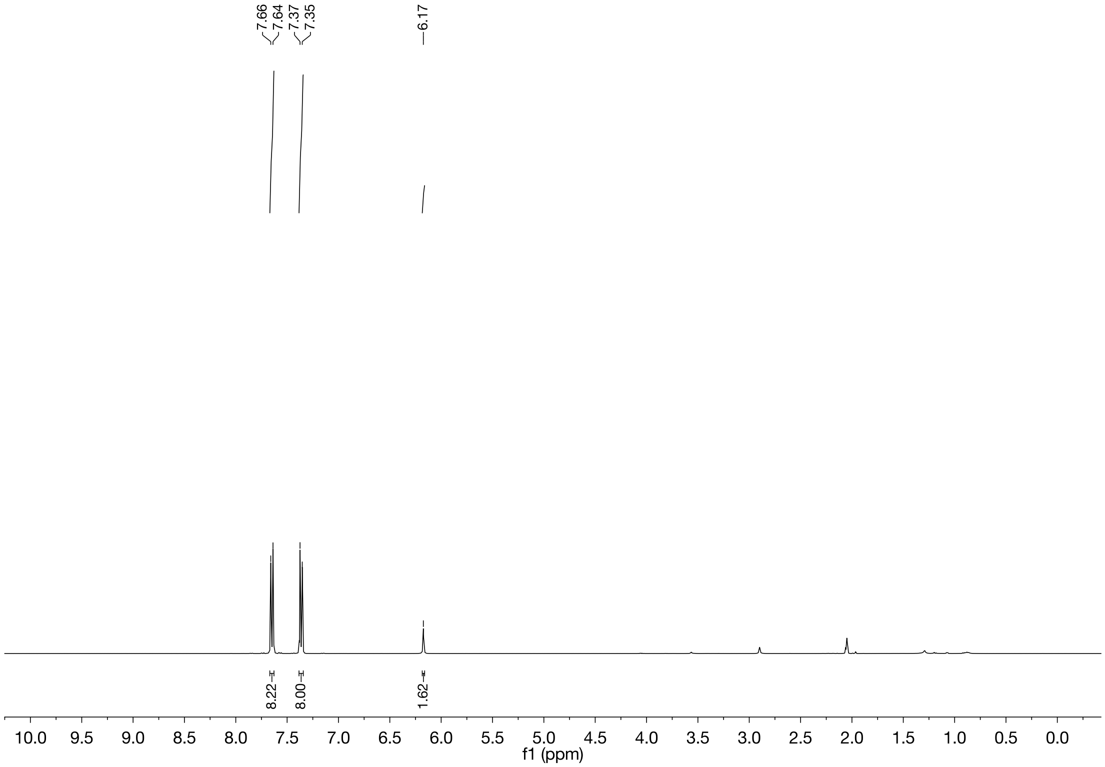

^{13}C NMR of 2e

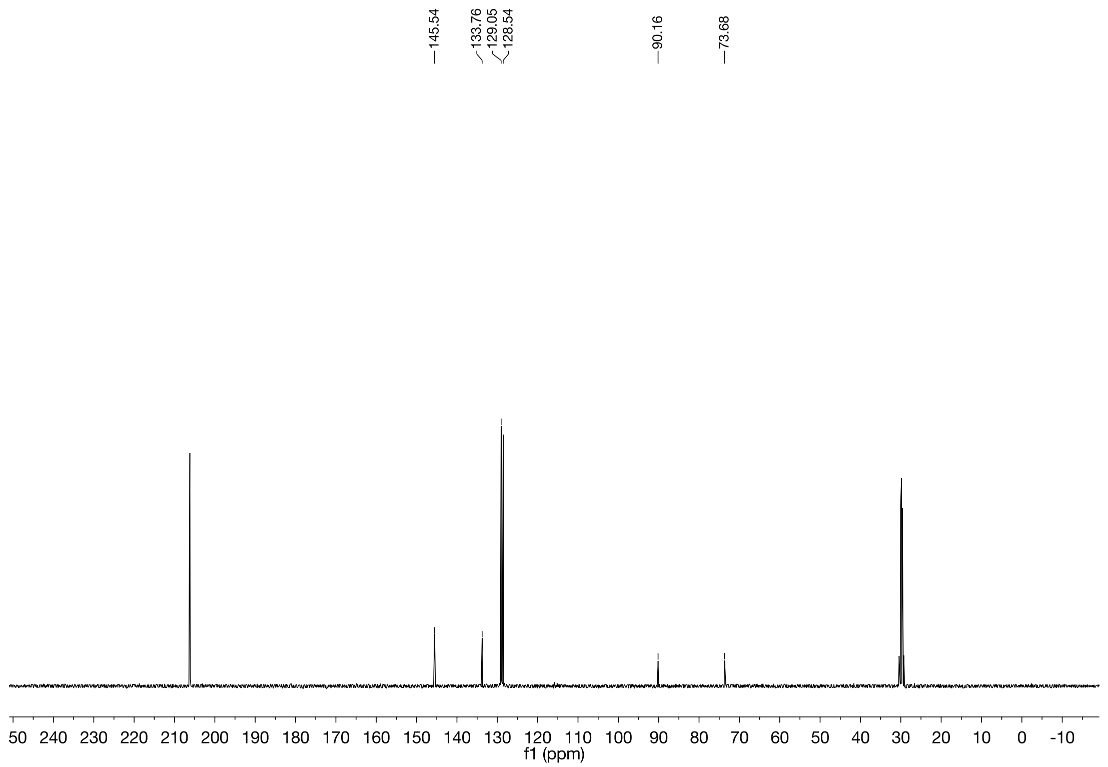

^{1}H NMR of 3e

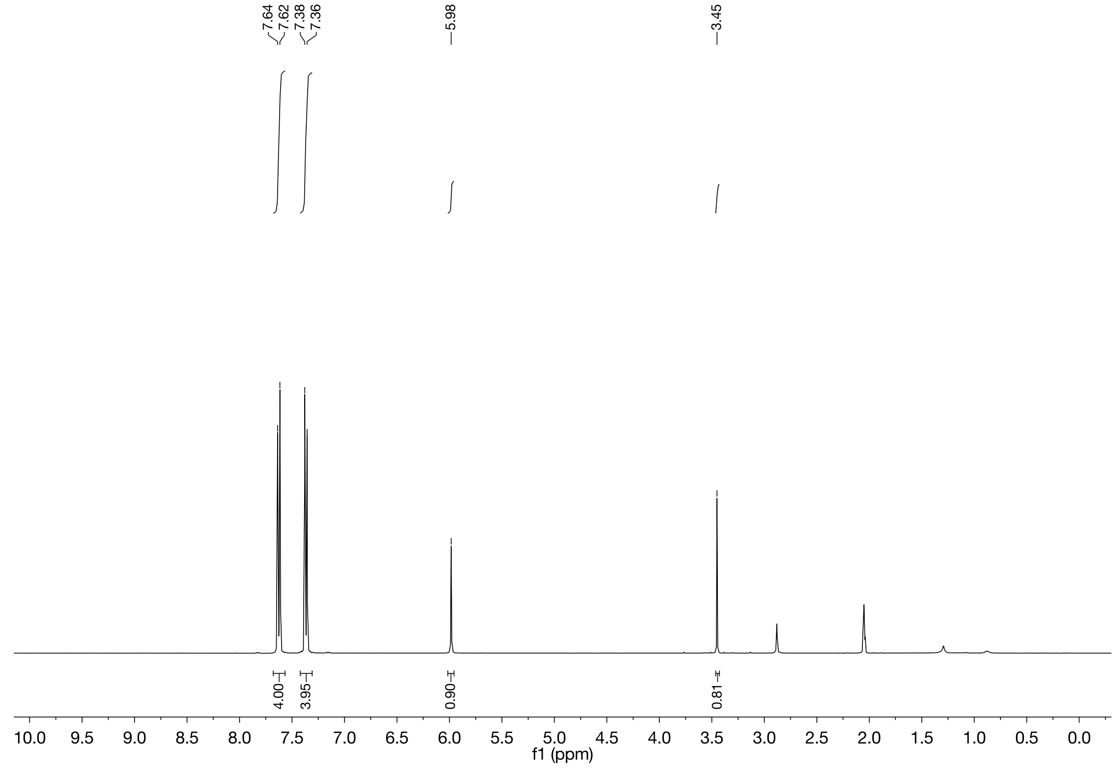

^{13}C NMR of 3e

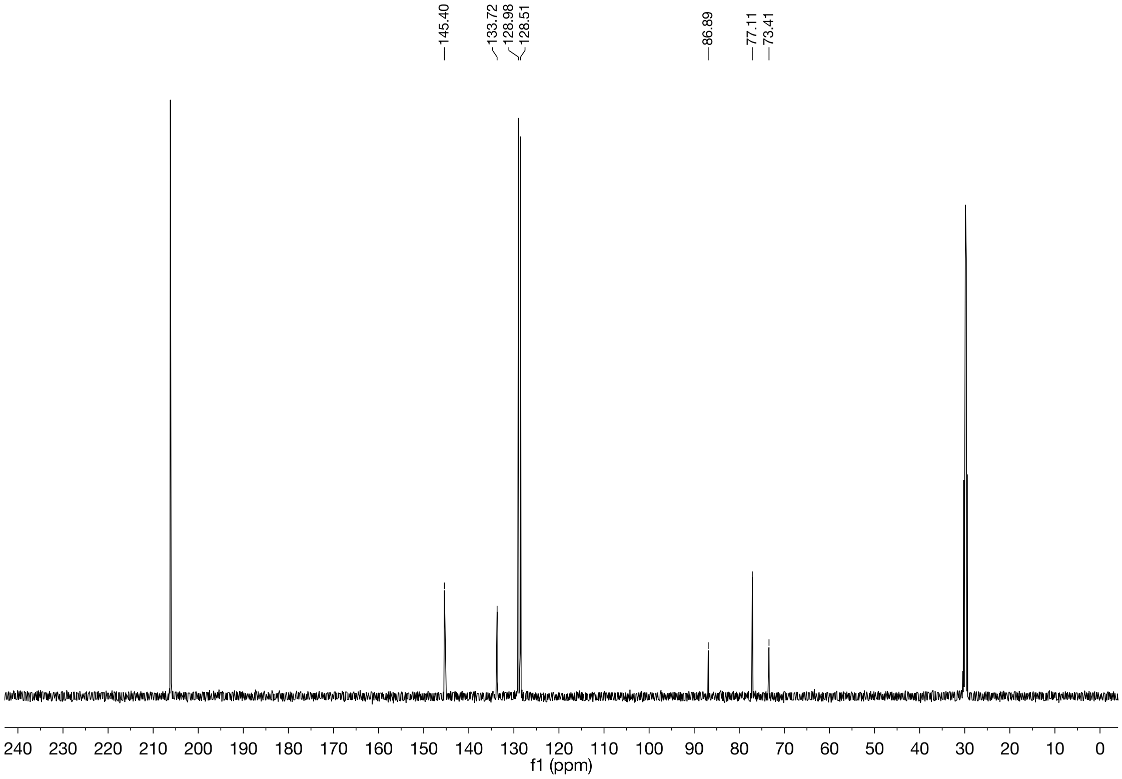

^{1}H NMR of 2f

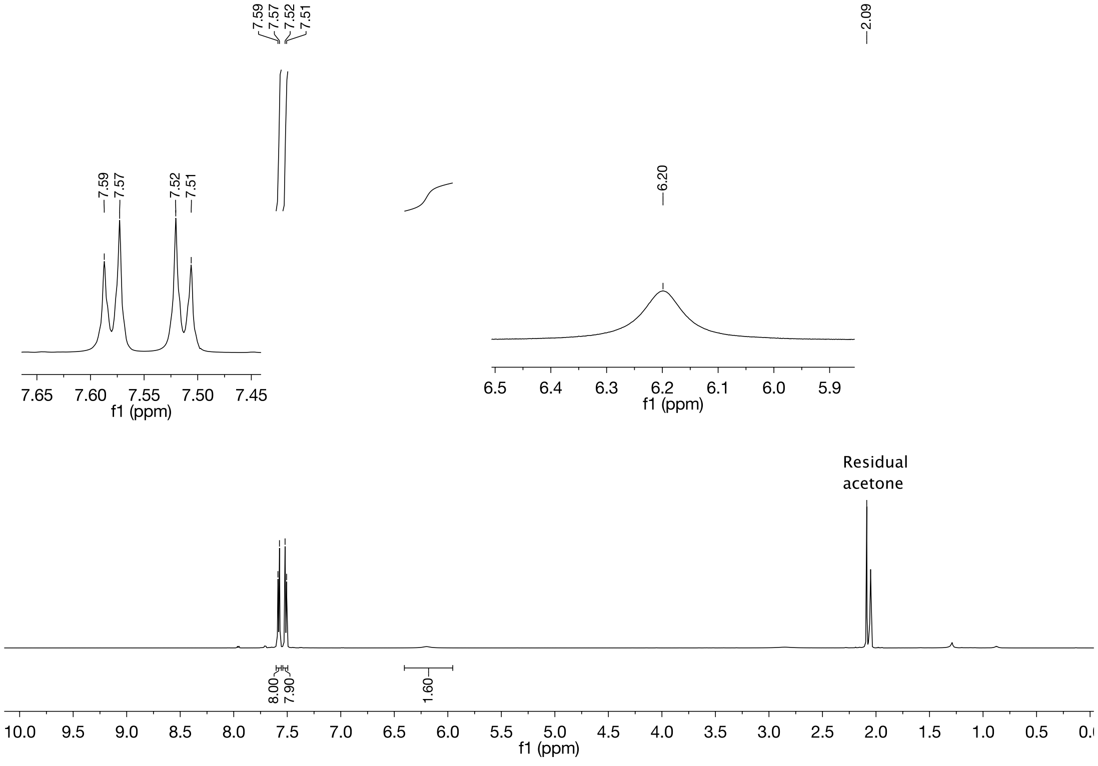

^{13}C NMR of 2f

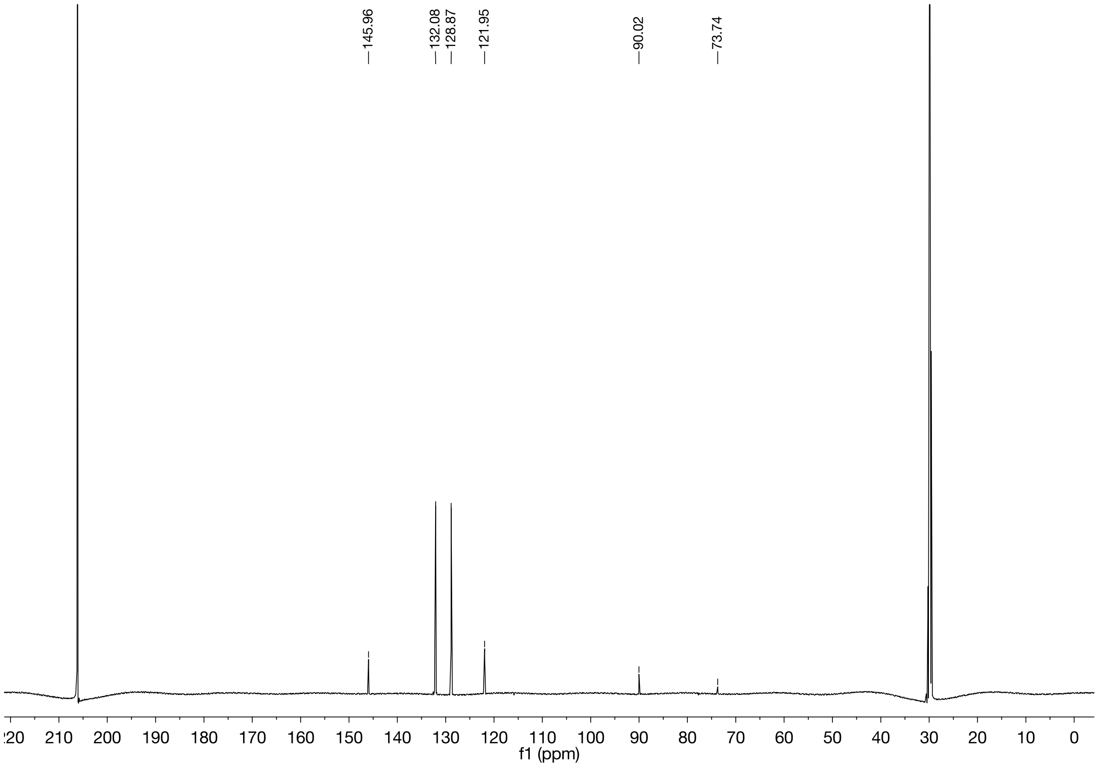

^{1}H NMR of 3f

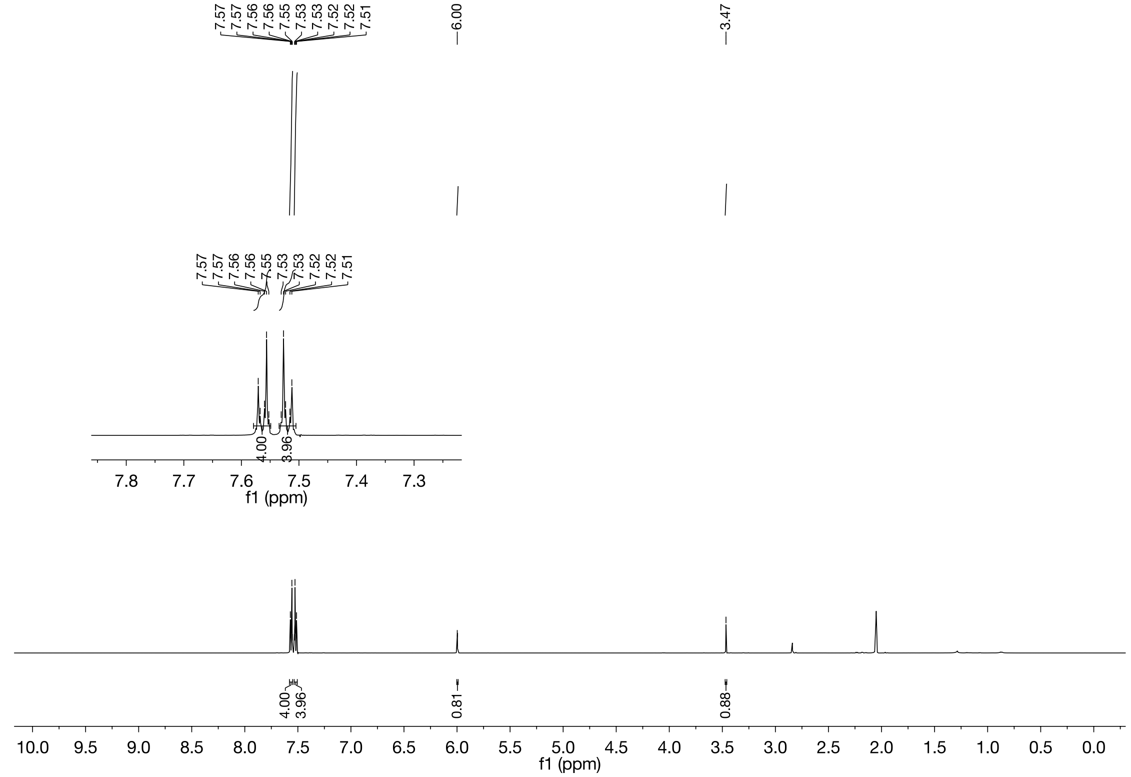

^{13}C NMR of 3f

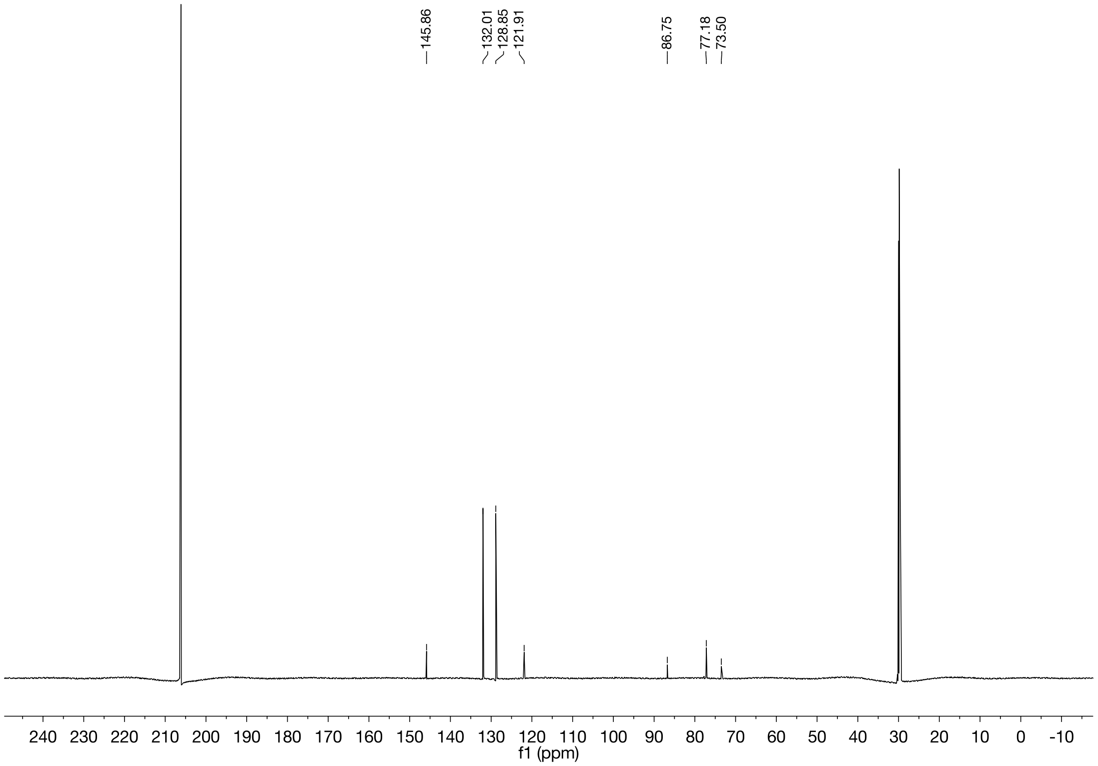

^{1}H NMR of 2g

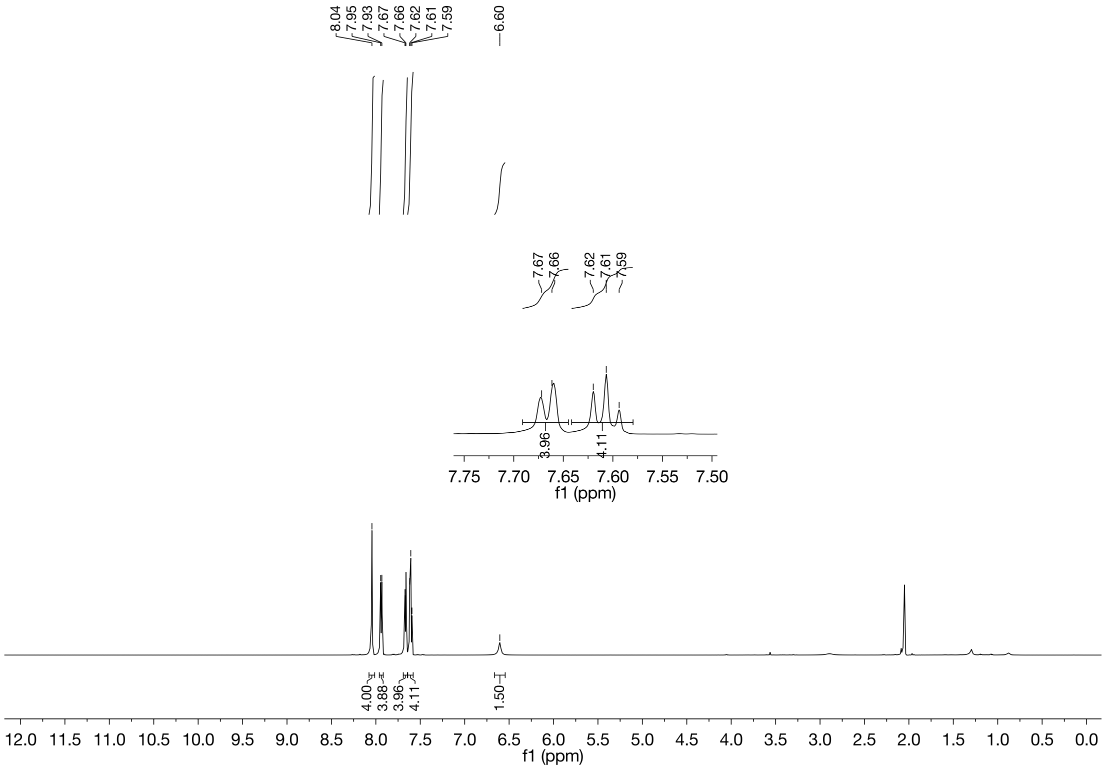

^{13}C NMR of 2g

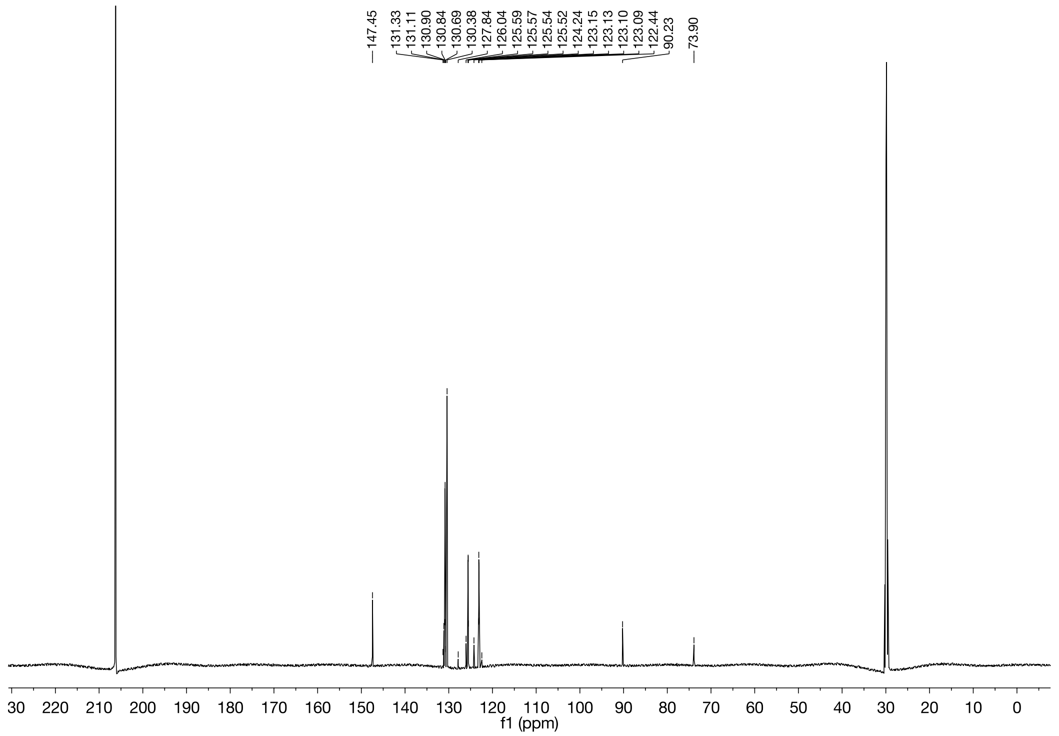

^{19}F NMR of 2g

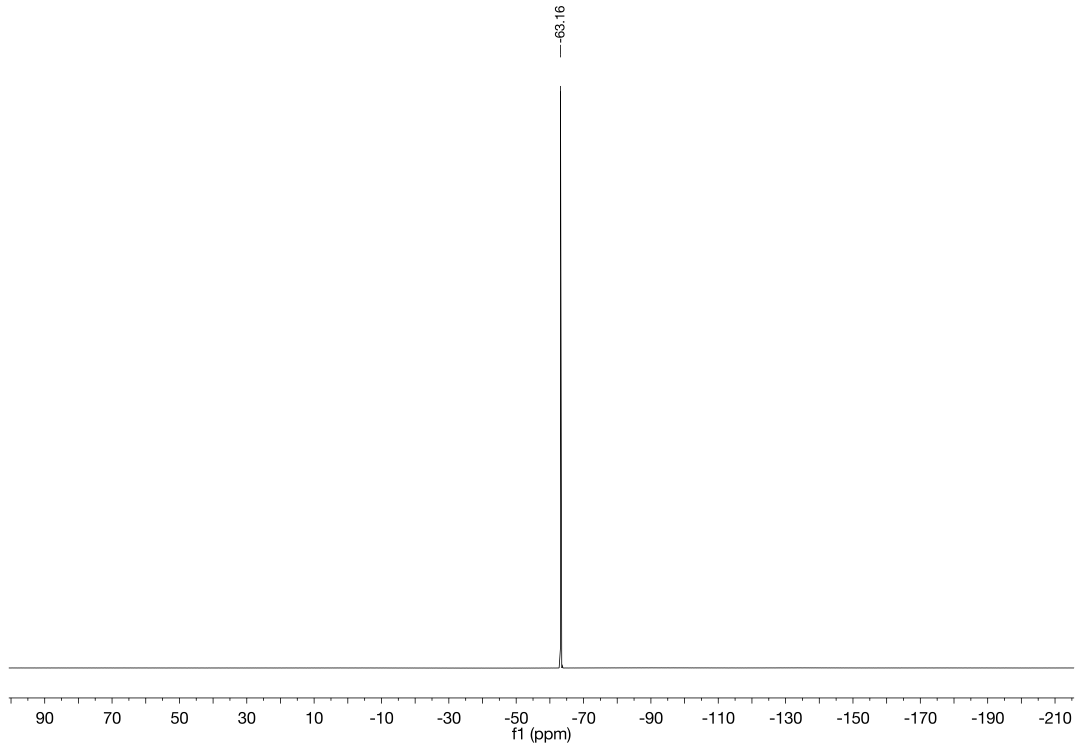

^{1}H NMR of 3g

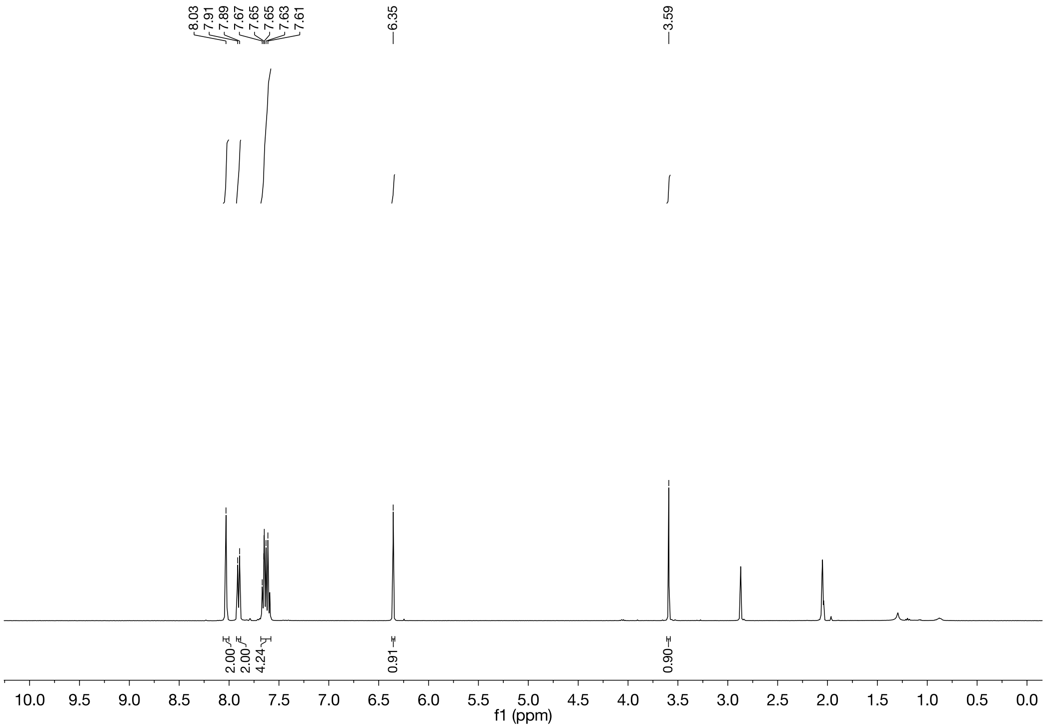

^{13}C NMR of 3g

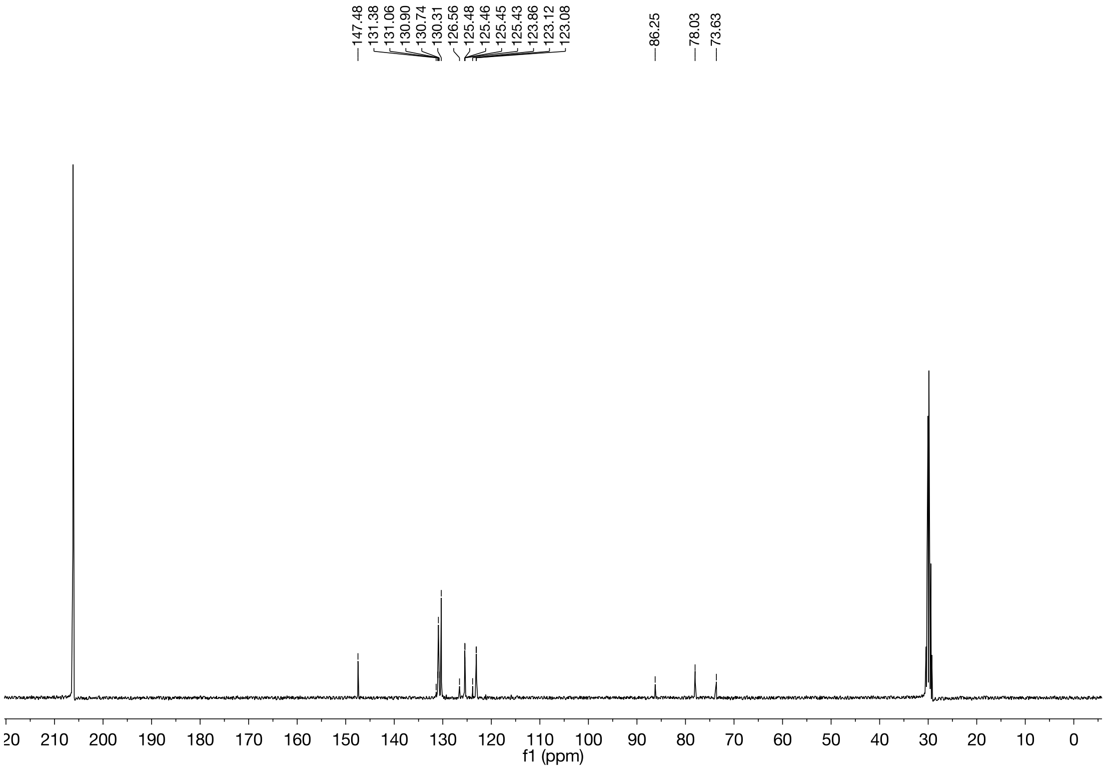

^{19}F NMR of 3g

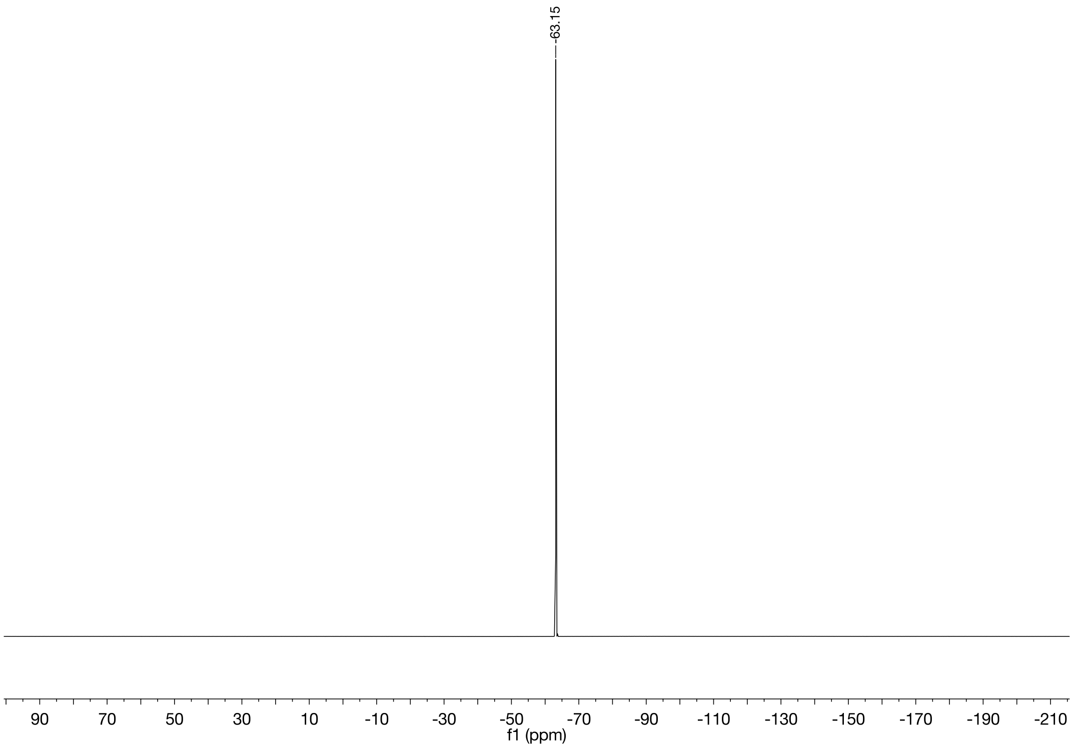

^{1}H NMR of 2h

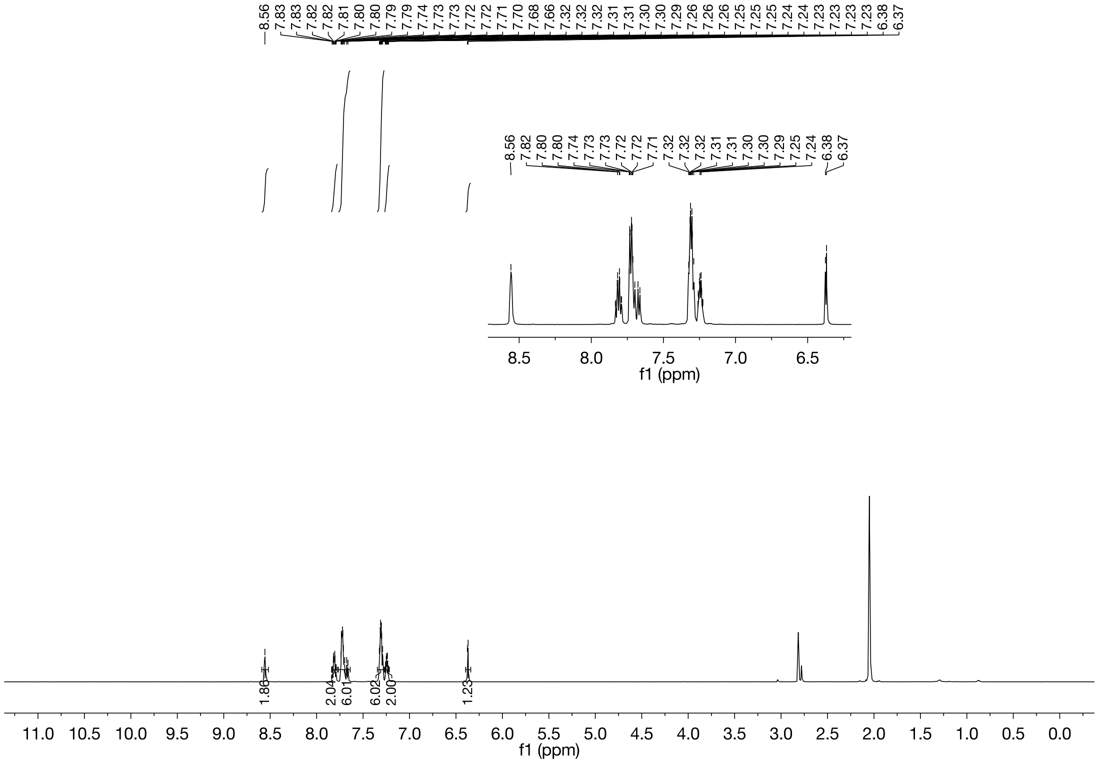

^{13}C NMR of 2h

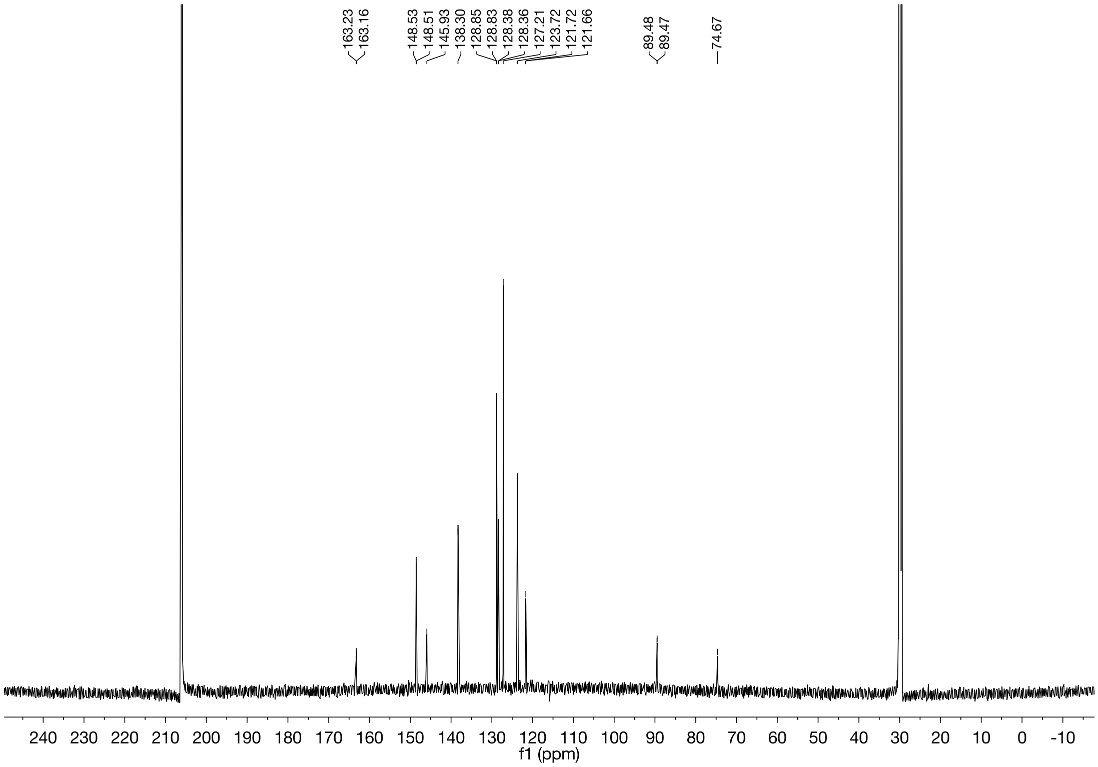

^{1}H NMR of 3h

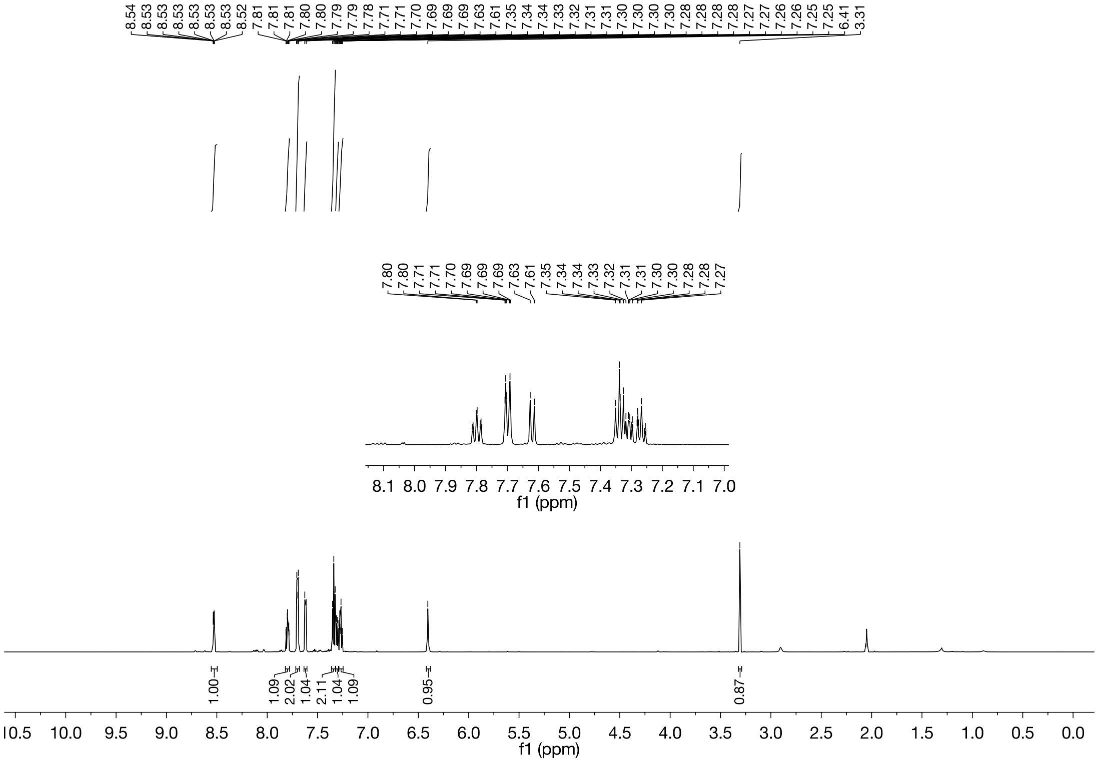

^{13}C NMR of 3h

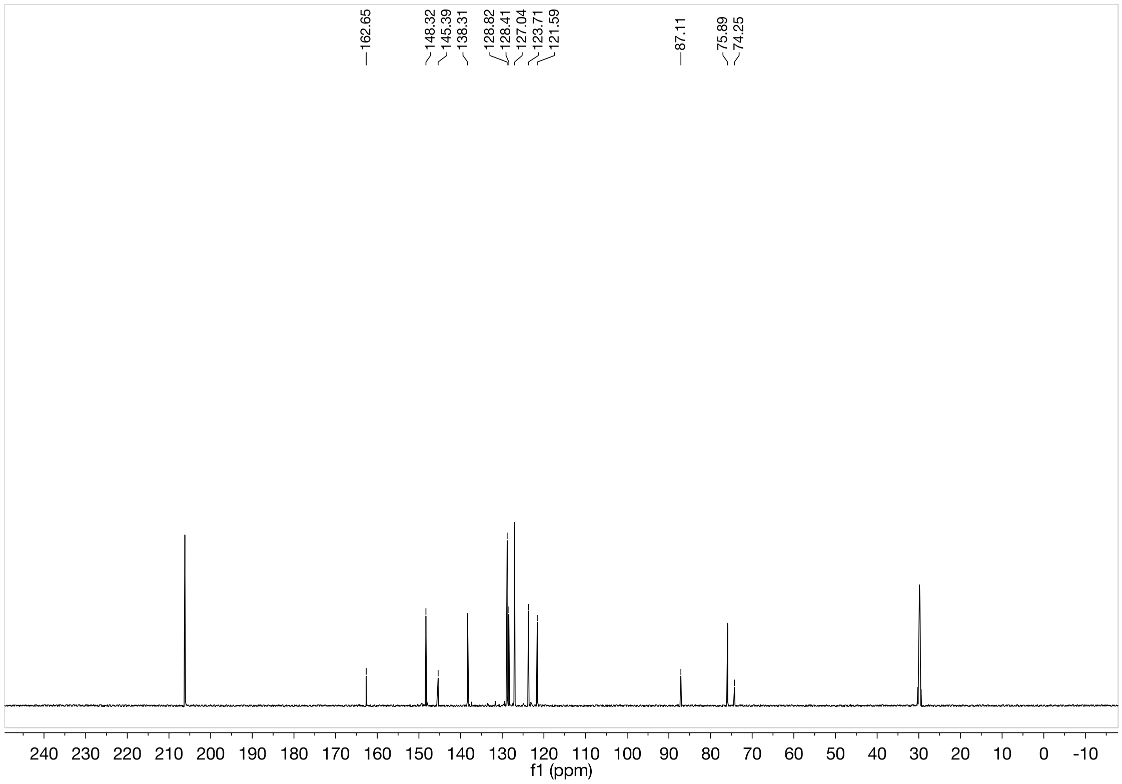

^{1}H NMR of 2i

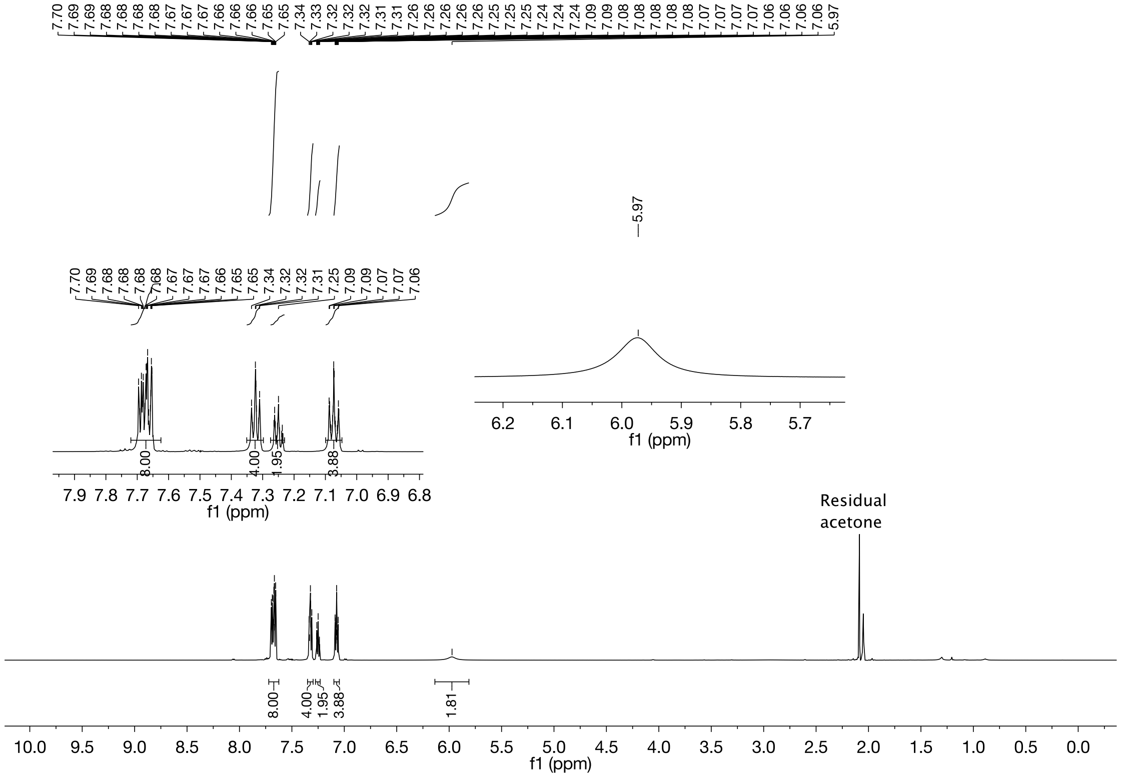

^{13}C NMR of 2i

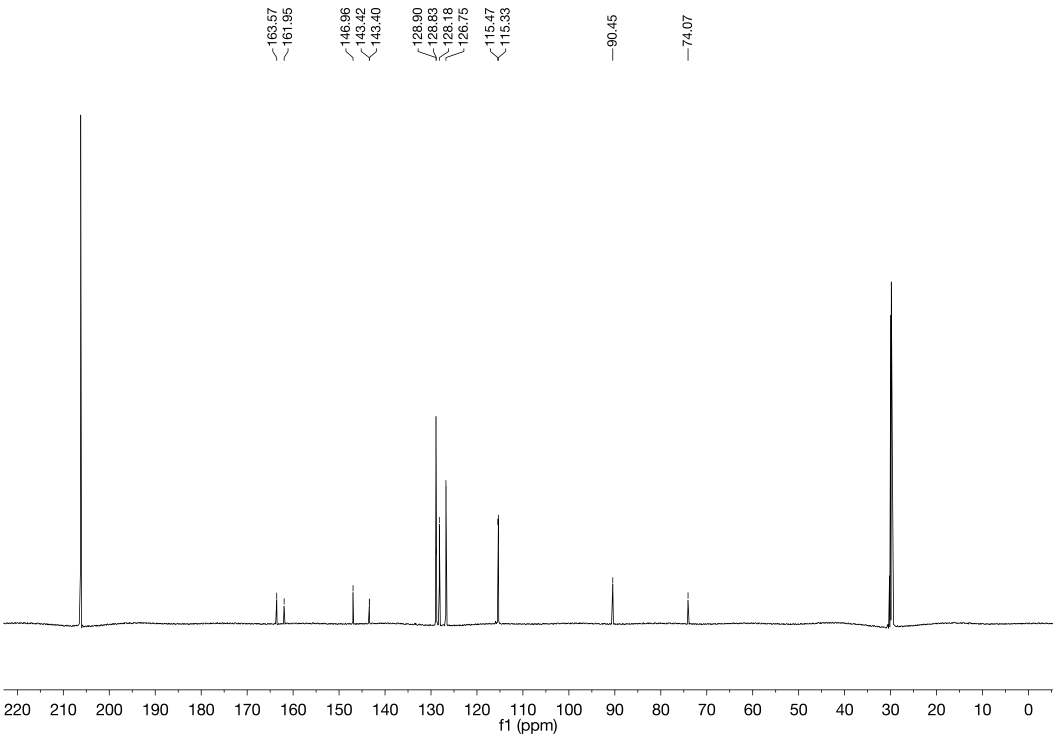

^{19}F NMR of 2i

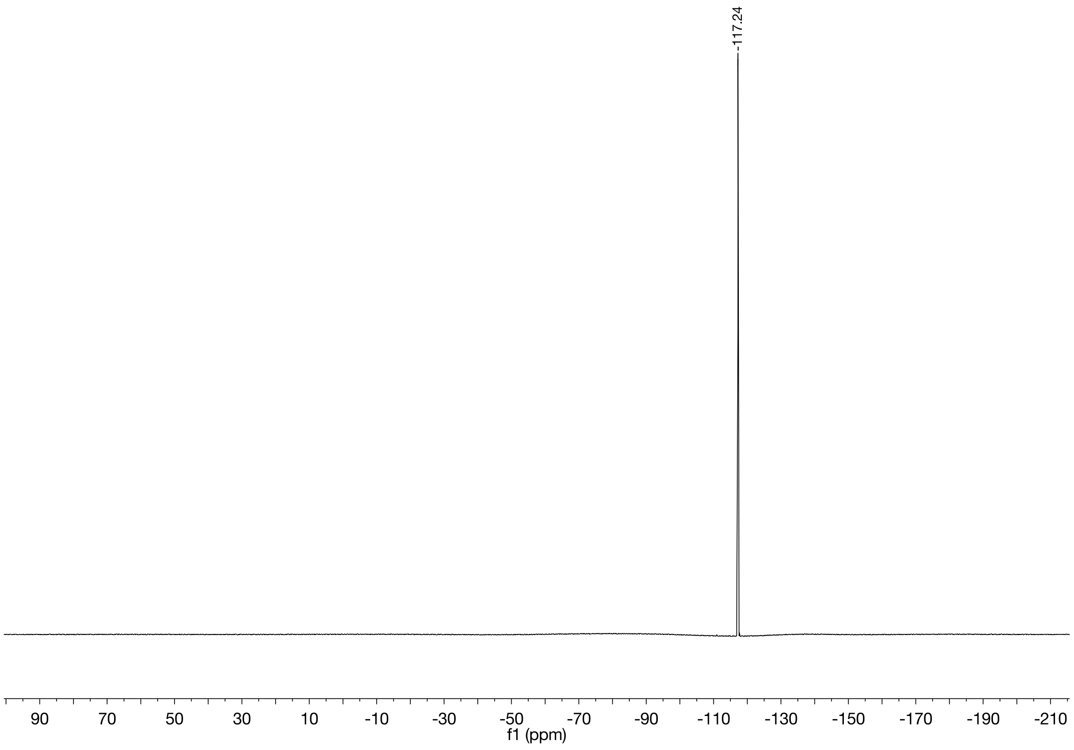

^{1}H NMR of 3i

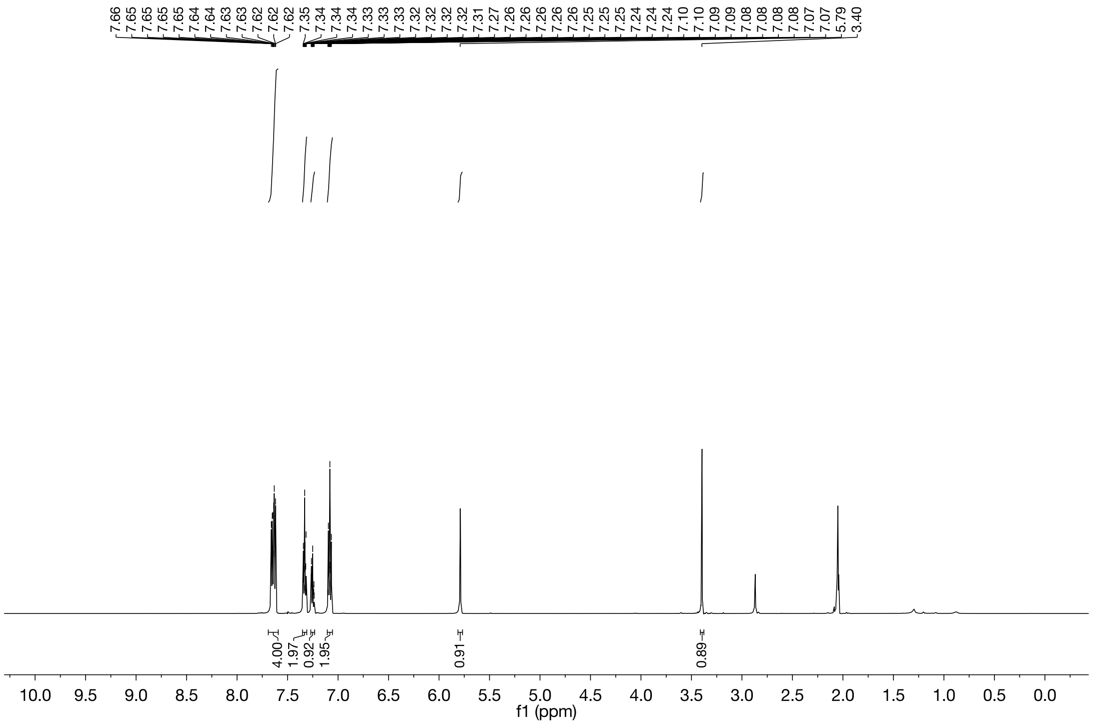

^{13}C NMR of 3i

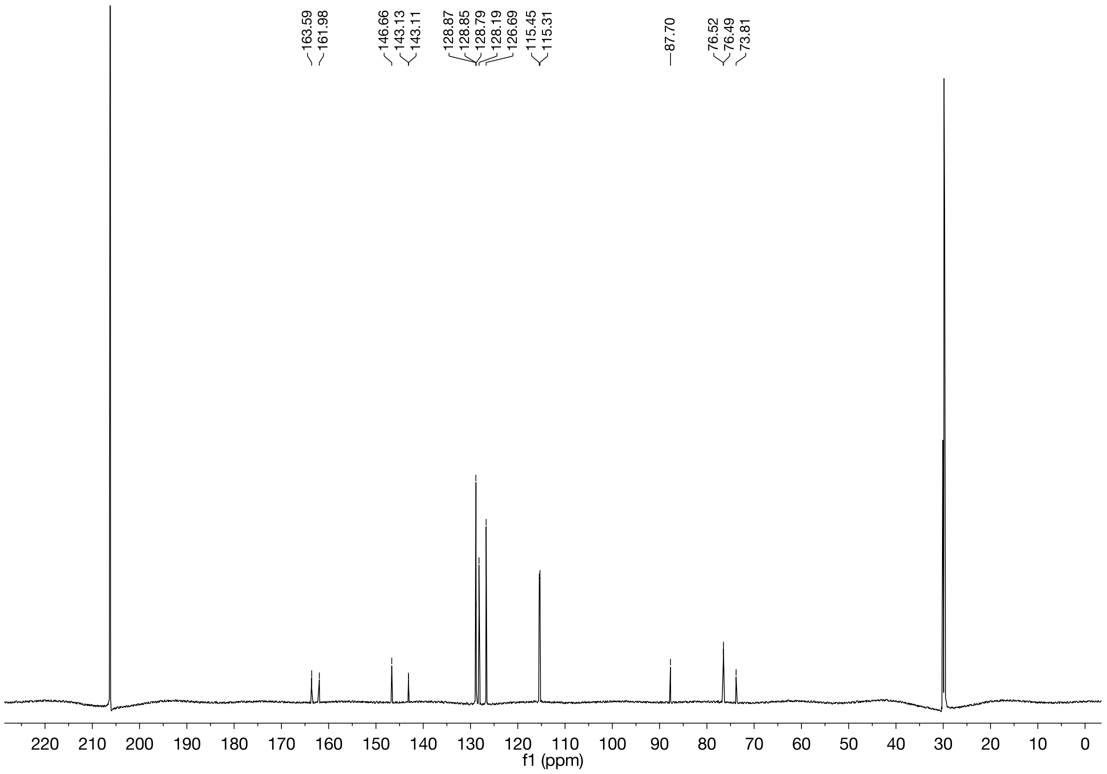

^{19}F NMR of 3i

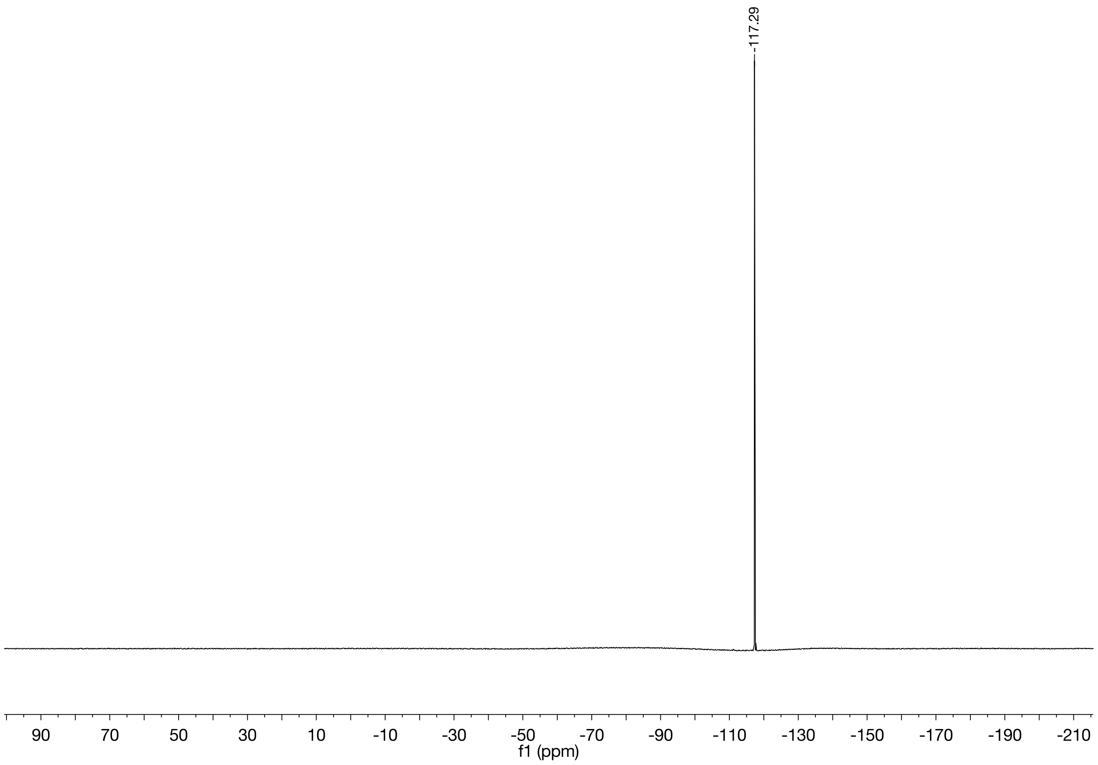

^{1}H NMR of 2j

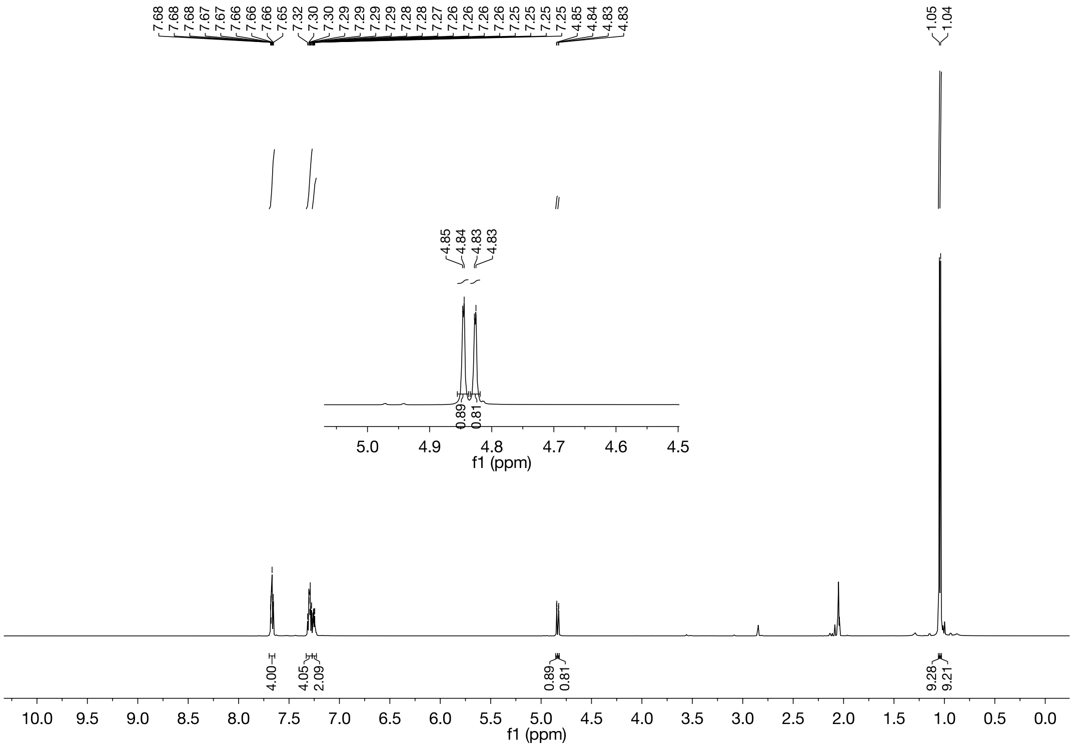

^{13}C NMR of 2j

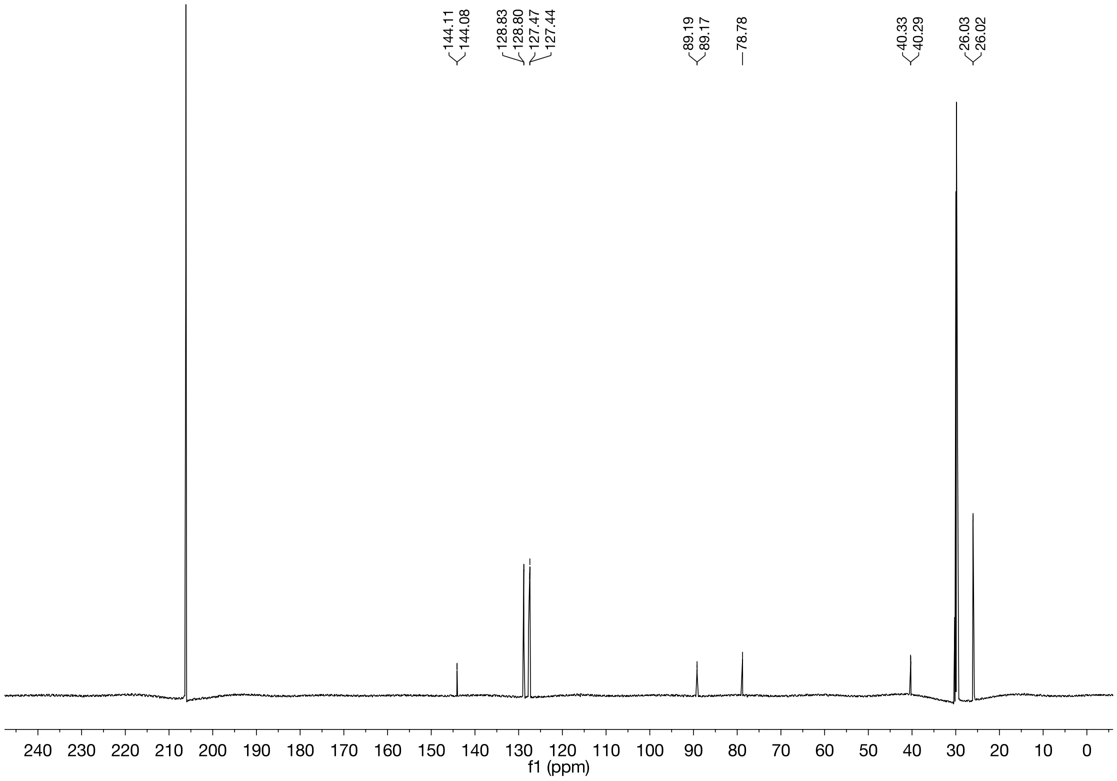

^{1}H NMR of 3j

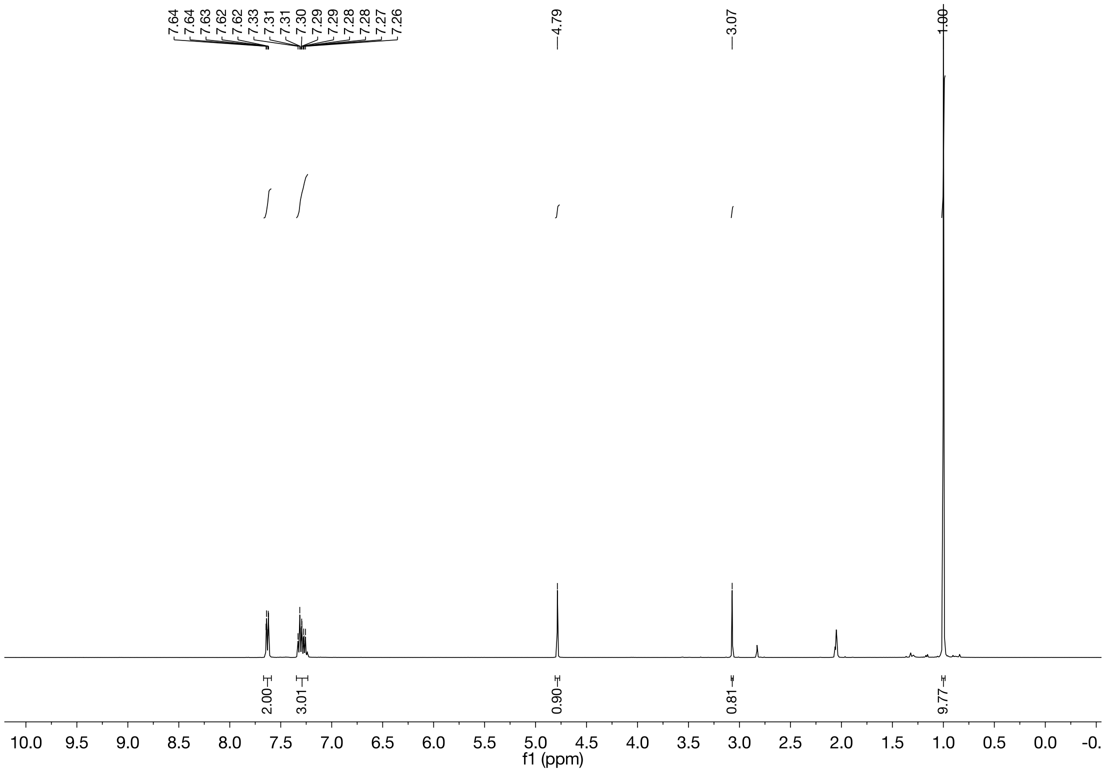

^{13}C NMR of 3j

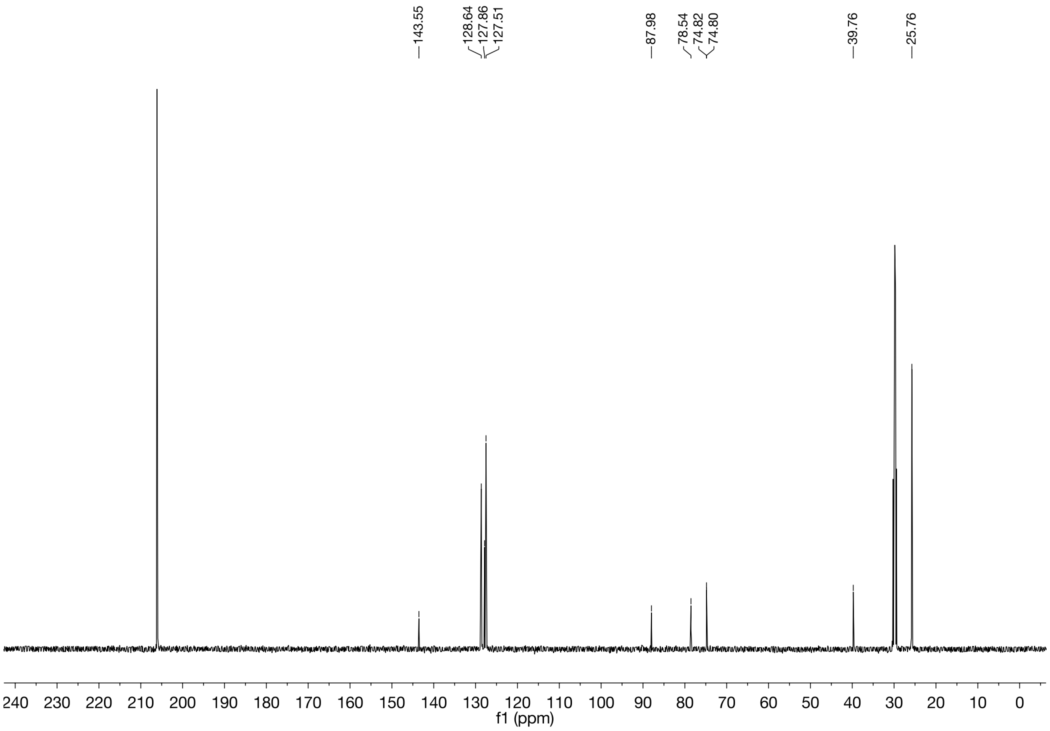

^{1}H NMR of 4a

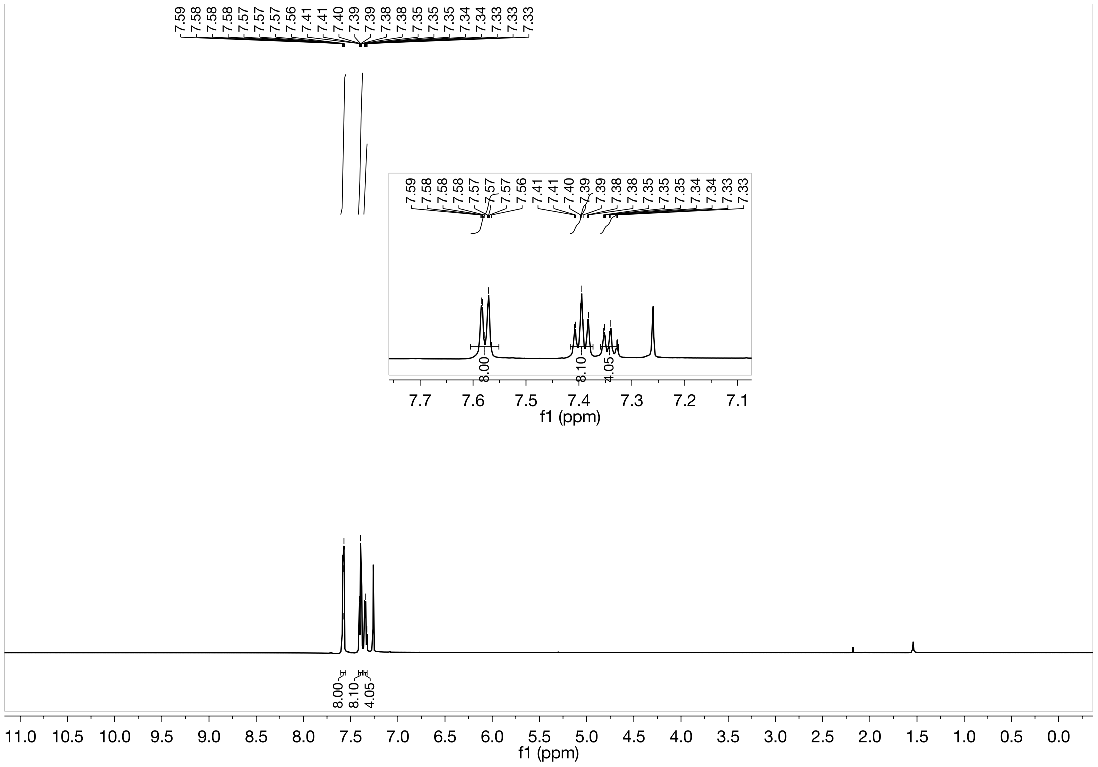

^{13}C NMR of 4a

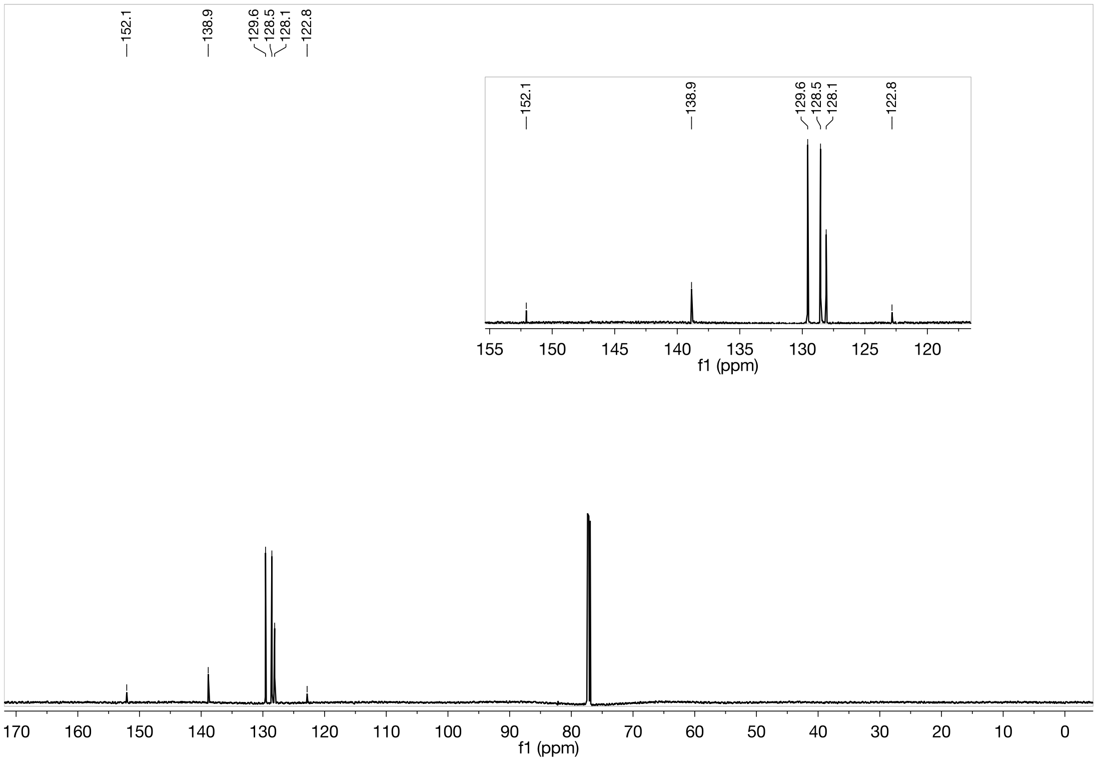

^{1}H NMR of 4a'

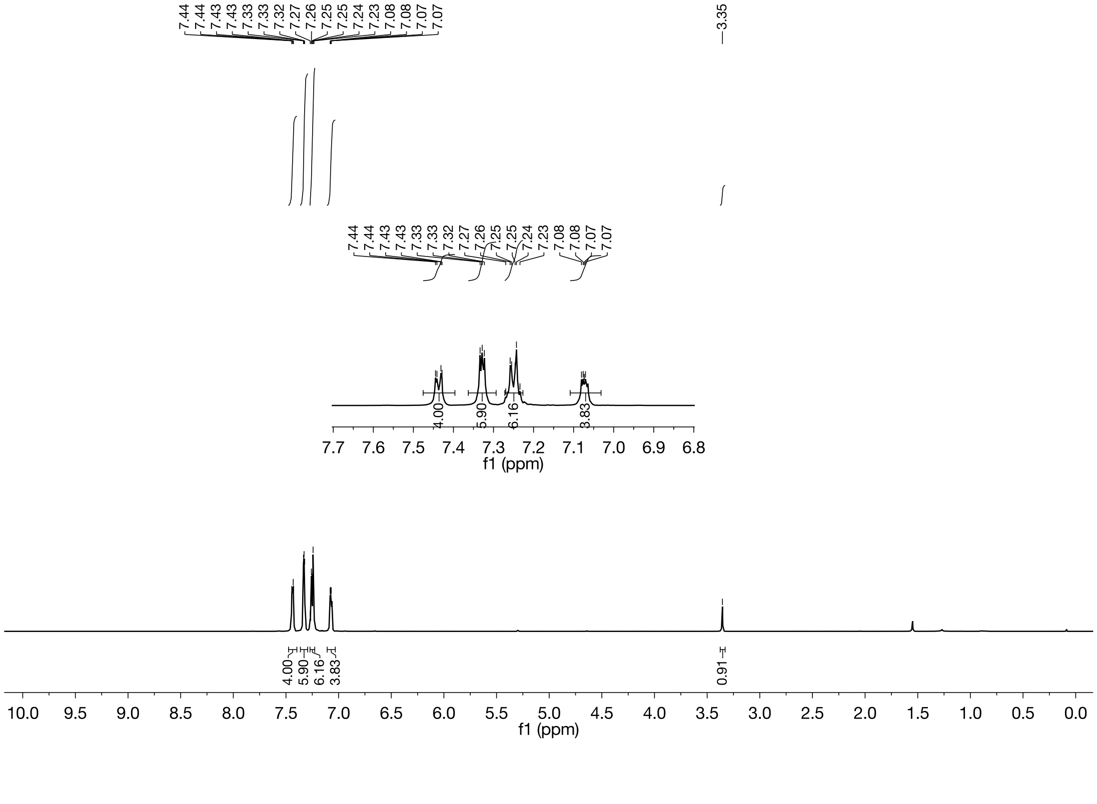

^{13}C NMR of 4a'

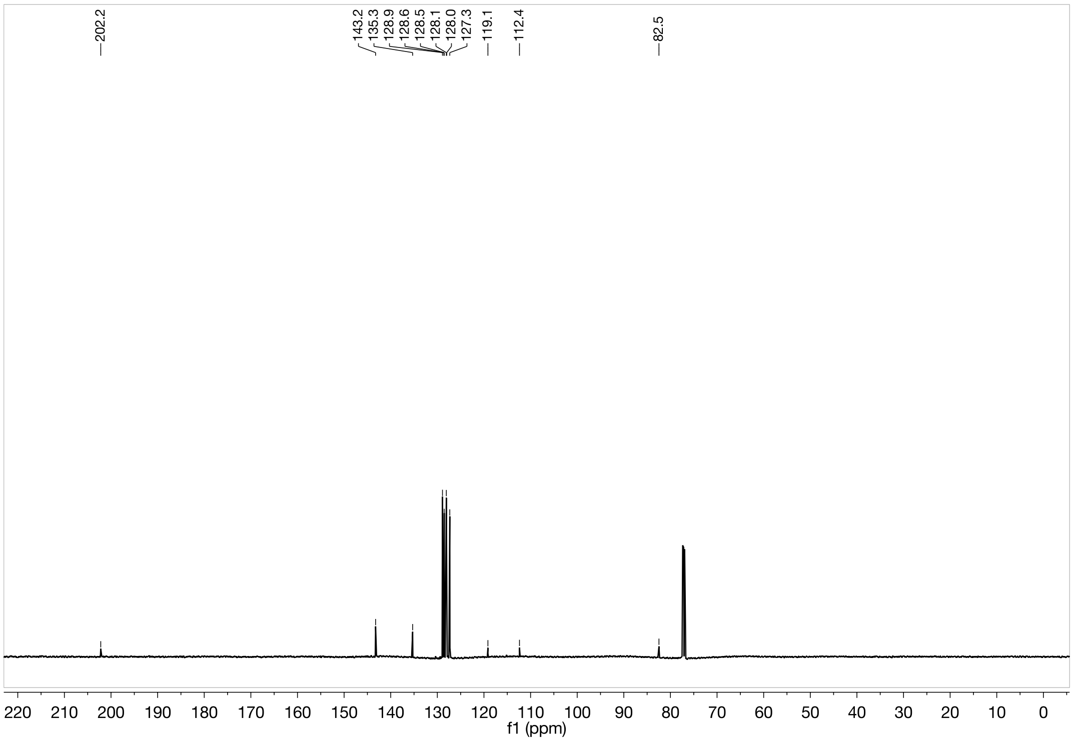

^{1}H NMR of 4b

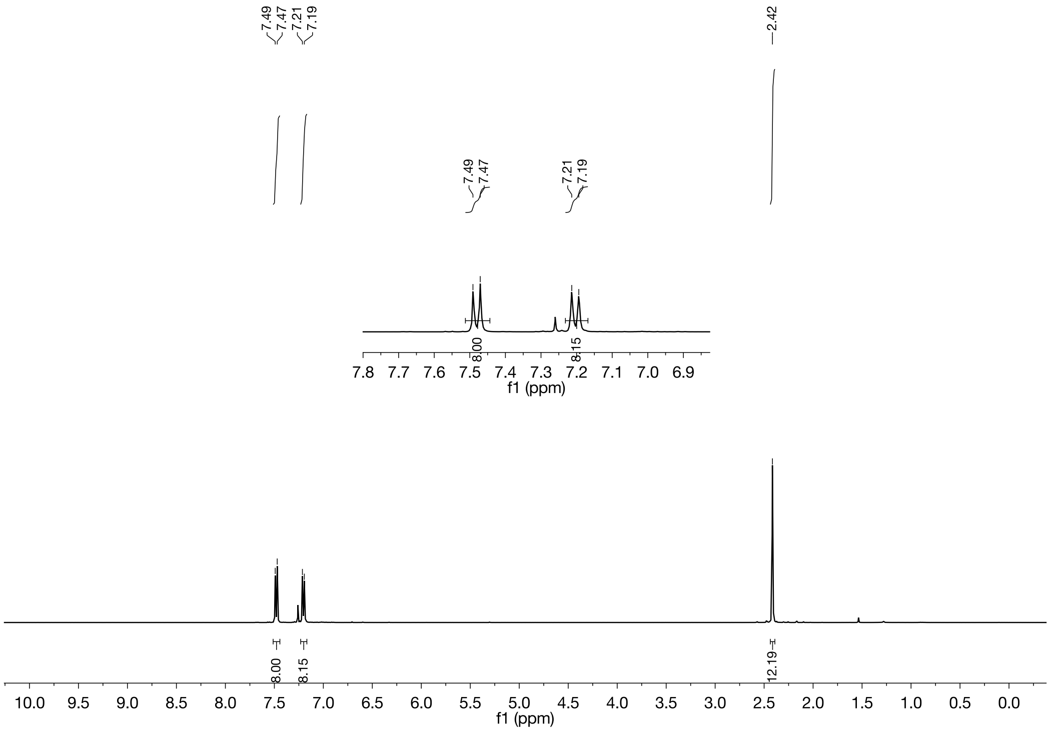

^{13}C NMR of 4b

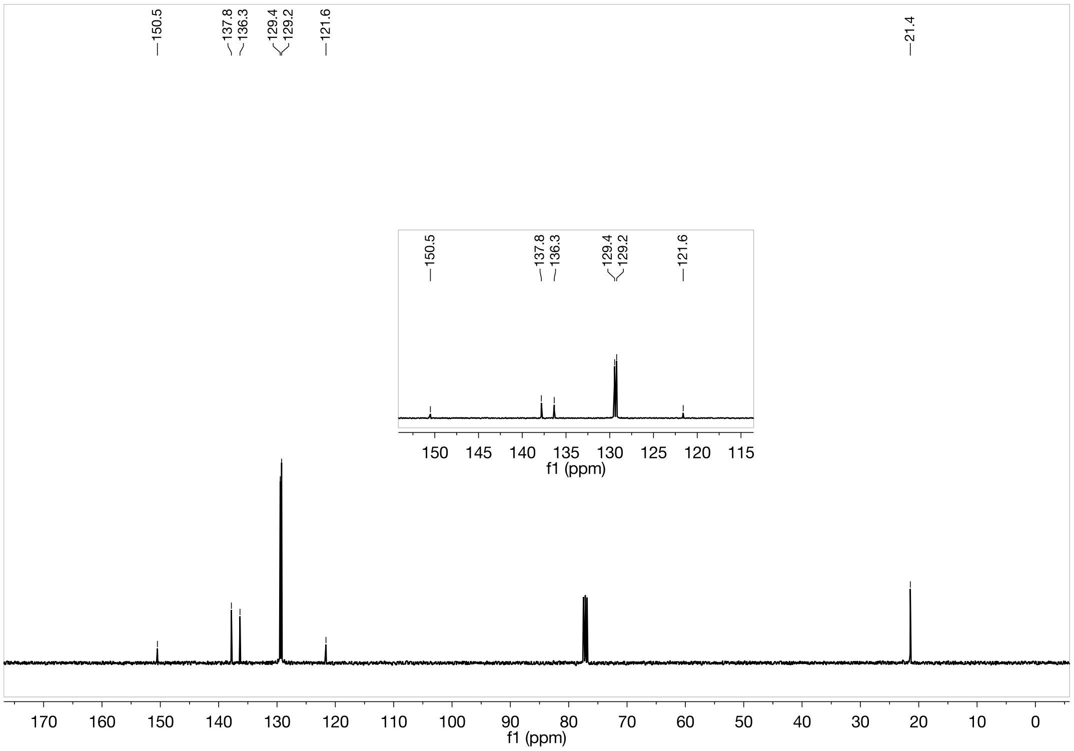

^{1}H NMR of 4d

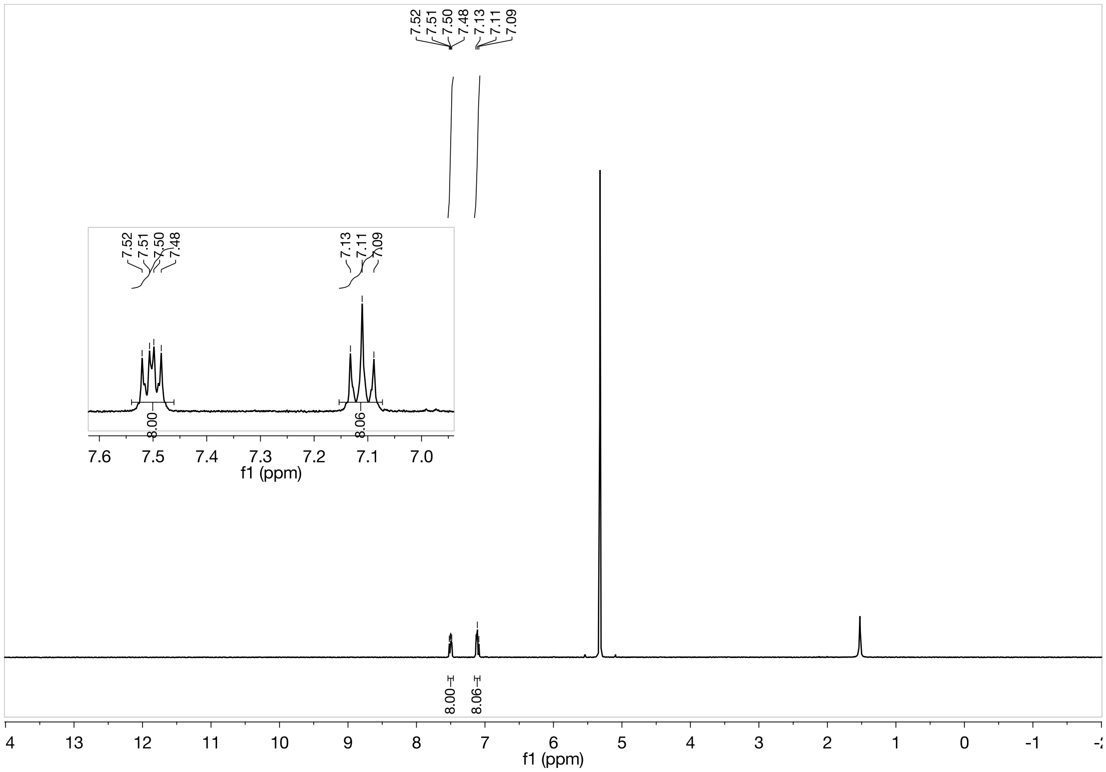

^{13}C NMR of 4d

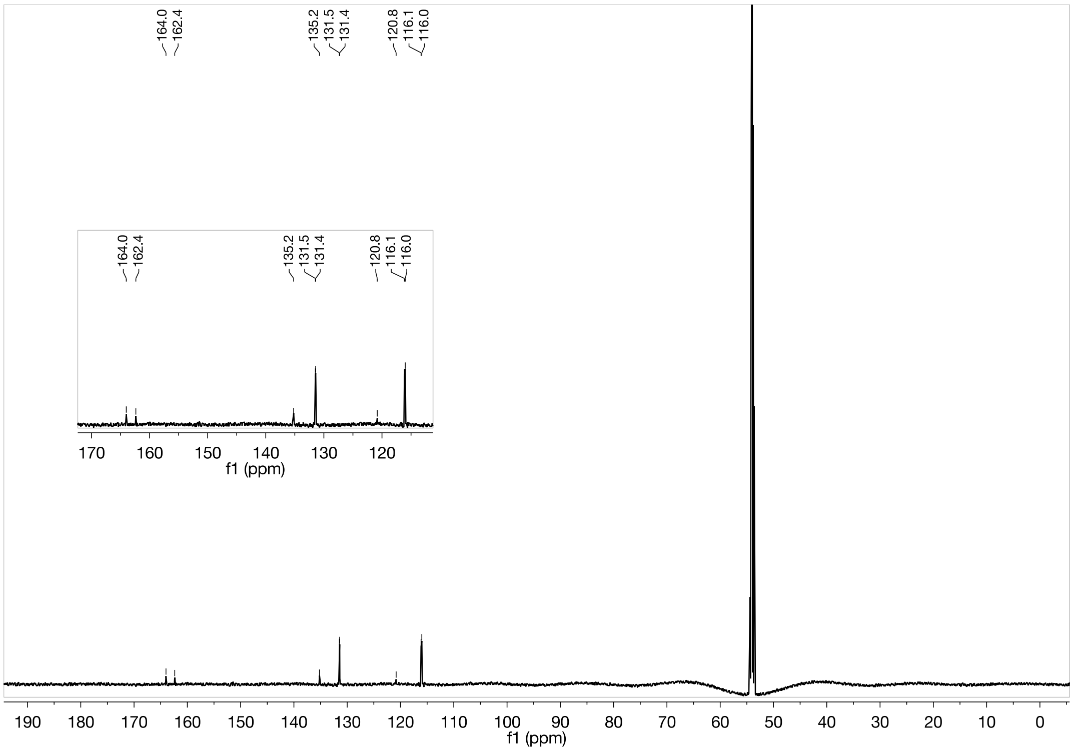

^{19}F NMR of 4d

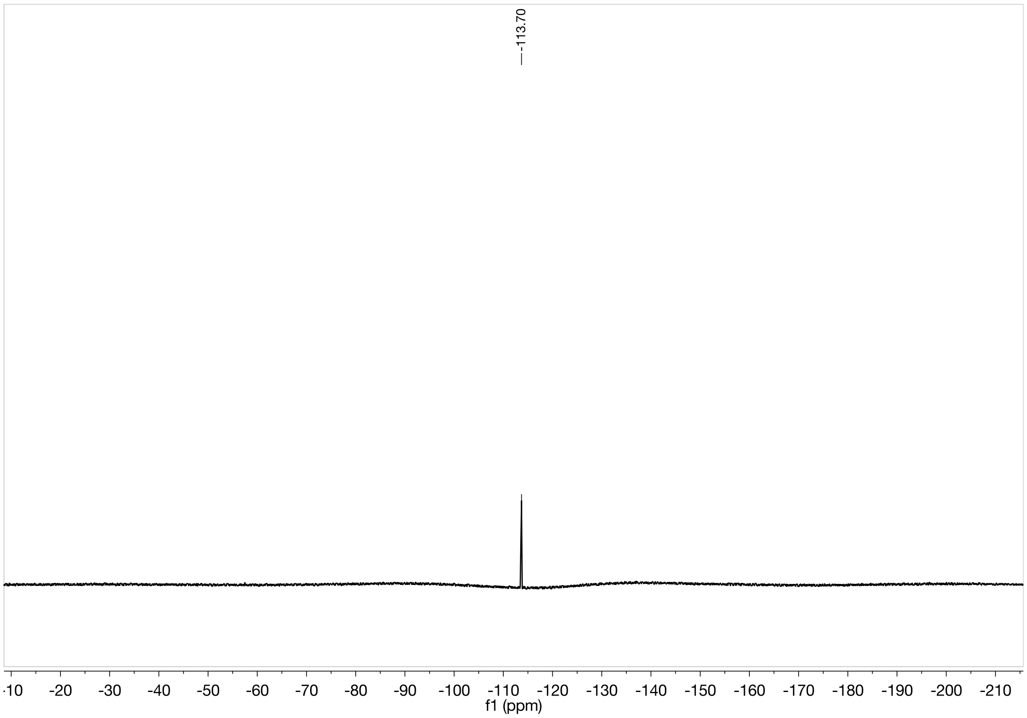

^{1}H NMR of 4i

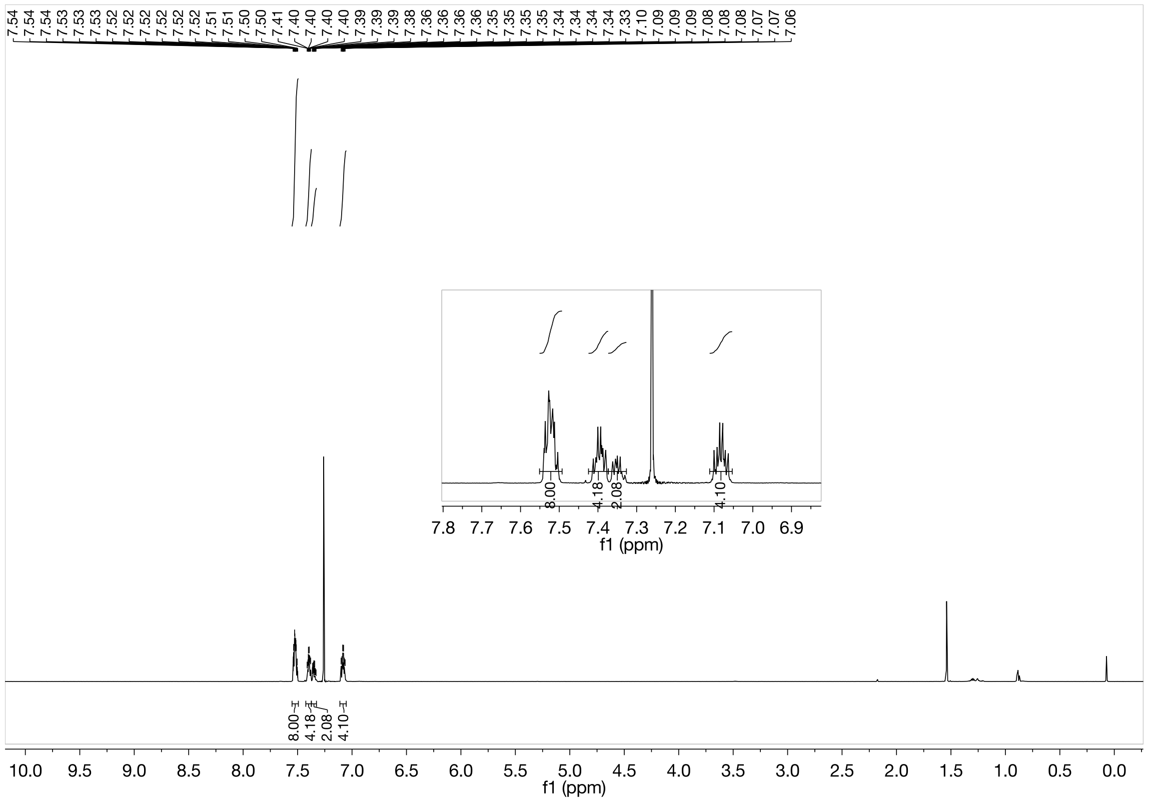

^{13}C NMR of 4i

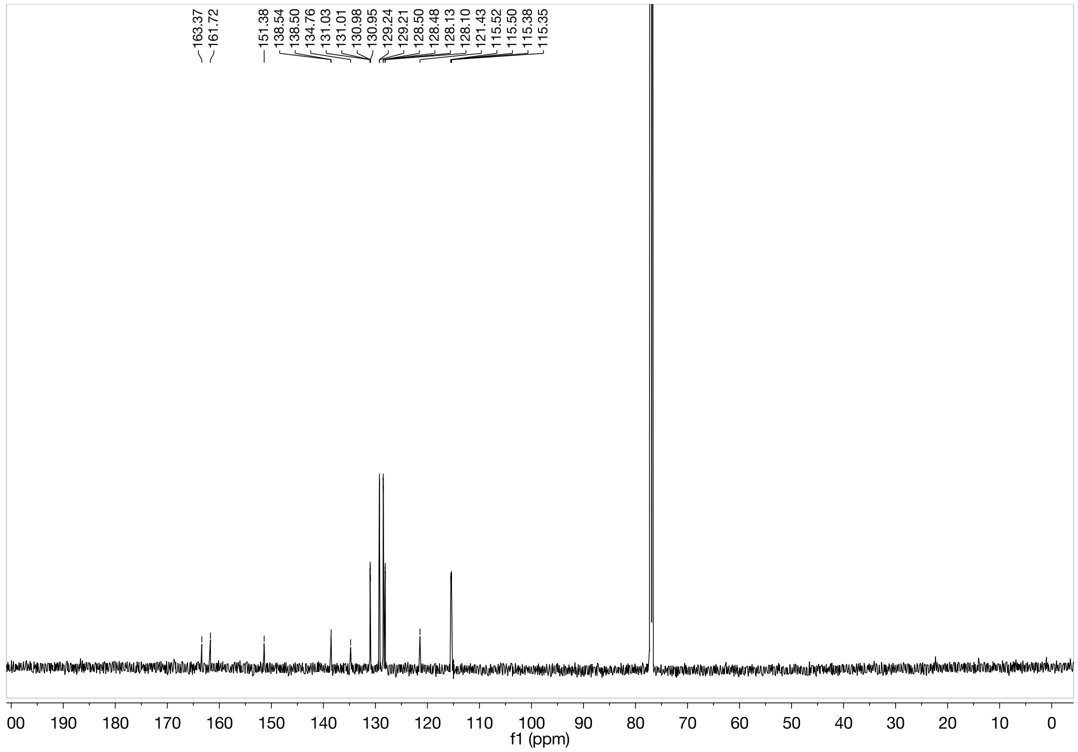

^{19}F NMR of 4i

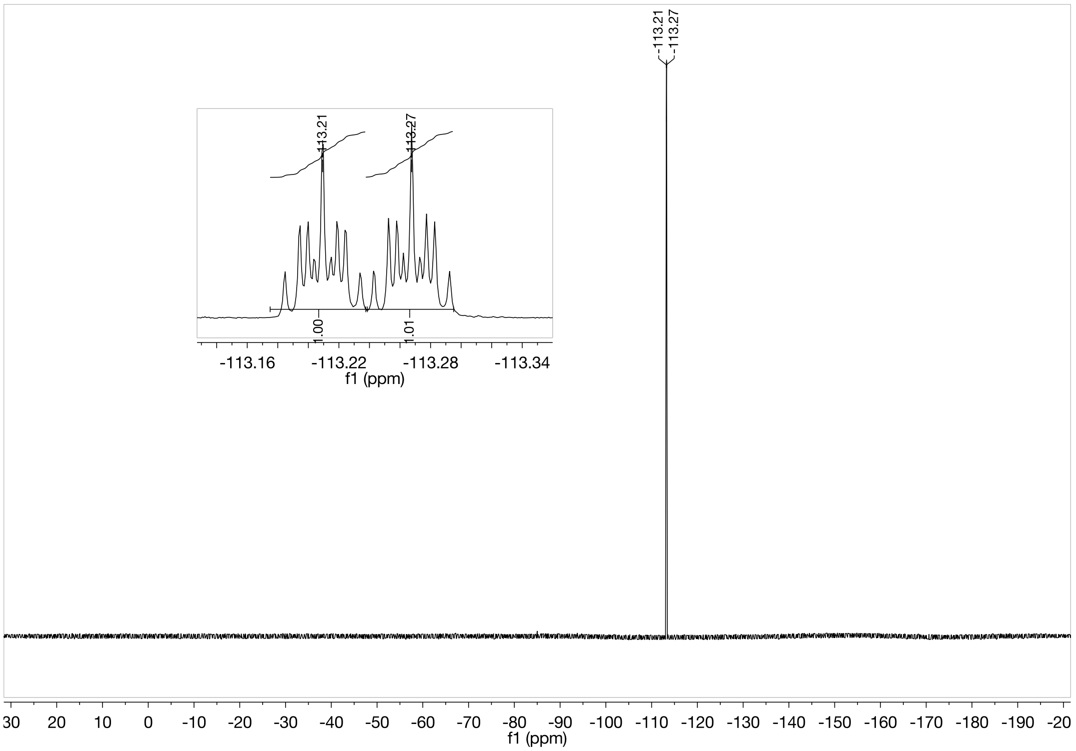

^{1}H NMR of 5a

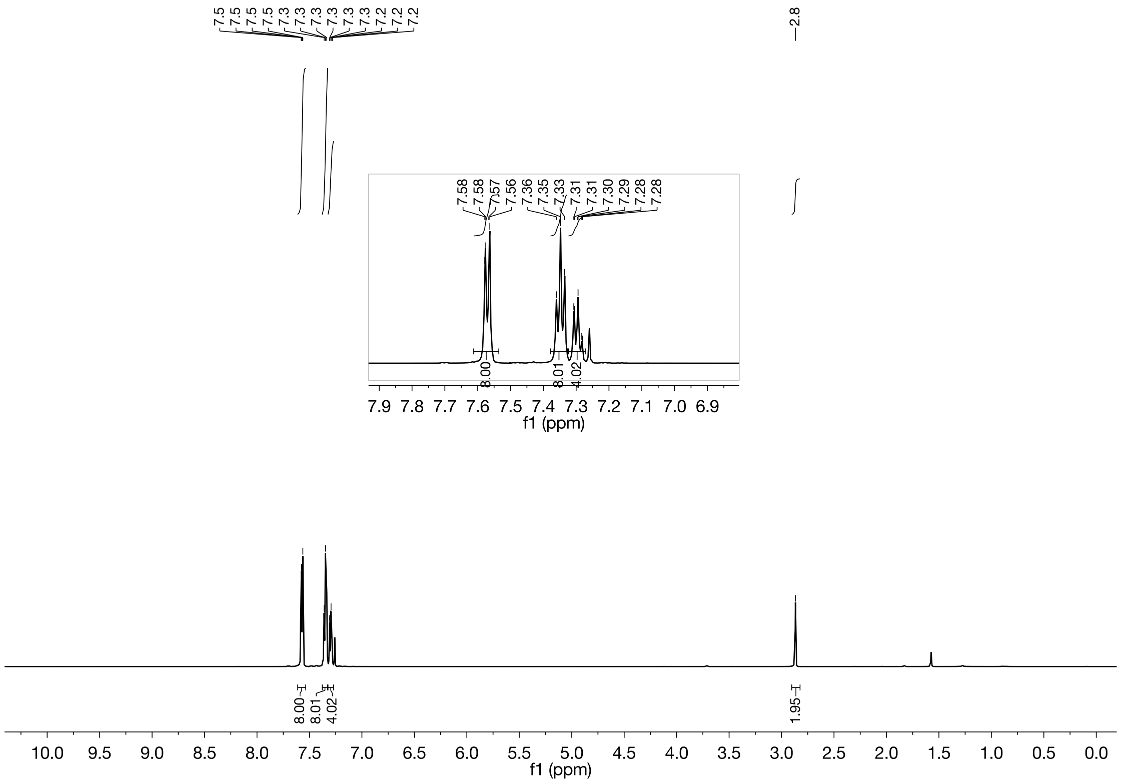

13C NMR of 5a

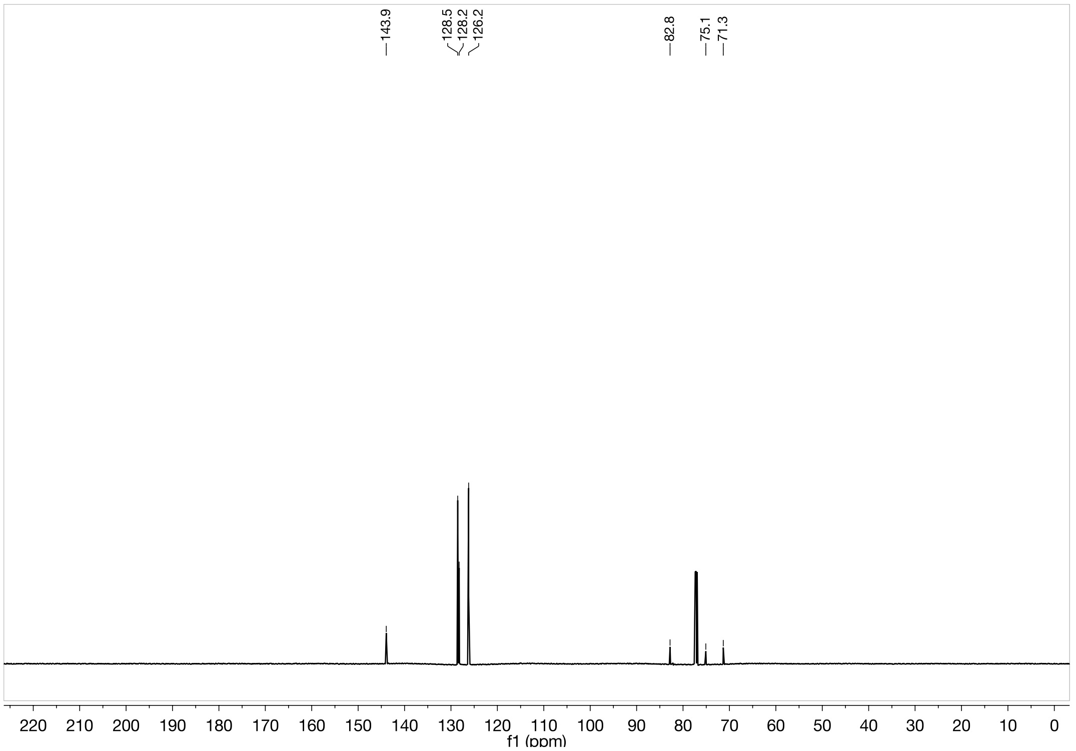

1H NMR of 6a

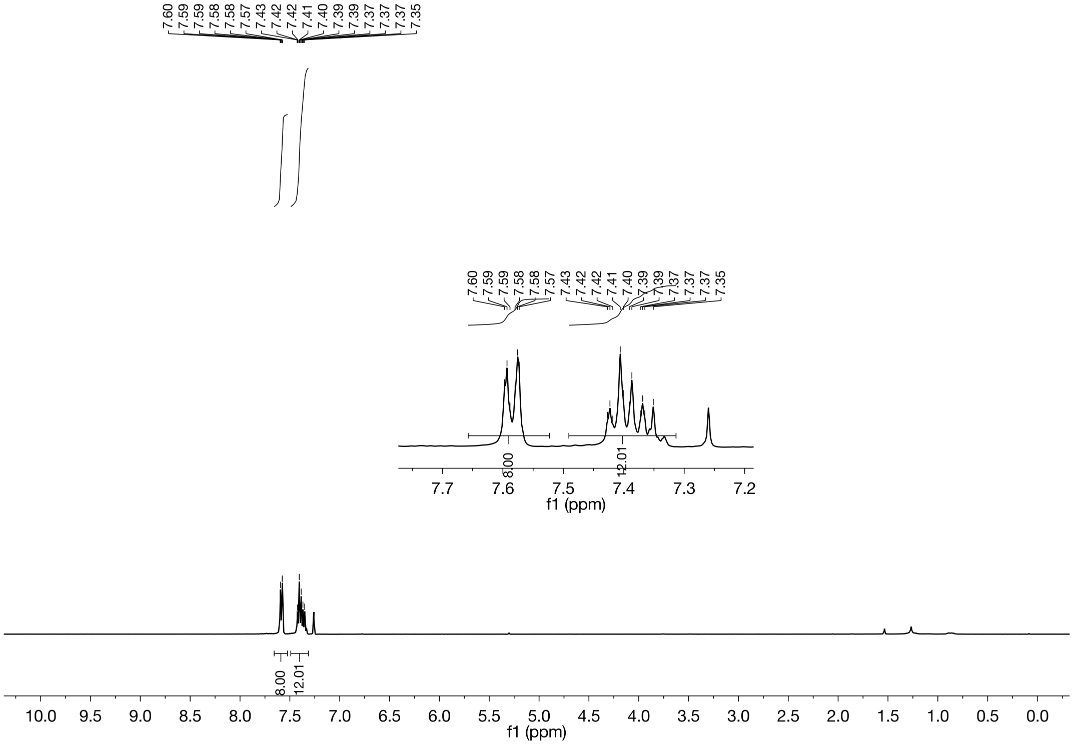

^{13}C NMR of 6a

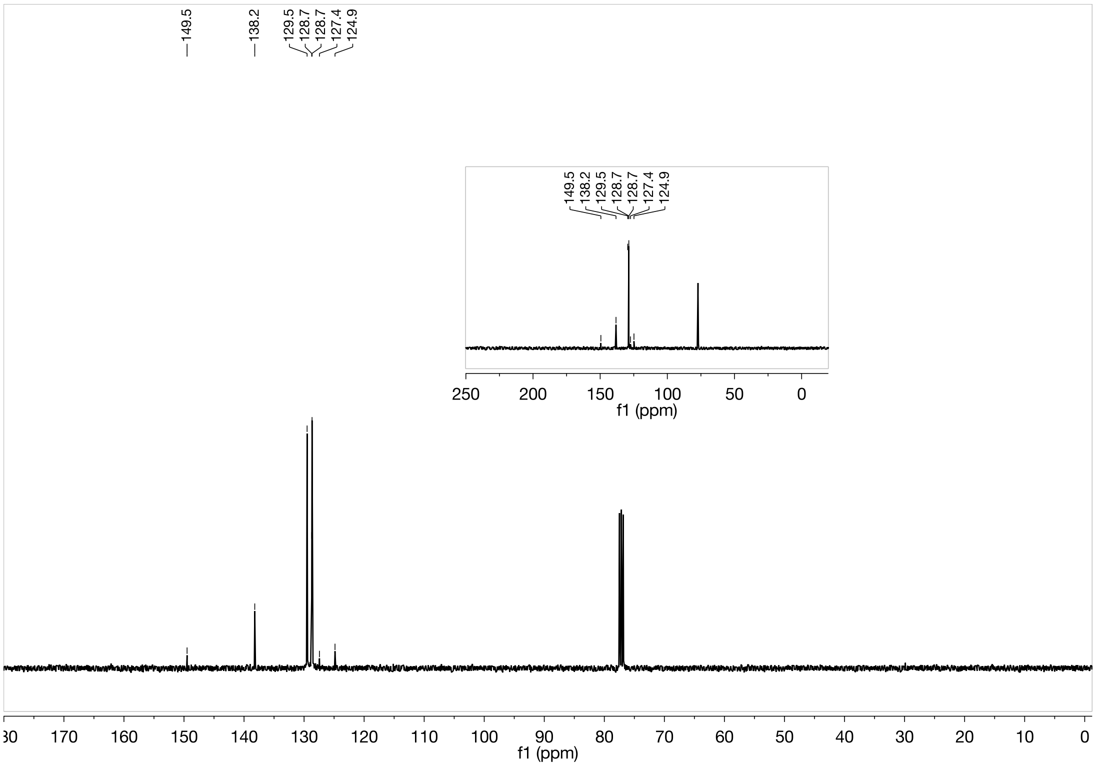

9. References

1 J. K. Sundar, K. M. Kumar, V. Vijayakumar, J. Suresh, S. Natarajan, P.L N. Lakshman, *Acta Crystallogr*. **2010**, *E66*, o679.

2 V. G. Jiménez, R. Tapia, M. A. Medel, I. F. A. Mariz, T. Ribeiro, V. Blanco, J. M. Cuerva, E. Maçôas, A. G. Campaña, *Chem. Commun*. **2018**, *54*, 3359–3362.

3 N. Kerisit, P. Gawel, B. Levandowski, Y.-F. Yang, V. García-López, N. Trapp, L. Ruhlmann, C. Boudon, K. N. Houk, F. Diederich, *Chem. Eur. J*. **2018**, *24*, 159–168.

4 M. Mondon, R. Delatouche, C. Bachmann, G. Frapper, C. Len, P. Bertrand, *Eur. J. Org. Chem*. **2011**, 2011–2119.

5 P. Gawel, C. Dengiz, A. D. Finke, N. Trapp, C. Boudon, J.- P. Gisselbrecht, F. Diederich, *Angew. Chem. Int. Ed*. **2014**, *53*, 4341–4345.

6 C. Böttcher, G. Zeyat, S. A. Ahmed, E. Irran, T. Cordes, C. Elsner, W. Zinth, K. Rueck-Braun, *Beilstein J. Org. Chem*. **2009**, *5*, 25.

7 J. N. Moorthy, A. L. Koner, S. Samantha, A. Roy, W. M. Nau, *Chem. Eur. J*. **2009**, *15*, 4289–4300.

8 H. Cho, I. Kim, *Tetrahedron,* **2012**, *68*, 5464–5480.

9 F. Toda, N. Ooi, K. Akagi, *Bull. Chem. Soc. Jpn.* **1971**, *44*, 1050–1054.

10 Y. Kuwatani, G. Yamamoto, M. Oda, M. Iyoda, *Bull. Chem. Soc. Jpn*. **2005**, *78*, 2188–2208.
